# Scutellarin Rescued Mitochondrial Damage through Ameliorating Mitochondrial Glucose Oxidation via the Pdk‐Pdc Axis

**DOI:** 10.1002/advs.202303584

**Published:** 2023-09-26

**Authors:** Ning Sheng, Zhihui Zhang, Hao Zheng, Congyu Ma, Menglin Li, Zhe Wang, Lulu Wang, Jiandong Jiang, Jinlan Zhang

**Affiliations:** ^1^ State Key Laboratory of Bioactive Substance and Function of Natural Medicines Institute of Materia Medica Chinese Academy of Medical Science and Peking Union Medical College Beijing 100050 China; ^2^ Institute of Medicinal Biotechnology Chinese Academy of Medical Science and Peking Union Medical College Beijing 100050 China

**Keywords:** ^13^C metabolic flux analysis, mitochondria, proteomics, pyruvate dehydrogenase kinase, scutellarin

## Abstract

Mitochondrial bioenergetic deficits and their resulting glucose hypometabolism are the key pathophysiological modulators that promote neurodegeneration. However, there are no specific potential molecules that have been identified to treat neurological diseases by regulating energy metabolism and repairing mitochondrial damage. Pyruvate dehydrogenase (PDH) complex (PDC), which can be phosphorylated by pyruvate dehydrogenase kinase (PDK), is the gate‐keeping enzyme for mitochondrial glucose oxidation. In this study, a small‐molecule scutellarin (SG) is discovered that can significantly alleviate the neuropathological changes in hippocampal CA1 of cerebral hypoperfusion model rats, rescued the morphological changes of abnormal mitochondria, and restored mitochondrial homeostasis. Mitochondrial proteomics, energy metabolism monitoring, and ^13^C‐metabolic flux analysis targeted SG activity on PDK2, thus regulating PDK‐PDC‐mediated glycolytic metabolism to TCA cycle during mitochondrial OXPHOS damage. The knockdown of PDK2 in the SK‐N‐SH cells validated that SG could rescue mitochondrial damage via the PDK‐PDC axis, promote the MMP level and reduce the mitochondria‐dependent apoptosis. Collectively, this study explored the novel therapeutic approach: the PDK‐PDC axis for neurological injury and cognitive impairment and uncovered the effect of SG on mitochondrial protection via the PDK‐PDC axis and mitochondrial glucose oxidation. The findings indicate that active components ameliorating mitochondrial bioenergetic deficits could be of significant value for neurological disease therapy.

## Graphical abstract

This study explores the novel therapeutic approach: the PDK‐PDC axis for neurological injury and cognitive impairment and uncoveres the effect of SG on mitochondrial protection via the PDK‐PDC axis and mitochondrial glucose oxidation. The findings indicate that active components ameliorating mitochondrial bioenergetic deficits could be of significant value for neurological disease therapy.

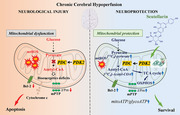

## 1 Introduction

Many lines of evidence suggest that mitochondria have a central role in neurodegenerative disease.^[^ ^1^, ^2^, ^3^ ^]^ Especially, mitochondria abnormalities in nerve disease had been widely studied in recent years and mitochondrial dysfunction has been considered as a final common pathogenic mechanism in aging, Alzheimer's disease (AD), and vascular dementia (VaD).^[^ ^4^, ^5^, ^6^, ^7^ ^]^ These different neurodegenerative diseases share two important characteristics: first, systemic loss of the neurons in the motor, sensory, and cognitive systems leading to cognitive disabilities, such as dementia and motor decline; and second, a correlation between energy metabolic changes and neurodegeneration.^[^ ^8^ ^]^ Given the limited regenerative ability of neuronal tissue, it is important to restrict neuronal impairment and death. As deficits in glucose availability and mitochondrial function are well‐known hallmarks of many neurodegenerative diseases, it appears reasonable to hypothesize that the high energy demand of the brain renders it sensitive to changes in the energy fuel supply and mitochondrial function.^[^ ^9^ ^]^ Therefore, it is of primary significance to interpret the neuronal damage in light of the metabolic changes and to look for therapies that can remedy the energy supply of the mitochondria.^[^ ^10^, ^11^, ^12^, ^13^, ^14^ ^]^ Chronic cerebral hypoperfusion (CCH) plays an important role in the initiation and progression of various neurological diseases.^[^ ^15^, ^16^ ^]^ Nowadays, CCH is increasingly common owing to an increasing burden of vascular risk factors and aging populations. In essence, hypoperfusion deprives the brain of its two paramount trophic substances, viz., oxygen and nutrients. Consequently, the brain suffers from dysfunctional energy metabolism, compromised mitochondrial ATP production, synaptic dysfunction, and neuronal degeneration/loss, leading to cognitive impairment, and AD.^[^ ^15^ ^]^ Among the pathophysiological processes, dysfunctional mitochondrial bioenergetics is associated with the occurrence and progression of CCH and leads to subsequent neuropathological changes.^[^ ^16^ ^]^

Scutellarin (4′,5,6‐hydroxy‐flavone‐7‐glucuronide, SG), a flavonoid glucuronide isolated from *Erigeron breviscapus* (Vant.) Hand.‐Mazz. has been shown to have a wide range of pharmacological activities and clinical applications in treating cerebrovascular and cardiovascular diseases.^[^ ^17^ ^]^ Recently, SG has been found to have potential therapeutic effects on neurological diseases, including neurodegenerative diseases.^[^ ^18^, ^19^, ^20^ ^]^ Importantly in these studies, our group found that SG could regulate neurotransmitter metabolism in GABAergic and glutamatergic neurons.^[^ ^21^ ^]^ The neurotransmitter metabolism is closely linked to the energy metabolism of nerve cells, which is extensively disturbed in AD.^[^ ^22^ ^]^ Since the function of nerve cells and brain tissue are extremely dependent on mitochondrial function, the roles of SG on cellular energy metabolism during bioenergetic deficits need to be thoroughly investigated.

Considering the key factor of mitochondria‐mediated neuronal damage and cognitive impairments in CCH, the present study is designed to investigate the protective mechanisms of SG on CCH through alleviating mitochondria damage. Global proteome profiled mitochondrial dysfunction of CCH and SG effect on mitochondrial aerobic metabolism. The real‐time cellular bioenergetics analysis verified the effect of scutellarin on mitochondrial aerobic respiration. Pyruvate dehydrogenase (PDH) complex (PDC), which could be phosphorylated by pyruvate dehydrogenase kinase (PDK), is the gate‐keeping enzyme for mitochondrial glucose oxidation. We performed a ^13^C‐labeled metabolic flux analysis that revealed SG could significantly enhance the flux of [^13^C_3_]‐pyruvate to [^13^C_2_]‐acetyl‐CoA, thereby enhancing mitochondrial glucose source aerobic respiration. After that, a series of experiments were conducted, including activity‐based kinase evaluation, molecular docking, co‐IP, Lip‐MS, DARTS, CETSA, etc., uncovered that SG could selectively inhibit the enzymatic activity of PDK2 as the pharmacological target, thus regulating PDK‐PDC‐mediated pyruvate metabolism. Finally, we conducted the cell line of stable knockdown of PDK2 to verify SG's functional act on PDK2 for mitochondrial protection. The results showed that SG rescued mitochondrial aerobic respiration, reversed mitochondrial membrane potential loss, and reduced the apoptosis induced by mitochondrial damage via PDK‐PDC axis. In summary, we revealed that SG, with PDK2 as the pharmacological target, was a novel molecule template for regulating mitochondrial damage by acting on mitochondrial glucose oxidation. Moreover, PDK‐PDC axis may represent a promising pharmacological therapeutic target of cerebral ischemia leading to neurological disease, especially with cognitive impairment.

## 2 Results

### 2.1 Scutellarin Ameliorated CCH Induced Brain Pathophysiological Changes and Cognitive Impairment

**Figure 1:**
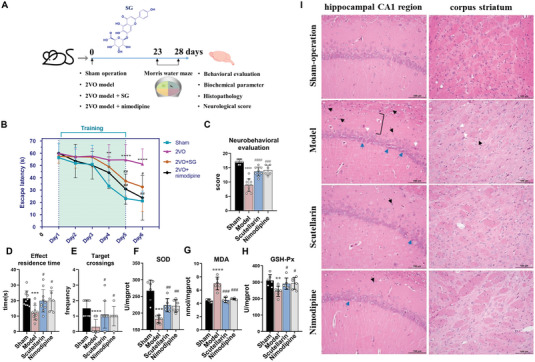
SG could alleviate pathological changes caused by chronic cerebral ischemia and improve cognitive impairment and reduce oxidative stress. A) The process to evaluate the stability of the 2VO model and the curative potential of SG. B) The escape latency of the water maze experiment. C) Neurobehavioral evaluation results. D) The effect residence time of the water maze experiment. E) The target crossings of the water maze experiment. FH) Evaluation of oxidative stress‐related parameters among the groups. I) The histopathology of the brain tissue among the groups (the black arrows represent inflammatory microglia; the white arrows represent shrinkage of neurons; the blue arrow represents cell pycnosis). Data are presented as mean ±SEM by paired two‐tailed Student's *t*‐test, ^*^ *p* < 0.05, ^**^ *p* < 0.01, ^***^ *p* < 0.001, ^****^ *p* < 0.0001, model versus control group, ^#^ *p* < 0.05, ^##^ *p* < 0.01, ^###^ *p* < 0.001, ^####^ *p* < 0.0001, SG or nimodipine versus model group.

Increasing evidence suggests that vascular risk factors contribute to neurodegeneration, cognitive impairment, and dementia.^[^ ^23^ ^]^ While there is considerable overlap between features of vascular cognitive impairment and dementia (VCID) and Alzheimer's disease (AD), it appears that cerebral hypoperfusion is the common underlying pathophysiological mechanism that is a major contributor to cognitive decline and degenerative processes leading to dementia.^[^ ^14^ ^]^ The reconstruction of the CCH pathological condition in animal models is a suitable approach to the unraveling of causal relationships. For this reason, permanent, bilateral occlusion of the common carotid arteries (2VO) in rats has been established as a procedure to investigate the effects of CCH on cognitive dysfunction and neurodegenerative processes, with nimodipine as a positive control. The 2VO model has generated a large amount of data, revealing the 2VO‐related pattern of cerebral hypoperfusion and metabolic changes, learning and memory disturbances, failure of neuronal signaling, and neuropathological changes in the hippocampus.^[^ ^24^ ^]^ The water maze experiment, oxidative stress test, and histopathological analyses were applied to evaluate the stability of the 2VO model and the curative potential of SG (**Figure** **1**A). Neurobehavioral scoring was performed using the modified Garcia JH method.^[^ ^25^ ^]^ The evaluation index of the water maze experiment, including escape latency, effect residence time and target crossings, and neurobehavioral scoring showed both the sham‐operation group and SG group were significantly superior to the model group, indicating that SG could reduce cognitive impairment in ischemic brain tissue (Figure 1B–E). Hemagglutinin‐eosin staining (HE) showed that the hippocampal CA1 and corpus striatum region of the model group exhibited typical neuropathological changes, appeared significant cell pycnosis, shrinkage, and staining loss (marked with arrows in Figure 1I). By contrast, these characteristics substantially improved in the SG group. The results of the biochemical parameter analysis were shown in Figure 1F–H. Compared with the sham‐operation group, SOD and GSH‐px were significantly lower in the model group and significantly increased after SG treatment. On the contrary, MDA presented an opposite trend. The results of these biochemical indicators showed that SG had an effect on regulating oxidative stress injury of 2VO model rats. These results basically indicated the pharmacodynamic effect of SG on CCH. Essentially, hypoperfusion results in the deprivation of the brain's two paramount trophic substances: oxygen and nutrients.^[^ ^14^ ^]^ Brain hypoperfusion, glucose hypometabolism, and diminished energy supply were directly related to mitochondria.^[^ ^15^ ^]^ The focus of our study was thus directed toward the regulation of mitochondrial dysfunction by SG.

### 2.2 Scutellarin Rescued CCH Induced Mitochondrial Dysfunction

**Figure 2:**
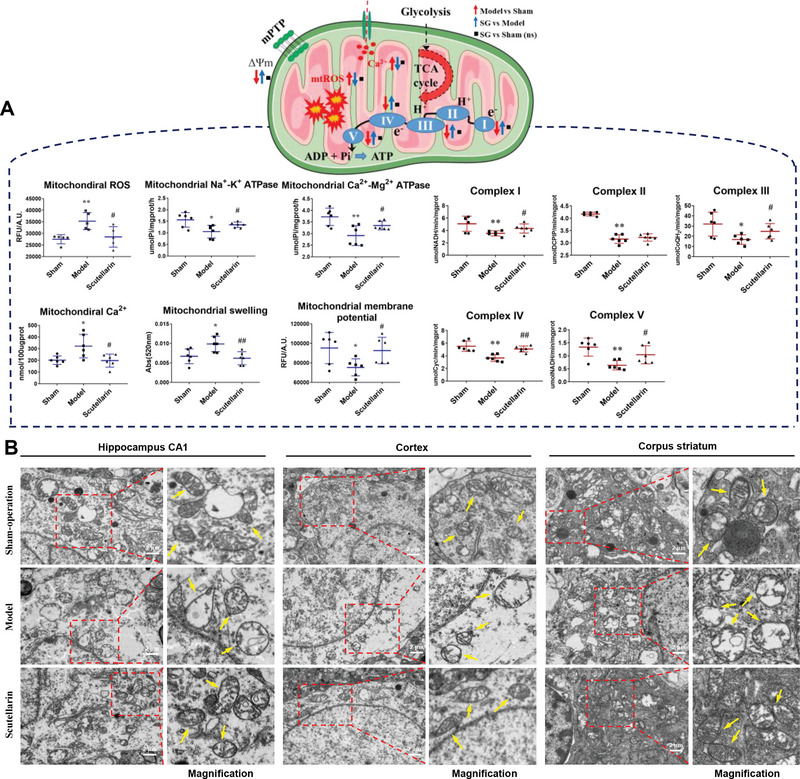
Scutellarin rescued CCH‐induced mitochondrial dysfunction. A) SG regulated mitochondria homeostasis and ATP production. B) Ultrastructural changes of mitochondria in hippocampal CA1, cortex, and corpus striatum by TEM. Data are presented as mean ±SEM by paired two‐tailed Student's *t*‐test, ^*^ *p* < 0.05, ^**^ *p* < 0.01, ^***^ *p* < 0.001, ^****^ *p* < 0.0001, model versus control group, ^#^ *p* < 0.05, ^##^ *p* < 0.01, ^###^ *p* < 0.001, ^####^ *p* < 0.0001, SG versus model group.

Mitochondria have distinct functions for cellular homeostasis in regulating bioenergy, biosynthesis, and signaling. Reduction in ATP production leads to compromised function of ATP‐dependent ion channels such as the Na^+^/K^+^ and Ca^2+^ pump.^[^ ^26^ ^]^ Consequently, this increases the resting membrane potential to the threshold, leading to unregulated depolarizations known as anoxic depolarization in neurons. The level of mitochondrial ROS, a critical factor in oxidative stress, was quantified and depicted in **Figure** **2**A. The results demonstrated that model rats exhibited a significant increase compared to sham‐operated rats, while SG treatment significantly reduced ROS levels relative to the model group. Notably, there was no significant difference in mitochondrial ROS levels between SG and sham‐operated group, indicating that SG could effectively alleviate oxidative stress in the CCH state. Meanwhile, evidence indicates that Na^+^‐K^+^ ATPase and Ca^2+^‐Mg^2+^ ATPase are most sensitive to ROS and thus changes of ATP production, which regulate the ionic concentration gradients in neurons for stimulating the generation of action potentials. The evaluation results showed that mitochondrial Na^+^‐K^+^ ATPase and Ca^2+^‐Mg^2+^ ATPase activities were markedly increased in the SG‐treated and sham‐operation group compared to the 2VO model group.

Considering the possible damage to mitochondria caused by excessive ROS and oxidative stress, we employed a series of assays to spectrofluorometrically measure the situation of mitochondrial permeability transition pore (mPTP) opening, which implicated as a mitochondrial status indicator and cell death pathway. The results demonstrated that mPTP opening was clearly apparent in the model group by the nearly simultaneous release of mitochondrial Ca^2+^ (increase in Rhod‐2 ratio signal), the collapse of mitochondrial membrane potential (decrease in JC‐1 ratio signal), and increased mitochondrial volume (decrease in the swelling signal) relative to sham‐operation group, and the treatment with SG was able to restore these changes significantly (Figure 2A).

ATP production is synthesis from oxidative phosphorylation (OXPHOS) derived by the coupled functioning of the mitochondrial electron transport chain (ETC) and the ATP synthase. The activities of mitochondrial respiratory chain complexes were assessed via spectrophotometry (Figure 2A). Compared to the sham‐operated group, the CCH model group exhibited a significant decrease in complex activity, while treatment with SG resulted in a corresponding increase in the activity of complex I, III, IV, and V compared to the model group. The results suggested that CCH could damage mitochondrial complexes, resulting in decreased energy generation, and SG could enhance the activities of several mitochondrial respiratory chain complexes. Moreover, the mitochondrial respiratory chain is one of the main sites of ROS generation even in physiological circumstances, complexes I and III are the major contributors of free radicals and ROS especially, in agree with the determination of ROS in brain mitochondria, that is SG could decrease robust ROS production and restore mitochondrial respiratory function and ETC complexes activity. Additionally, under physiological conditions, SG does not significantly affect mitochondrial state and there are no significant differences in the parameters compared to the control group (Figure S1, Supporting Information).

We observed ultrastructural changes of mitochondria in hippocampal CA1, cortex, and corpus striatum by transmission electron microscopy (TEM). As shown in Figure 2B, in the sham group, the mitochondria in all samples had prominent cristae and intact membranes, while in the CCH group, the mitochondria were swelled, and the cristae were broken and moved to the surrounding. The administration of SG ameliorated these damages. These results demonstrated that SG significantly reduced CCH‐induced mitochondrial damage by regulating mitochondria homeostasis and ATP production.

### 2.3 Global Proteome Profiled Mitochondrial Dysfunction of CCH and Scutellarin Effect on Mitochondrial Aerobic Respiration

**Figure 3:**
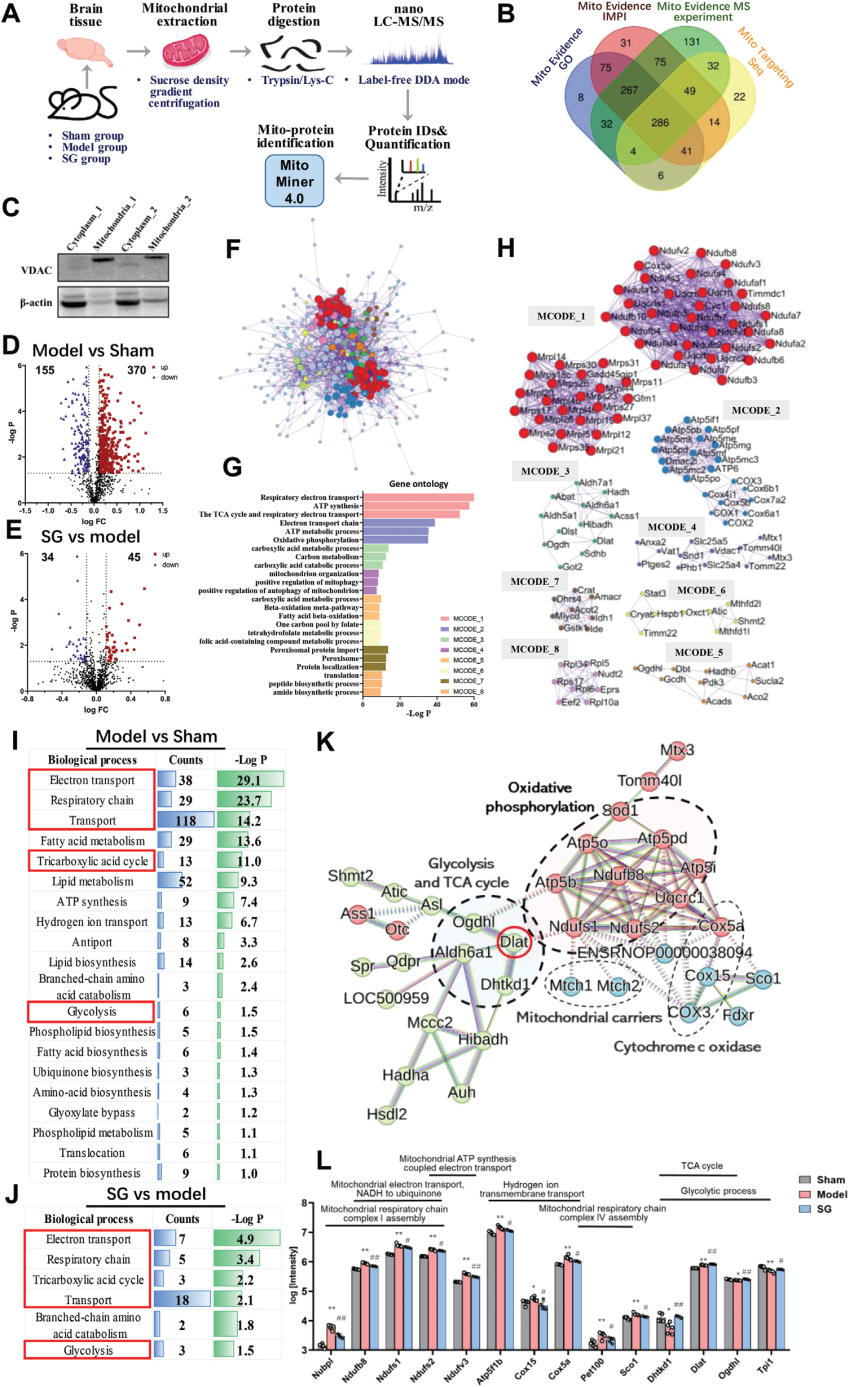
Mitochondrial proteomics revealed the regulatory role of SG on oxidative phosphorylation and energy metabolism pathways. A) The process of mitochondrial proteomics experiment. B) The criteria for mitochondrial protein identification by MitoMiner 4.0. C) WB experiment to verify the purity of extracted mitochondria, VDAC: mitochondrial marker protein, F‐actin, and cytoplasmic marker protein. D) Differentially expressed mitochondrial proteins between model versus sham‐operation group. E) Differentially expressed mitochondrial proteins between SG versus model group. F) Total interaction relationship of the DEMPs. G) The description of the Molecular Complex Detections (MCODEs). H) The DEMPs in each MCODE, of that, MCODE_1 and MCODE_2, contained most of the DEMPs, were both related to OXPHOS and its upstream TCA cycle. I) Function annotation of the differentially expressed mitochondrial proteins between model and sham‐operation group. J) Function annotation of the differentially expressed mitochondrial proteins between model and SG group. K) The PPI enrichment analysis of DEMPs both belong of the model versus sham‐operation group and SG versus model group. L) SG exert reversed regulation of proteins belonging to OXPHOS and its upstream pathways. Data are presented as mean ±SEM by paired two‐tailed Student's *t*‐test, ^*^ *p* < 0.05, ^**^ *p* < 0.01, ^***^ *p* < 0.001, ^****^ *p* < 0.0001, model versus control group, ^#^ *p* < 0.05, ^##^ *p* < 0.01, ^###^ *p* < 0.001, ^####^ *p* < 0.0001, SG versus model group.

Mitochondria serve essential cellular functions, including energy production and anabolic pathways, which is a key feature in both cerebrovascular and cardiovascular diseases.^[^ ^27^ ^]^ In order to unveil mitochondrial dysfunctions and regulating effect of SG, we adopted a discovery‐driven approach with label‐free quantitative proteomics and profiled the mitochondrial proteomes based on 2VO model rat brain tissue (**Figure** **3**A). Brain homogenate (10%, w/v) was prepared using a PotterElvehjem type glass Teflon homogenizer with a tight‐fitting Teflon pestle, and mitochondrial fractions were isolated and furtherly purified by sucrose density gradient centrifugation. Concomitantly, mitochondrial purity was assessed by western blotting (WB) against mitochondrial and cytoplasmic marker proteins, which were voltage‐dependent anion‐selective channel 1 (VDAC1) and F‐actin, respectively. WB experiment showed that the isolated mitochondria had high purity and little cytoplasmic interference, which was suitable for mitochondrial proteome characterization (Figure 3C). Furthermore, we used high‐resolution mass spectrometry (MS) for proteomics data acquisition and automated computational analysis in PEAKS software and detected 5449 proteins in total in sham‐operation, 2VO model, and SG treatment groups with criteria of FDR < 1% at peptide and protein levels. We then subjected quantified brain proteome data to MitoMiner 4.0,^[^ ^28^ ^]^ which is used MitoCarta 2.0 database and IMPI (Version Q2 2018) database to search for mitochondrial proteins and a substantial portion (1113 proteins) of brain proteome were recognized as belonging to mitochondrial origin (Table S1, Supporting Information). The Venn diagram (Figure 3B) illustrated the criteria for mitochondrial protein identification by these databases. To determine the up‐ or down‐regulation, the mean peak intensity of brain mitochondrial proteins between five independent samples in every group was measured, then their fold changes (FC>1.3) were plotted against ‐log *p*‐values (*p* < 0.05) in a volcano plot. Based on the frequency distribution, we identified 583 and 136 differentially expressed mitochondrial proteins (DEMPs, p‐value<0.05) between the model versus sham‐operation group and SG versus model group, of which there were 525 and 79 DEMPs with FC>1.3, respectively (Figure 3D,E).

We subsequently investigated how these DEMPs affected CCH pathology and SG effect in an unbiased manner. Proteinprotein interaction (PPI) enrichment analysis of all DEMPs was carried out with the following databases: STRING, BioGrid, OmniPath, InWeb_IM. The Molecular Complex Detection (MCODE) algorithm^[^ ^29^ ^]^ was applied to identify densely connected network components, and the three best‐scoring terms by p‐value have been retained as the functional description of the corresponding components (Figure 3F). Of that, MCODE_1 and MCODE_2, contained most of the DEMPs (55 and 21 proteins), were both related to OXPHOS and its upstream TCA cycle, accounting for 34.2% and 14.0% of the total, respectively (Figure 3G,H). It was proved that the CCH model and SG effect on mitochondria were mainly related to mitochondrial aerobic energy metabolism. Additionally, function annotation, performed by the DAVID clustering tool, indicated that the DEMPs of the model versus sham‐operation group and SG versus model group were consistent in the following biological processes: Electron transport, Respiratory chain, TCA cycle, Transport, Glycolysis, etc. (Figure 3I,J). By the way, changes in lipid‐related metabolism pathways were enriched in the model group compared with the control group, whereas SG was found to be primarily enriched in mitochondrial glucose aerobic metabolism pathways rather than lipid metabolism pathways. Specifically, there were 85 DEMPs both belong of the model versus sham‐operation group and SG versus model group, respectively (Table S2, Supporting Information). The PPI enrichment analysis showed that these DEMPs were closely related to mitochondrial aerobic respiration with pyruvate dehydrogenase (PDH) complex (PDC) component E2 (Dlat) as the core (Figure 3K). These DEMPs involved in mitochondrial aerobic respiration related metabolic pathways included mitochondrial respiratory chain complex I/IV assembly, electron/hydrogen ion transport, TCA cycle, glycolytic process, etc. (Figure 3L).

These results suggested that SG may enhance the upstream pathways of aerobic respiration, TCA cycle and glycolysis, with PDH as the core, to enhance hydrogen proton and electron supply, and alleviate mitochondrial damage in CCH brain tissue.

### 2.4 Scutellarin Regulated Mitochondrial Aerobic Metabolism and Morphology of Mitochondria during Mitochondrial OXPHOS Damage

**Figure 4:**
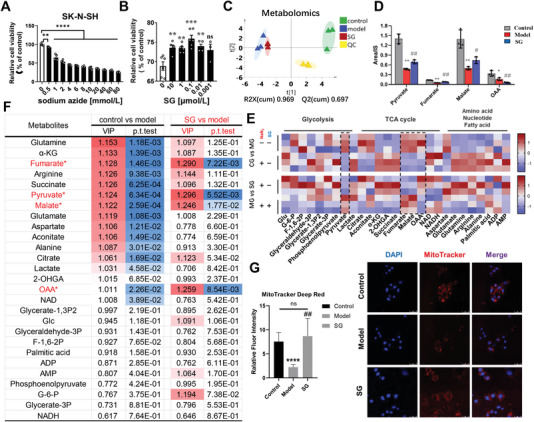
Metabolomics and morphology of mitochondria upon NaN_3_‐induced cell damage revealed that SG regulates pyruvate metabolism linked glycolysis and TCA cycle during mitochondrial OXPHOS damage. A) The dose‐dependent effect of NaN_3_‐induced mitochondria damage model. B) SG protective effect on NaN_3_‐induced mitochondrial damage. C) Principal component analysis (PCA) showed that the control group, model group and SG group were well distinguished. D) Potential biomarkers compared SG with model group. E) Application of OPLS‐DA model to screen the potential biomarkers with VIP value greater than 1, and student's *t*‐test value was < 0.05. F) Metabolomics analysis including 27 major metabolites involved in cell energy metabolism and related metabolic pathways. G) The morphology of mitochondria upon NaN_3_‐induced mitochondrial damage and SG treatment. Data are presented as mean ±SEM by paired two‐tailed Student's *t*‐test, ^*^ *p* < 0.05, ^**^ *p* < 0.01, ^***^ *p* < 0.001, ^****^ *p* < 0.0001, model versus control group, ^#^ *p* < 0.05, ^##^ *p* < 0.01, ^###^ *p* < 0.001, ^####^ *p* < 0.0001, SG versus model group.

The objective is to validate the findings of mitochondrial proteomics in the CCH model, demonstrating that SG regulates mitochondrial OXPHOS and its upstream pathways under CCH conditions. We conducted metabolomics studies based on the cell model of sodium azide (NaN_3_) injury, which can impair ATP production of mitochondrial OXPHOS.^[^ ^30^ ^]^ The human neuroblastoma SK‐N‐SH cell line was used to establish the NaN_3_‐induced mitochondria damage model, and the dose‐dependent effect of NaN_3_ on the cell viability of SK‐N‐SH cell line was summarized in **Figure** **4**A. Treatment of NaN_3_ from 0.5 to 80 mmol L^−1^ significantly inhibited cell viability of SK‐N‐SH cells, the optimal concentration of 1 mmol L^−1^ NaN_3_ was applied to establish the NaN_3_‐induced mitochondrial damage cell model, and 24 h exposure to 1 mmol L^−1^ NaN_3_ induced a 40% decline in cell viability. We investigated the impact of SG on cell viability in NaN_3_‐induced mitochondrial damage cells, and the findings demonstrated that pre‐incubation with 0.01, 0.1, 1, and 10 µmol L^−1^ SG for a duration of 4 h significantly enhanced the cell viability of the experimental model cells. This indicates a substantial protective effect against NaN_3_‐induced mitochondrial damage (Figure 4B). Moreover, we have evaluated the cytotoxicity of SG to SK‐N‐SH cells. The results showed that SG did not exhibit cytotoxicity at concentrations ranging from 0.1 to 30 µmol L^−1^ (Figure S2, Supporting Information).

Based on this, we first carried out a metabolomics study to quantitatively analyze a total of 27 major metabolites involved in cell energy metabolism and related metabolic pathways, including glycolysis, TCA cycle, amino acid, nucleic acid, and fatty acid metabolism. Principal component analysis (PCA) showed that the control group, model group, and SG group were well distinguished, and the vector distance of SG group was closer to the control group than that of model group (Figure 4C). Compared with the model group, 4 metabolites simultaneously satisfied the above conditions both in the control group and SG group, including fumarate, malate, and oxaloacetate (OAA) in TCA cycle, as well as pyruvate, a key link metabolite in glycolysis and TCA cycle (Figure 4D,E). Next, the quantitative analysis results of metabolites were fitted with orthogonal partial least squares discriminant analysis (OPLS‐DA) model and the potential biomarkers were screened with variable important in projection (VIP) value greater than 1, with student's *t*‐test value was < 0.05 (Figure 4F). The validation model and the permutation test of the OPLS‐DA model were shown in Figures S3 and S4 (Supporting Information). Additionally, the morphology of mitochondria upon NaN_3_‐induced mitochondrial damage was visualized using confocal microscopy by staining nuclear with DAPI and mitochondrial with MitoTracker Deep Red (with a final concentration of 0.5 µmol L^−1^). We observed that cells exposed to 24 h NaN_3_ exhibited a decrease in area of MitoTracker Deep Red staining compared to the untreated control cells, indicating a loss of mitochondrial membrane potential, which was in accordance with previous animal experiment. As shown in Figure 4G, SG exhibited a significant inhibition of depolarization induced by NaN_3_, as evidenced by an increase in MitoTracker Deep Red intensity compared to the model group. These findings suggest that SG may have a protective effect against mitochondrial injury caused by NaN_3_. Notably, there was no significant difference in MitoTracker Deep Red intensity between SG and control groups. In summary, metabolomics and morphology of mitochondria upon NaN_3_‐induced cell damage revealed that SG regulates pyruvate metabolism linked glycolysis and TCA cycle during mitochondrial OXPHOS damage, reducing mitochondrial damage and improving cell survival.

### 2.5 Real‐Time Cellular Bioenergetics Analysis to Verify the Effect of Scutellarin on Mitochondrial Aerobic Respiration

**Figure 5:**
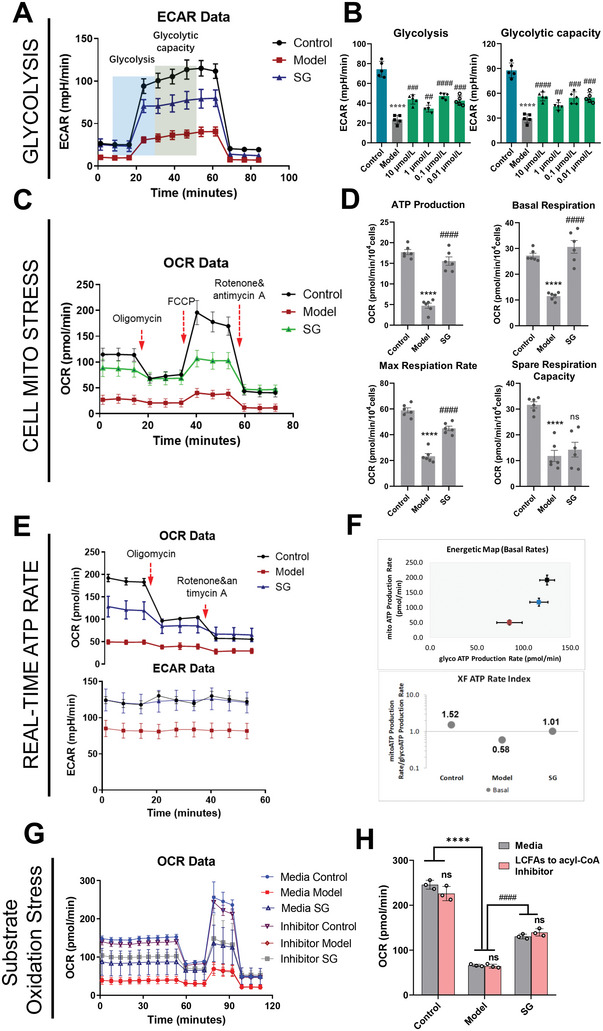
The evaluation of real‐time ECARs and OCRs in intact cells after NaN_3_‐induced mitochondria OXPHOS damage and SG treatment. A) The plot of glycolytic stress test. B) ECARs for the glycolysis and glycolysis capacities of control, model and SG treatment group. C) The plot of mitochondrial stress test. D) OCRs for basal respiration, ATP‐linked respiration, maximal respiration, and spare respiratory capacity of control, model, and SG treatment group. E) The plot of real‐time ATP rate test. F) SG could significantly reverse the mitoATP/glycoATP ratio compared with model group. G) The plot of substrate oxidation stress test. H) OCRs between medium group and LCFAs oxidation inhibitor group. Data are presented as mean ±SEM by paired two‐tailed Student's *t*‐test, ^*^ *p* < 0.05, ^**^ *p* < 0.01, ^***^ *p* < 0.001, ^****^ *p* < 0.0001, model versus control group, ^#^ *p* < 0.05, ^##^ *p* < 0.01, ^###^ *p* < 0.001, ^####^ *p* < 0.0001, SG versus model group.

To verify that SG regulates pyruvate metabolism linked glycolysis and TCA cycle and thus enhances mitochondrial glucose oxidation during mitochondria OXPHOS damage, we followed the evaluation of real‐time extracellular acidification rates (ECARs) and oxygen consumption rates (OCRs) in intact cells after NaN_3_ induced mitochondria OXPHOS damage and SG treatment. We first examined the changes of ECARs in the control group, model group and SG group with glucose as carbon source (**Figure** **5**A). SK‐N‐SH cells pretreated by starvation (without glucose and sodium pyruvate) were given saturated glucose, and then utilized the added glucose and decomposed it into pyruvate and lactate through the glycolysis pathway to produce ATP, NADH, H_2_O, and protons, resulting in a rapid increase of ECARs in the surrounding solution. Changes in ECARs during this process can be used to measure the basal glycolysis rate. We furtherly added oligomycin, an inhibitor of ATP synthase, which inhibits mitochondrial ATP production, to calculate the maximum glycolysis capacity of cells by increasing ECARs. The results of ECARs showed that both the basal glycolysis and glycolysis capacities of model group were significantly decreased compared with control group, and were significantly enhanced by SG treatment groups with a series of concentration (0.01, 0.1, 1, and 10 µmol L^−1^) (Figure 5B). Differences in ECARs promoted by SG treatment under our conditions may be related to pyruvate and lactate production, changes in TCA cycle flux (due to CO_2_ production), ATP turnover, or other acid‐generating catabolic processes. To evaluate the acidification source under our conditions, we measured the TCA cycle flux by monitoring the OCRs changes among the groups (Figure 5C). The results of cell mitochondrial stress test indicating that NaN_3_‐treated SK‐N‐SH cells maintained a reduced basal OCRs, and responded minimally to the introduction of oligomycin, FCCP, and a mix of rotenone and antimycin A, respectively, which indicated mitochondrial bioenergetic deficits. As quantitatively assessed, pre‐treatment of SG (1 µmol L^−1^) significantly up‐regulated OCRs for basal respiration, ATP‐linked respiration, and maximal respiration, respectively (Figure 5D). In addition, the results of real‐time ATP rate test, which could measure OCRs and ECARs simultaneously, revealed that SK‐N‐SH cells treated with NaN_3_ had lower total ATP production rates than those of the control group, and NaN_3_ significantly reduced both mitochondrial ATP production rate and glycolysis ATP production rate. On the contrary, SG (1 µmol L^−1^) was able to significantly up‐regulate both mitochondrial and glycolysis ATP production (Figure 5E). Moreover, it was noteworthy that SG could significantly reverse the mitoATP/glycoATP ratio compared with model group (0.58 up to 1.01), indicating that SG mainly enhanced cellular energy supply by regulating mitochondrial aerobic metabolism (Figure 5F). Long‐chain fatty acids (LCFAs) can enter the mitochondrial TCA cycle through metabolism to acetyl‐CoA. Are long‐chain fatty acids involved in the aerobic metabolism enhanced by SG treatment? Etomoxir (Eto) inhibits the oxidation of LCFAs by inhibiting carnitine palmityl transferase 1A (CPT1a). We measured the changes of OCRs between groups after the addition of Eto to evaluate the effects of LCFAs oxidation on mitochondrial aerobic respiration (Figure 5G). The results indicated that there was no significant change in OCRs between the medium group and inhibitor group, suggesting that the improvement of mitochondrial aerobic respiration by SG (1 µmol L^−1^) was not attributed to LCFA oxidation (Figure 5H). Overall, these results confirmed the results of that SG enhanced mitochondrial aerobic respiration by regulating glycolysis and TCA cycle linked by pyruvate metabolism, and ultimately ameliorates NaN_3_‐induced mitochondrial respiratory dysfunction.

### 2.6 Exploration of [^13^C_6_]‐Glucose Labeled Steady‐State Condition of Energy Metabolism Pathways in SK‐N‐SH Cells

**Figure 6:**
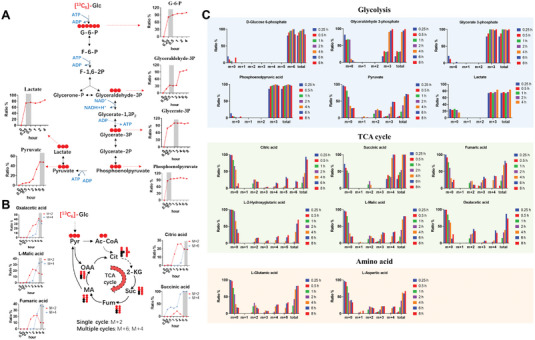
Exploration of [^13^C_6_]‐glucose labeled steady‐state condition of energy metabolism pathways. A) The kinetics of ^13^C labeled key metabolites in glycolysis. B) The kinetics of ^13^C labeled key metabolites in TCA cycle. C) The isotopologue distribution of the metabolites at different time points. m, monoisotopic metabolite; m+n, isotopologue distribution of the metabolites.

Based on the results of real‐time cellular bioenergetics analysis, we then conduct [^13^C_6_]‐glucose metabolic flux analysis (^13^C‐MFA) to explore the molecular regulation mechanism of SG. To monitor glycolysis and TCA cycle in the setting of OXPHOS damage and SG treatment, we incubated exponentially growing SK‐N‐SH cells with uniformly labeled [^13^C_6_]‐glucose, harvested metabolites over time (0, 0.25, 0.5, 1, 2, 4, 6, and 8 h), subjected the extracts to analyze the metabolites by liquid chromatography coupled to high‐resolution mass spectrometry (LC‐HRMS). The kinetics of [^13^C_6_]‐G6P, [^13^C_3_]‐glyceraldehyde‐3P, [^13^C_3_]‐glycerate‐3P, [^13^C_3_]‐phosphoenolpyruvate, [^13^C_3_]‐pyruvate, [^13^C_3_]‐lactate in glycolysis and [^13^C_2_]‐Cit/[^13^C_6_]‐Cit, [^13^C_2_]‐Suc/[^13^C_4_]‐Suc, [^13^C_2_]‐Fum/[^13^C_4_]‐Fum, [^13^C_2_]‐MA/[^13^C_4_]‐MA, [^13^C_2_]‐OAA/[^13^C_4_]‐OAA in TCA cycle were measured through the identification and quantitative of the isotopologues, respectively. The isotopologue distribution of the metabolites at different time points were shown in **Figure** **6**C. The results showed that most metabolites in glycolysis reached labeled homeostasis at 0.5 h, while pyruvate, which connects glycolysis with the TCA cycle and associated with amino acid metabolism and lipid metabolism, reached labeled homeostasis at 6 h (Figure 6A). In the TCA cycle, the steady‐state time of metabolites labeled with single cycle ([^13^C_2_]‐labeled) and multiple cycles ([^13^C_4_]‐labeled or [^13^C_6_]‐labeled) was investigated. The results showed that both single and multiple cycle could reach labeled homeostasis at 6 h (Figure 6B). Finally, we selected two observation time points of 0.5 and 6 h to evaluate the changes of upstream metabolic flux of OXPHOS in NaN_3_‐induced mitochondrial damage model group and SG treatment group, respectively.

### 2.7 ^13^C‐MFA Revealed SG Strengthen Pyruvate to Acetyl‐CoA Linked Mitochondrial Glucose Oxidation during Mitochondrial OXPHOS Damage

**Figure 7:**
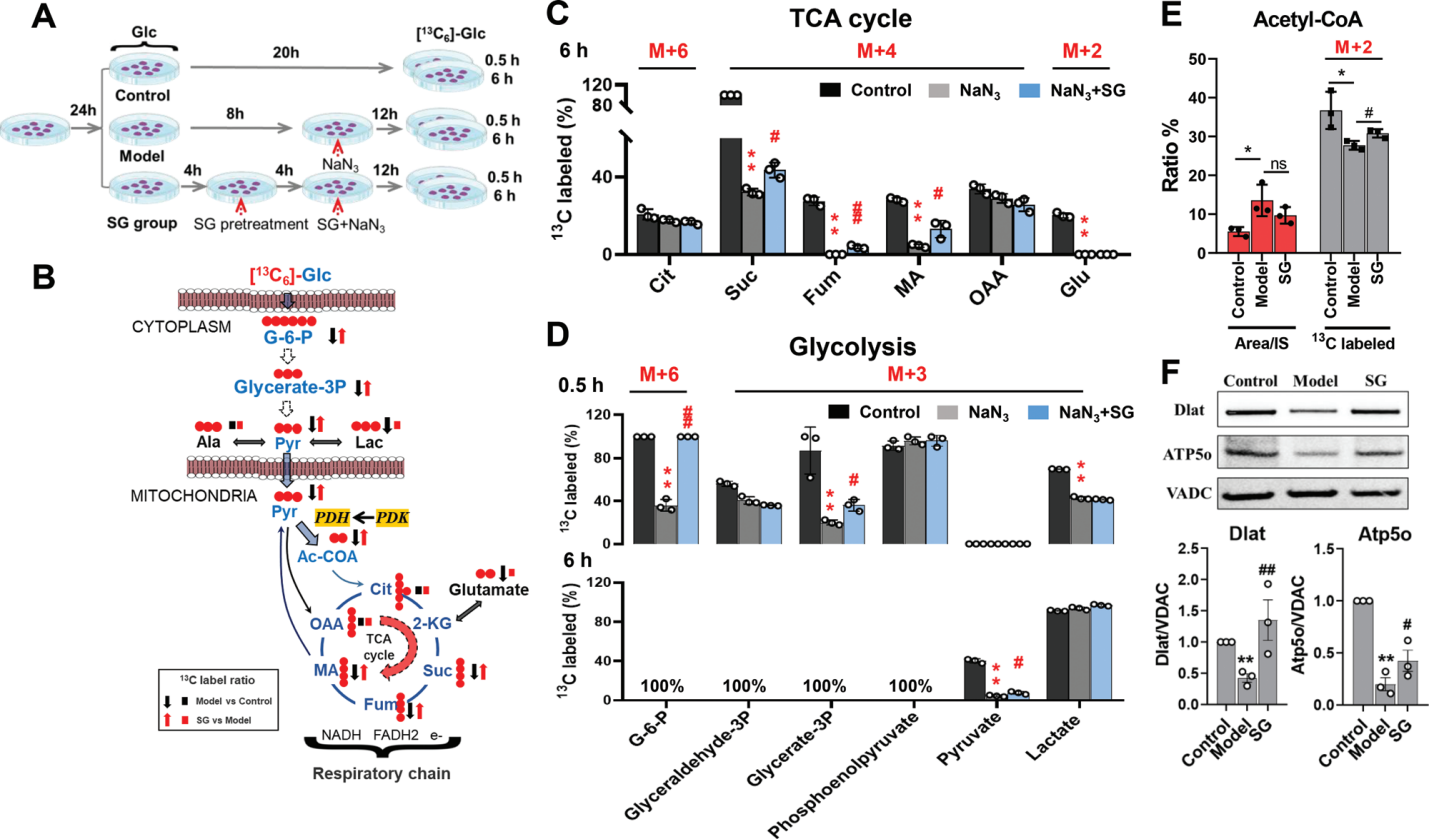
^13^C MFA and western blotting revealed that SG could regulate pyruvate metabolism linked glucose‐pyruvate‐TCA cycle axis. A) The incubation process of ^13^C‐MFA experiment. B) SG could regulate PDK‐PDC axis linked glucose‐pyruvate‐TCA cycle axis under the condition of impaired OXPHOS. C) The abundance ratio of ^13^C metabolites in TCA cycle (6 h). D) The abundance ratio of ^13^C metabolites in glycolysis (0.5 h and 6 h). E) SG significantly enhanced the flux of [^13^C_3_]‐pyruvate metabolism to [^13^C_2_]‐acetyl‐CoA compared to the model group. F) The expression of *Dlat* and *Atp5o* of control, model, and SG treatment group. Data are presented as mean ±SEM by paired two‐tailed Student's *t*‐test, ^*^ *p* < 0.05, ^**^ *p* < 0.01, ^***^ *p* < 0.001, ^****^ *p* < 0.0001, model versus control group, ^#^ *p* < 0.05, ^##^ *p* < 0.01, ^###^ *p* < 0.001, ^####^ *p* < 0.0001, SG versus model group.

Our metabolomics studies revealed that SG could regulates the upstream metabolic pathway of mitochondrial OXPHOS and electron transport chain (ETC). As ETC activity is linked to the supply of carbon substrates, to provide further evidence for the metabolic regulatory mechanism of SG, we performed ^13^C‐MFA using LC‐HRMS technique to study the alteration of metabolism in amino acid, glycolysis, TCA cycle, etc. The ^13^C‐MFA experimental process is shown in **Figure** **7**A. Universally labeled [^13^C_6_]‐glucose was employed as a tracer to track the metabolites in these pathways. The medium of NaN_3_ induced mitochondrial damage model and SG treatment group (1 µmol L^−1^) was replaced by [^13^C_6_]‐glucose medium after continuous culture for 12 h, and samples were collected at 0.5 and 6 h for pre‐treatment and furtherly LC‐HRMS analysis, respectively. We analyzed the metabolic flux of upstream metabolic pathways of OXPHOS, and the results showed that compared with the control group, the abundance ratios of [^13^C_6_]‐G6P (0.5 h), [^13^C_3_]‐glycerate‐3P (0.5 h), [^13^C_3_]‐lactate (0.5 h) and [^13^C_3_]‐pyruvate (6 h) of glycolysis pathway in model group was significantly decreased. However, in the SG group, the abundance ratio of [^13^C_6_]‐G6P (0.5 h), [^13^C_3_]‐glycerate‐3P (0.5 h), [^13^C_3_]‐lactate (0.5 h), and [^13^C_3_]‐pyruvate (6 h) could be significantly reversed. Notably, there was no significant difference in the abundance ratio of [^13^C_3_]‐lactate (0.5 h) between model and the SG group, indicating that the enhanced glycolysis did not increase energy supply through the lactate metabolic pathway, but mainly flowed to pyruvate metabolism (Figure 7D). We furtherly examined the flux of key metabolites in the TCA cycle and found that the abundance ratio of [^13^C_4_]‐Suc, [^13^C_4_]‐Fum, [^13^C_4_]‐MA and [^13^C_2_]‐Glu were significantly decreased in the model group compared with the control group, while that of [^13^C_4_]‐Suc, [^13^C_4_]‐Fum and [^13^C_4_]‐MA were significantly reversed in the SG group. However, the abundance ratio of [^13^C_2_]‐Glu was consistent between model and the SG group, suggesting that SG could not regulate the glutamine‐glutamate‐2‐KG metabolic pathway to enhance the TCA cycle pathway. Simultaneously, pyruvate‐alanine metabolic pathway was monitored and no significant difference was observed in [^13^C_3_]‐alanine abundance ratio among the groups (Figure 7C). Most importantly, we found that SG significantly enhanced the flux of [^13^C_3_]‐pyruvate metabolism to [^13^C_2_]‐acetyl‐CoA compared to the model group, thereby enhancing mitochondrial glucose source aerobic respiration (Figure 7E). Furthermore, we investigated the key protein expression of pyruvate dehydrogenase E2 *(Dlat)* and ATP synthase subunit O *(ATP5o)* by western blotting which catalyzes pyruvate to acetyl‐CoA and then to the TCA cycle and produces ATP from ADP generated by ETC of the respiratory chain, respectively. These proteins both were down‐regulated in the model group compared with the control group and could be significantly reversed in SG treatment group (Figure 7F). The original diagrams of three biological repeats of the western blotting experiment of *Dlat* and *Atp5o* were shown in Figure S5 (Supporting Information). To sum up, we believe that SG could regulate PDK‐PDC axis linked glucose‐pyruvate‐TCA cycle axis under the condition of impaired OXPHOS, and increasing the aerobic metabolism of electronic respiratory chain in the oxidative phosphorylation process (Figure 7B).

### 2.8 SG Promoted Mitochondrial Aerobic Respiration and Mitochondrial Membrane Potential by Selectively Inhibiting PDK2 as the Target

**Figure 8:**
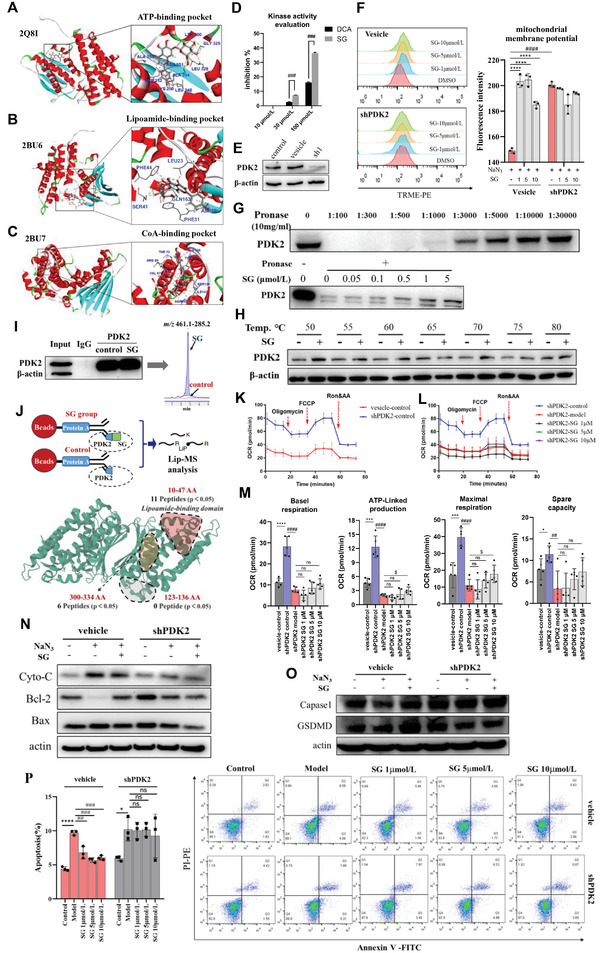
SG promoted mitochondrial aerobic respiration and regulated mitochondrion‐dependent apoptosis via inhibiting PDK2 as the target. A) Docking analysis of SG spatially bond around ATP‐binding domain of PDK2. B) Docking analysis of SG spatially bond around lipoamide‐binding domain of PDK2. C) Docking analysis of SG spatially bond around CoA‐binding domain of PDK2. D) The percent inhibition ratio of SG against PDK2 compared with DCA. E) Western blot of SK‐N‐SH cell line with stable knockdown of PDK2 (shPDK2). F) Mitochondrial membrane potential evaluation of vehicle SK‐N‐SH cell and shPDK2 SK‐N‐SH cell during mitochondrial OXPHOS damage. G) The western blot of the DARTS experiment. H) The western blot of the CETSA experiment. I) The results of the co‐IP‐MS experiment. J) The results of the Lip‐MS experiment. K) Cell mitochondrial stress test of vehicle SK‐N‐SH cell and shPDK2 SK‐N‐SH cell. L) Cell mitochondrial stress test of shPDK2 SK‐N‐SH cell during mitochondrial OXPHOS damage and SG administration. M) OCRs for basal respiration, ATP‐linked respiration, maximal respiration, and spare respiratory capacity of shPDK2 SK‐N‐SH cell during mitochondrial OXPHOS damage and SG administration. N) the western blot of mitochondria‐dependent apoptosis‐related proteins. O) The western blot of pyroptosis‐related proteins. P) Flow cytometry to compare apoptosis ratio of vehicle SK‐N‐SH cells and shPDK2 SK‐N‐SH cells under mitochondrial damage and SG administration. Data are presented as mean ±SEM by paired two‐tailed Student's *t*‐test, ^*^ *p* < 0.05, ^**^ *p* < 0.01, ^***^ *p* < 0.001, ^****^ *p* < 0.0001, model versus control group, ^#^ *p* < 0.05, ^##^ *p* < 0.01, ^###^ *p* < 0.001, ^####^ *p* < 0.0001, SG versus model group.

To verify the effect of SG on pyruvate metabolism regulated by PDK‐PDC axis to enhance mitochondrial aerobic metabolism, we conduct kinase activity evaluation by measuring the non‐phosphorylated and phosphorylated substrate in the kinase reaction. The percent inhibition ratio of SG, against PDK2 and PDK4, was tested with dichloroacetic acid (DCA) as the positive control.^[^ ^31^ ^]^ The results showed that SG had significantly higher inhibitory activity against PDK2 than DCA at multiple concentrations (**Figure** **8**D). Then, docking analysis showed that SG could spatially bond around ATP‐binding domain, lipoamide‐binding domain, and CoA‐binding domain of PDK2 (Figure 8A–C), with the LibDock score or CDOCKER_Interation_Energy higher than all the ligands, respectively (Figure S6, Supporting Information). Meanwhile, the specific amino acids that SG bind to each domain through hydrogen bonding were shown in Figure S7 (Supporting Information). The Drug Affinity Responsive Target Stability (DARTS) experiment was performed for the evaluation of the interaction between SG and PDK2. As shown in the Figure 8G, when the ratio of protease: protein was 1:3000, there was still a certain amount of protein remaining and it was significantly different from the control group, so this ratio was selected for subsequent experiments. After incubation for 2 h at room temperature with different concentrations of SG (0.05–5 µmol L^−1^), compared with DMSO in the control group, the PDK2 protein was higher than that of DMSO in a concentration‐dependent manner, which indicated that the PDK2 protein stability is increased due to SG binding, and further proved the interaction between SG and PDK2 protein. The original western blot plots of the DARTS experiments were shown in Figure S8 (Supporting Information). The CETSA experiment confirmed the interaction between SG and PDK2 protein at molecular level, that the PDK2 protein in SK‐N‐SH cells degraded with the increase of temperature, but the addition of SG reduced the degradation of PDK2 protein (Figure 8H). The original western blot plots of the CETSA experiments were shown in Figure S9 (Supporting Information). SG incubation group showed significantly higher stability of PDK2 protein compared with control group in the range of 50 to 80 °C. For function verification, we obtained the SK‐N‐SH cell line with stable knockdown of PDK2 (shPDK2) (Figure 8E; Figure S10, Supporting Information). Based on this, we found that SG could play a protective role against mitochondrial membrane potential damage induced by NaN_3_ with PDK2 as the target (Figure 8F). Furtherly, we conduct the co‐immunoprecipitation (IP) experiment to study small molecular‐protein interactions. As shown in Figure 8I and Figure S11 (Supporting Information), the protein bands of the input group indicated that the cell lysate contained PDK2 protein, and the negative control IgG group had no target bands, which ruled out the possibility of non‐specific binding of the protein to the antibody. The experimental group incubated with SG had bands of PDK2, indicating that PDK2 protein was successfully precipitated by immunoprecipitation experiment with PDK2 antibody. Next, the immunoprecipitated beads were washed repeatedly four times through PBS solution to remove non‐specific residues attached to the bead surface. Finally, we performed LC‐MS/MS to analyze the SG binding to the PDK2. The results clearly showed that the interaction between SG and PDK could be detected by co‐immunoprecipitation‐MS. Small molecular‐protein interactions typically result in local or in global alterations of protein structures. Detecting ligand‐induced structural alterations on a proteome‐wide scale could provide a universal readout of the interactions.^[^ ^32^ ^]^ In this study, the immunoprecipitated beads extracted under native lysis conditions were treated or not with SG, with preincubated for 1 h at 1 mmol L^−1^. A limited proteolysis step is performed with proteinase K (PK) under native conditions, followed by complete digestion with trypsin under denaturing conditions to generate MS‐measurable peptides. In total, 371 peptides of PDK2 were identified based on Lip‐MS. Statistical analysis was performed to find differentially expressed peptides between the control and SG treated groups. There were 11, 0, and 6 differential peptides were identified in lipoamide‐binding domain, CoA‐binding domain and ATP‐binding domain, respectively (Table S3, Supporting Information). These experimental results further proved the binding effect of SG and PDK2. Moreover, the lipoamide‐binding domain might be the main binding region of SG binding to PDK2 (Figure 8J). In addition, cell mitochondrial stress test indicated that shPDK2 SK‐N‐SH cells maintained higher mitochondrial aerobic respiration indices than vehicle SK‐N‐SH cells, and SG could up‐regulate OCRs for ATP‐linked respiration and maximal respiration only at high concentration (10 µmol L^−1^) (Figure 8K–M). Compared to the control group showed in Figure 5C,D, we concluded that SG promote mitochondrial aerobic respiration via inhibiting PDK2 as the target.

### 2.9 SG Regulated Mitochondrion‐Dependent Apoptosis by Targeting PDK2

To study the process of cell death caused by mitochondrial damage, we conduct proteomics to explore the effect of SG on mitochondrial damaged cells. 4052 proteins, including 509 mitochondrial proteins were identified by MitoMiner 4.0 database. Among them, there were 65 mitochondrial proteins with FC greater than 1.5 and 28 mitochondrial proteins with FC greater than 2.0 (Figure S12, Supporting Information). The PPI enrichment analysis of the above proteins were focused on OXPHOS and TCA cycle, including pyruvate kinase (PKM) and pyruvate dehydrogenase kinase 2 (PDK2). Meanwhile, we uncovered these proteins were associated with a variety of apoptotic processes in biological process analysis (Figure S12, Supporting Information). The critical role of neuronal apoptosis in ischemia‐induced brain injury has been shown in both human and animal studies. In addition to being known as cellular powerhouses, mitochondria appear to play a key role in the cell death machinery because of their associations with a long list of apoptosis‐related proteins.^[^ ^33^ ^]^ In this study, we used flow cytometry to compare apoptosis ratio of vehicle SK‐N‐SH cells and shPDK2 SK‐N‐SH cells under NaN_3_‐induced mitochondrial damage and SG administration. As quantitative analysis results shown, SG significantly reduced vehicle SK‐N‐SH cells apoptosis caused by mitochondrial damage at different concentrations, but this phenomenon was not observed in shPDK2 SK‐N‐SH cells (Figure 8P). Cytochrome c is primarily known for its function in the mitochondria as a key participant in the life‐supporting function of ATP synthesis. However, when a cell receives an apoptotic stimulus, cytochrome c is released into the cytosol and triggers programmed cell death through apoptosis. The release of cytochrome c to the cytoplasm and cytochrome‐c‐mediated apoptosis are controlled by multiple layers of regulation, the most prominent players being members of the B‐cell lymphoma protein‐2 (Bcl‐2) family.^[^ ^34^ ^]^ The western blot results demonstrated a significant increase in cytochrome c expression and a significant decrease in Bcl‐2 protein expression in vehicle‐treated SK‐N‐SH cells with mitochondrial damage. However, SG treatment significantly increased the expression of Bcl‐2 protein during mitochondrial damage. Correspondingly, SG showed no such effect in shPDK2 SK‐N‐SH cells (Figure 8N; Figure S13, Supporting Information). Since mitochondrial dysfunction will generate excessive ROS and induce pyroptosis,^[^ ^35^ ^]^ we examine the regulation of SG on pyroptosis. The classical pyroptosis pathway is mainly mediated by caspase‐1. Studies showed that activated caspase‐1 cleaved at the middle linker of the GSDMD protein, releasing a 31 kDa N‐terminal fragment of GSDMD (GSDMD‐N) that initiates pyroptosis. Therefore, we investigated the effects of SG on caspase‐1 and GSDMD, the key proteins in the classical pyrodeath pathway. The western blot results demonstrated no significant different among the groups of caspase‐1 and GSDMD in vehicle‐treated SK‐N‐SH cell and shPDK2 SK‐N‐SH cell (Figure 8O; Figure S14, Supporting Information). This suggested that SG exert neuroprotective effect against mitochondrial damage‐induced cell death primarily by regulating the mitochondria‐dependent apoptotic pathway. In summary, these results suggested that SG regulates mitochondrion‐dependent apoptosis by targeting PDK2.

## 3 Discussion

Considerable interest has grown in recent years concerning the role of mitochondria on the development and progression of neurocognitive disorders, including VaD and AD.^[^ ^36^ ^]^ Mitochondria dysfunction related pathophysiological signaling may upregulate several pathological mechanisms, including glucose dyshomeostasis, inflammation, oxidative stress, and neurotoxicity.^[^ ^37^, ^38^, ^39^ ^]^ These trigger memory and cognitive dysfunction—before frank AD or VaD. Simultaneously, the high energy demand of the brain renders it sensitive to changes in energy fuel supply and mitochondrial function. Deficits in glucose availability and mitochondrial function are well‐known hallmarks of brain aging and are particularly accentuated in neurodegenerative disorders such as AD during chronic hypoperfusion.^[^ ^40^ ^]^ In this study, we found that SG could improve learning and memory ability in model 2VO rats by pathological staining of hippocampal CA1 and corpus striatum region and behavioral evaluation, respectively. Considering the relationship between chronic hypoperfusion and bioenergetic deficits and oxidative stress injury, we furtherly investigated the effects of SG on REDOX related biochemical indicators in 2VO model rats, and the results showed that SG could regulate oxidative stress related peroxidases. Given that mitochondria play a key role in both bioenergetic deficits and oxidative stress, we then examined several of mitochondrial parameters. The results showed that SG could significantly reduce the level of ROS, and increase Na^+^‐K^+^ ATPase and Ca^2+^‐Mg^2+^ ATPase activity in mitochondria of brain tissue and alleviate oxidative stress damage. After cerebral hypoperfusion injury, the structure of brain mitochondria was damaged, the membrane potential level was reduced, and the continuous opening of mPTP is induced, which leads to mitochondrial membrane swelling, and leads to mitochondrial calcium overload. For this situation, SG could repair mitochondrial membrane potential, reduce mitochondrial swelling and the calcium overload, and finally maintain mitochondrial homeostasis. Simultaneously, the activity of mitochondrial respiratory chain complex I, III, IV, and V were restored by SG treatment, accounting for explaining the improvement of OXPHOS function and mitochondrial aerobic respiration.

The above results indicated that SG could alleviate bioenergetic deficit and cognitive impairment caused by cerebral hypoperfusion by regulating mitochondrial status. Therefore, we further extracted mitochondria from 2VO rat brain tissue and carried out label‐free quantitative proteomics. Both function annotation and PPI enrichment analysis displayed the DEMPs were mainly associated with OXPHOS driven by the ETC and the upstream glycolysis and TCA cycle pathway, respectively. It means that SG may enhance the upstream pathways of aerobic respiration to enhance hydrogen proton and electron supply, alleviated the overexpression of OXPHOS related proteins, ameliorate mitochondrial damage and improve aerobic respiration.

On this assumption, we conducted metabolomics to verify the regulatory effect of SG on bioenergetics deficit during OXPHOS injury. The results showed that SG significantly increased the abundance of fumarate and malate in TCA cycle, and pyruvate, the key metabolite linking glycolysis to the TCA cycle. We followed the evaluation of real‐time ECARs and OCRs in intact cells. The results concluded that SG treatment could alleviate the glucose hypometabolism by enhancing both the glycolysis and OXPHOS. It is worth noting that SG could significantly reverse the mitoATP/glycoATP ratio compared with model group, proved that SG mainly enhanced cellular energy supply by regulating mitochondrial aerobic respiration. The ^13^C‐MFA results showed the abundance ratios of [^13^C_6_]‐G6P and [^13^C_3_]‐pyruvate in glycolysis could be significantly reversed by SG treatment compared with model group, and there was no significant difference in the abundance ratio of [^13^C_3_]‐lactate between model and SG group, indicating that the enhanced glycolysis did not increase energy supply through the lactate metabolic pathway, but mainly flowed to pyruvate metabolism. Most importantly, we found that SG significantly enhanced the flux of [^13^C_3_]‐pyruvate to [^13^C_2_]‐acetyl‐CoA compared to the model group, thereby enhancing mitochondrial glucose source aerobic respiration. In the TCA cycle, [^13^C_4_]‐Suc, [^13^C_4_]‐Fum, and [^13^C_4_]‐MA were significantly reversed by the SG group, and the abundance ratio of [^13^C_2_]‐Glu had no significant difference between model and SG group, suggesting that the glutamine‐glutamate‐2‐KG metabolic pathway was not responsible for the enhancement of TCA cycle. On the other hand, the [^13^C_3_]‐alanine abundance ratio among the group had no significant difference, indicating that the pyruvate‐alanine metabolism in the cytoplasm and mitochondria was not enhanced. To sum up, we believe that the flux of [^13^C_3_]‐pyruvate to [^13^C_2_]‐acetyl‐CoA regulating by PDK‐PDC axis is the most critical point during SG against mitochondrial OXPHOS damage.

The mitochondrial pyruvate dehydrogenase (PDH) complex (PDC) irreversibly decarboxylates pyruvate to acetyl‐CoA, thereby linking glycolysis to the TCA cycle and defining a critical step in cellular bioenergetics. Inhibition of PDC activity by pyruvate dehydrogenase kinase (PDK)‐mediated phosphorylation has been associated with the pathobiology of many disorders of metabolic integration.^[^ ^41^ ^]^ The original xenobiotic developed for clinical use as a PDK inhibitor is DCA, which was discovered to modulate glucose and fat metabolism before its direct effect on the PDC/PDK axis was known. DCA is most active against the ubiquitous PDK2 isoform (Ki≈0.2 mm), approximately equipotent against PDK1 and PDK4.^[^ ^42^ ^]^ To verify the effect of SG on pyruvate metabolism regulated by PDK‐PDC axis to enhance mitochondrial aerobic metabolism, we conduct kinase activity evaluation by comparing with DCA as the positive control. The results showed that SG had significantly higher inhibitory activity against PDK2 than DCA at multiple concentrations. Based on the results of molecular docking, co‐immunoprecipitation (co‐IP), Lip‐MS, DARTS and CETSA experiments, we have essentially concluded that SG interacts with PDK2 protein and probably inhibits its activity by binding to the lipoamide‐binding pocket of PDK2. Targeting of the lipoamide‐binding pocket is a new strategy for developing PDK inhibitors with an optimal inhibition of kinase activity. The lipoamide binding site is located at the N‐terminus of PDKs. Under normal conditions, PDKs bind to the E2 subunit of PDC through the lipoamide binding site, which swings around the E2 domain and contacts E1α to produce phosphorylation. The expression of the PDC E2 subunit (Dlat) under mitochondrial injury could be concurrently enhanced by SG. Collectively, SG exerts neuroprotective effects by enhancing mitochondrial aerobic metabolism, increasing mitochondrial membrane potential, and further reducing mitochondria‐dependent apoptotic processes by regulating the PDK‐PDC axis. For function verification, we obtained the shPDK2 SK‐N‐SH cell line and found that SG could play a protective role against mitochondrial membrane potential damage and mitochondrial aerobic respiratory damage induced by NaN_3_ with PDK2 as the target.

Furtherly, we conduct proteomics to explore the process of cell death caused by mitochondrial damage the effect of SG. There were 65 mitochondrial proteins with FC greater than 1.5 and 28 mitochondrial proteins with FC greater than 2.0. The PPI enrichment analysis of the above proteins were focused on OXPHOS and TCA cycle, including pyruvate kinase (PKM) and pyruvate dehydrogenase kinase 2 (PDK2). Meanwhile, we uncovered these proteins were associated with a variety of apoptotic processes in biological process analysis. Accumulating evidence suggests that a group of proteins of the B‐cell lymphoma (Bcl‐2) family are profoundly involved in the regulation of neuronal death in cerebral ischemia. The Bcl‐2 protein family is a major regulator of outer mitochondrial membrane permeability and plays critical roles in the intrinsic apoptotic pathway. Cytochrome c is released into the cytosol and triggers programmed cell death through apoptosis. The release of cytochrome c and cytochrome‐c‐mediated apoptosis are controlled by the Bcl‐2 family. In our study, we found that SG significantly reduced vehicle SK‐N‐SH cells apoptosis caused by mitochondrial damage at different concentrations, but this phenomenon was not observed in shPDK2 SK‐N‐SH cells. Simultaneously, the western blot confirmed the effects of mitochondrial damage and SG administration on Bcl‐2 protein and cytochrome c and other apoptosis‐related proteins, suggesting that SG regulated mitochondrion‐dependent apoptosis by targeting PDK2.

**Figure 9:**
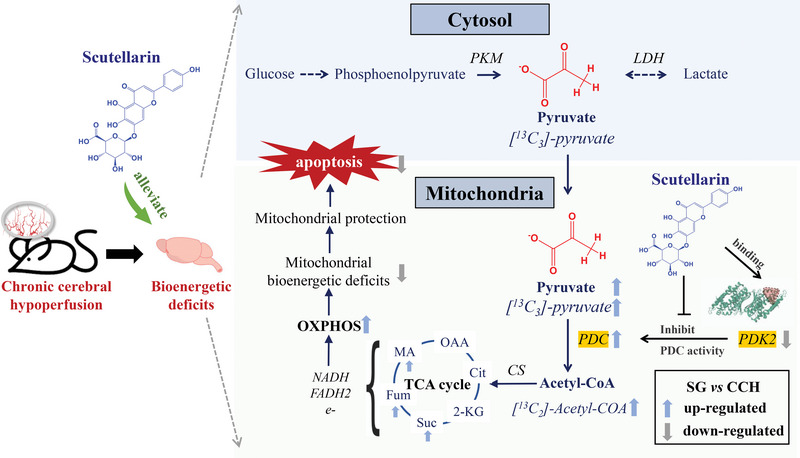
SG regulates PDK‐PDC axis and mitochondrial glucose oxidation.

In summary, we explored novel therapeutic approaches: the PDK‐PDC axis, which is targeting metabolic flexibility, for neurological injury and cognitive impairment caused by cerebral hypoperfusion, and discovered that SG exert mitochondrial protection and anti‐apoptotic activity by selectively targeting PDK2 (**Figure** **9**).

## 4 Conclusion

The present study provides evidence that enhancing mitochondrial dysfunction may represent a potential mechanism by which SG exerts its neuroprotective effects on CCH, with PDK2 serving as a promising pharmacological target. SG promoted mitochondrial aerobic respiration and mitochondrial membrane potential by inhibiting PDK2 and furtherly regulated mitochondrion‐dependent apoptosis. In general, we uncovered the PDK‐PDC axis for neurological injury and cognitive impairment treatment, and SG act on mitochondrial protection and anti‐apoptosis via the PDK‐PDC axis and mitochondrial glucose oxidation. This study provides a template for neurological diseases therapy by small molecules that target metabolic flexibility.

## 5 Experimental Section

### Materials

Scutellarin was purchased from Baoji Herbest Bio‐Tech Co. Ltd (Shaanxi, China), the purities of these reference standards were > 98% based on HPLC analysis. Nimodipine was purchased from Tianjin central pharmaceutical Co., Ltd. (Tianjin, China). Isotope labeled internal standard and [^13^C_6_]‐glucose tracer were purchased from Cambridge Isotope (Woburn, USA). LC‐MS‐grade acetonitrile and methanol and HPLC‐grade formic acid and ammonium bicarbonate were purchased from Fisher Scientific (New Jersey, USA). Ultrapure water was prepared using a Milli‐Q purification system (Massachusetts, USA). The mitochondrial extraction kit was purchased from Solarbio Science & Technology Corporation (Beijing, China). Assay kits of JC‐1 mitochondrial membrane potential, mitochondrial swelling, mitochondrial ROS, mitochondrial calcium, Na^+^‐K^+^ ATPase, and Ca^2+^‐Mg^2+^ ATPase, electron transport chain complex I, II, III, IV, and V were purchased from Genmed Scientifics Inc., (Shanghai, China).

### Animal Experiments and Drug Administration

All animal care and experimental procedures were reviewed and approved by the Institutional Animal Care and Use Committee of the Chinese Academy of Medical Sciences & Peking Union Medical College (approval No. 00003405). Animal studies were reported in compliance with the ARRIVE guidelines. Randomization was used to assign samples to the experimental groups for all in vivo studies. Data collection and evaluation of all in vivo and in vitro experiments were performed in a blind manner.

Male Sprague–Dawley rats were randomly assigned to receive permanent bilateral occlusion of common carotid arteries induction (CCH model) or sham operation. In the animal experiment, the sham operation group (*n* = 10) served as a control and the CCH modeled rats were randomly divided into the model group (*n* = 10) and scutellarin group (*n* = 10, 100 mg kg^−1^). Nimodipine, a well validated drug that was effective in the CCH pharmacological model, was used as a positive control to evaluate the efficacy of pharmacotherapies in two independent animal experiments (*n* = 6). The doses used in vivo were relevant for clinical translation. The model of chronic cerebral hypoperfusion and details of SG administration were shown in Supporting Information.

### Mitochondrial Samples Collection, Preparation, and Function Evaluation

The isolation of crude mitochondria was carried out according to the manufacturer's instructions. Briefly, brain or cell homogenate (10% w/v) was prepared using a Potter–Elvehjem type glass Teflon homogenizer with a tight‐fitting Teflon pestle (20 strokes on ice for brain samples and 30 strokes on ice for cell samples) and centrifuged at 800 g for 10 min to sediment nuclei and unbroken cell debris. The supernatant was centrifuged at 10 000 g for 10 min to sediment mitochondria. The crude mitochondrial pellet thus obtained was washed once by suspending in the isolation medium at 13 000 g for 10 min.

Mitochondrial fractions isolated after differential centrifugation were purified further by sucrose density gradient centrifugation. In Brief, mitochondrial fractions were prepared in SEM buffer (250 mmol L^−1^ sucrose, 1 mmol L^−1^ EDTA, 10 mmol L^−1^ MOPS‐KOH, pH 7.2) and then loaded onto a three‐step sucrose gradient [1.5 mL 60%, 4 mL 32%, 1.5 mL 23%, and 1.5 mL 15% sucrose in EM buffer (1 mmol L^−1^ EDTA, 10 mmol L^−1^ MOPS‐KOH, pH 7.2)] to obtain highly pure mitochondria. The samples were centrifuged for 1 h at 1 34 000 g and highly pure mitochondria were recovered from 32% to 23% sucrose interface. Mitochondrial purity was assessed by western blotting against various cellular marker proteins.

Functional evaluation of brain mitochondria was achieved by various parameters according to the manufacturer's instructions, such as electron transfer activity, mitochondrial ATPase activity, mitochondrial membrane potential, mitochondrial swelling, mitochondrial ROS, mitochondrial calcium.

### Mitochondrial Proteome

Proteomics analysis was performed on the highly pure mitochondria samples of sham operation group (*n* = 5), CCH model group (*n* = 5) and SG administration group (*n* = 5), especially. The samples from each group were prepared for LC‐MS/MS analysis using EasyPep Mini MS Sample Prep Kit (Thermo Scientific, Waltham, MA, USA). Briefly, mitochondria samples were transferred into a new tube and adjusted to 100 µL general lysis buffer. Afterward, incubate sample at 95 °C using a heat block for 10 min to reduce and alkylate the protein samples, 10% Trypsin/Lys‐C mix was added to digest proteins at 37 °C for 2 h. Digestion was stopped by acidification and desalted using a peptide clean‐up column.

Protein (100 µg) from each sample was dissolved with 80 µL of 0.1% FA in 2% acetonitrile and analyzed on UHPLC‐trapped ion mobility spectrometry coupled to tandem mass spectrometry (UHPLC‐TIMS‐TOF‐MS) system (Bruker Technologies). The peptides (3 µL injection volume) were separated using a C_18_ reverse‐phase capillary column (75 µm × 25 cm, Thermo Scientific, PepMap). The chromatographic separation was performed with a continuous acetonitrile gradient using 0.1% formic acid (A) and acetonitrile containing 0.1% formic acid (B) as mobile phases. The following program was run at 0.3 µL min^−1^: 0–75 min, 2%–22% B; 75–80 min, 22%–37% B; 80–85 min, 37%–80% B; 85–90 min, 80% B.

The MS scan was performed at 60K resolution with a mass range of m/z 100 to 1700 at positive mode. The number of parallel accumulation‐serial fragmentation (PASEF) ramps was set as 10, with the total cycle time was 1.17 s. The range of 1/k_0_ values was set to 0.6–1.6 V s cm^−2^, corresponding to the collision energy from 20 to 59 V. MS/MS analysis was performed on the most abundant intense signal exhibiting a charge state from 0 to 5. The dynamic exclusion was set to 0.5 min with a 15 ppm mass window. The raw data files (^*^.d) were directly import to PEAKS Studio (Bioinformatics Solutions, Inc) for qualitative and quantitative analysis of the proteins. MS/MS spectra were searched against the Uniprot Rattus norvegicus (Rat) database (released in December 2021), and both precursor mass tolerance and MS/MS tolerance were set to 15 ppm and 0.02 Da, respectively. Searches were performed with full tryptic digestion and a maximum of 2 missed cleavage was allowed. A false discovery rate (FDR) threshold of 1% was used for both peptide and protein levels and data were processed using the standard legacy PeptideProphet scoring system. The PEAKS software generates a reverse decoy database reversing the protein sequences from the target database. The MitoMiner 4.0 and MitoCarta 2.0 database were used to annotate the mitochondrial proteins. The relative protein abundance ratios were calculated, and permutation tests were performed. Proteins with *p*‐values <0.05 and a significant fold change (>1.3 and <0.769 for up‐regulated and down‐regulated proteins, respectively) were considered as differentially expressed proteins. Function annotations were performed by the DAVID functional annotation clustering tool.

### Transmission Electron Microscopy (TEM)

Hippocampal tissue was fixed with electron microscopy fixative and preserved at 4 °C. Then, the tissue was washed by 0.1 mol L^−1^ phosphate‐buffered saline (PBS, pH 7.4), and fixed with 1% OsO4 in 0.1 mol L^−1^ PBS (pH 7.4) for 2 h at room temperature. The tissue was then dehydrated in ethanol and embedded in resin. The resin embedded tissue was allowed to polymerize in 65 °C oven for 48 h, and then cut into 60 nm thin sections, which were examined by transmission electron microscopy (HT7800, Hitachi, Tokyo, Japan).

### Cell Culture

SK‐N‐SH cells (ATCC Cat# HTB‐11, RRID:CVCL_0531) were routinely cultured in DMEM medium containing 10% fatal bovine serum, 100 units·mL^−1^ penicillin and 100 µg·mL^−1^ streptomycin at 37 °C under a humidified condition of 5% CO_2_. 5000 SK‐N‐SH cells were seeded in 96‐well plates and cultured overnight. Then, different concentrations of NaN_3_ were added to the DMEM cell culture medium for modeling. After 24 h of being cultured with NaN_3_, the cell survival rate was determined by the cell counting kit‐8. The optimal concentration of NaN_3_ was applied to establish the NaN_3_‐induced mitochondrial damage cell model. After that, the administrated cells were pre‐treated with various concentrations of SG in target brain mitochondria based on the established model for 12 h in incubator. Finally, the model was applied on control, model and treated groups were harvested for cell viability, mitochondrial biogenesis assays, metabolomics, western blot, and fluorescence microscopy assays.

### Mitochondrial Biogenesis Assays

Metabolic data on energy production in cells were examined using Agilent Seahorse XFe96 analyzer (Santa Clara, CA). Briefly, cells plated at an optimized seeding density of 20 000 cells/well in 200 µL of DMEM media. The cultured cells were divided randomly into different groups: control group (blank control), model group (NaN_3_‐induced mitochondrial damage cells), and SG group. Prior to cell DMEM media was removed and replaced with Seahorse XF assay media which containing 2 mmol L^−1^ glutamine, 10 mmol L^−1^ glucose and 2 mmol L^−1^ sodium pyruvate. On the day of measurement, cells were washed with XFe96 media and incubated in a CO_2_‐free incubator at 37 °C for 2 h to establish equilibration prior to loading.

The extracellular acidification rates (ECARs) of cells were measured using Agilent Seahorse XF Cell Glycolytic Stress Test Kit, these compounds (glucose, oligomycin, and 2‐deoxy‐glucose (2‐DG)) were serially injected to measure basal glycolysis and glycolytic capacity, respectively.

The oxygen consumption rate (OCR) of cells was measured using Agilent Seahorse XF Cell Mito Stress Test Kit and Substrate Oxidation Stress Test Kit, these compounds (oligomycin, FCCP, and a mix of rotenone and antimycin A) were serially injected to measure basal respiration, maximal respiration, spare respiratory capacity, ATP‐linked respiration, respectively.

The cellular rate of ATP production was measured in real time using an Agilent Seahorse Real‐Time ATP Rate Assay Kit. After the assessment of basal respiration, metabolic modulators (oligomycin and a mix of rotenone and antimycin A) that when serially injected, allow the calculation of the mitochondrial and glycolytic ATP production rates, and providing a quantitative insight into phenotype of cellular energy poise.

### Cell Treatment for Metabolic Flux Analysis

SK‐N‐SH cells were seeded in growth media at 5 × 105 cells per dish and divided into four groups: ^12^C control group, ^13^C control group, NaN_3_‐induced mitochondrial damage model group, and SG + NaN_3_ treatment group, especially. After 24 h of cell culture, the media in each dish except for the ^12^C control group were exchanged with an isotope tracing media consisting of glucose‐free DMEM supplemented with 10 mmol L^−1^ [^13^C_6_]‐glucose. Then, SG (1 µmol L^−1^) was added in the culture medium of the SG + NaN_3_ treatment group after 4 h. After another 4 h of cell culture, 1 mmol L^−1^ NaN_3_ was added to the NaN_3_ model group and SG + NaN_3_ treatment group, especially. Finally, cells were harvested for metabolic flux analysis after 12 h cultured of ^13^C control group, NaN_3_ model group, and SG + NaN_3_ treatment group. Cells in the ^12^C control group were incubated with media supplemented with 10 mmol L^−1^ unlabeled glucose and harvested at the same time. This experiment was repeated independently three times.

### UHPLC‐HRMS Metabolomics and Isotope Tracing Metabolic Flux Analysis

The culture medium was gently aspirated, and cells were collected in a glass centrifuge tube. After adding 20 µL of d_4_‐succinate internal standard solution and 1 mL of cooled methanol: water (80:20, v/v) solution, the cells were extracted by ultrasound and vortex for 1 min. The centrifuge tubes were incubated at −80°C for 12 h to aid cell division and protein precipitation, exposed to ultrasound, and vortexed again for 1 min. After centrifugation at 12 000 g for 5 min at 4°C, the supernatant was transferred to another glass centrifuge tube and dried under a gentle nitrogen stream and then kept at −80°C. Dried extracts were re‐suspended in 120 µL of acetonitrile: water (90:10, v/v) solution. After centrifugation at 12 000 g for 5 min at 4°C, a 100 µL volume was transferred to auto‐sampler vials and analyzed for metabolomics and isotope tracing metabolic flux analysis by UHPLC‐HRMS system.

The experiment used a 1290 Infinity II ultra‐high performance liquid chromatography (UHPLC) system coupled to a 6550 iFunnel quadrupole time‐of‐flight (Q‐TOF) mass spectrometer equipped with a dual AJS electrospray ionization source (Agilent Technologies). Metabolites were separated on a Waters XBridge Amide column (100 × 2.1 mm, 3.5 µm particle size) (HILIC system). Sample analysis in negative ionization mode was performed using water containing 0.1% ammonium hydroxide and 10 mmol L^−1^ ammonium acetate as mobile phase A and 95% acetonitrile with 0.1% ammonium hydroxide and 10 mmol L^−1^ ammonium acetate as mobile phase B. The elution gradient used was as follows: isocratic step at 100% B for 5 min, 100%–95% B in 6 min, maintained at 95% B for 10 min, 95%–74% B in 20 min, 74%–53% B in 23 min, and 53%–100% B in 24 min, and then, the column was equilibrated at initial conditions for 6 min. The flow rate was 0.3 mL min^−1^, the injection volume was 10 µL, and the column oven was maintained at 35 °C. The metabolite database of the TCA cycle, glycolysis, pentose phosphate pathway, and amino acid metabolism was established using MassHunter Pathways to PCDL and PCDL Manager software. Data analysis and isotopic natural abundance correction were performed within MassHunter VistaFlux, MassHunter Profinder, and MassHunter Quant software, respectively.

### Western Blot Assays

The mitochondrial fraction of SK‐N‐SH cells were isolated as described above, total protein samples from mitochondrial fraction were extracted and the protein content was then determined by using a BCA protein assay kit. Mitochondrial proteins (30 µg) were subjected to sodium dodecyl sulfate‐polyacrylamide gel electrophoresis, and then proteins were transferred onto polyvinylidene difluoride membranes. After blotting, membranes were blocked with 5% nonfat milk, incubated overnight at 4°C with primary antibodies against pyruvate dehydrogenase E2 (Dlat; Abcam, Cat# ab88884, RRID: AB_2091772), ATP synthase subunit O, mitochondrial (Atp5o; Abcam, Cat# ab91400, RRID: AB_2049187) and voltage‐dependent anion‐selective channel 1 (VDAC1; Abcam Cat# ab10523, RRID: AB_297264). Protein blots were incubated with secondary antibody for 1 h at room temperature. VDAC1 served as a loading control in mitochondrial samples. The bands were visualized using the enhanced chemiluminescence (ECL) method, and the relative proteins levels were quantified using Image J software by an investigator blinded to the groups. The primary antibodies against Cytochrome C (Cat#4272), Bcl‐2 (D17C4, Cat#3498), Bax (2D2, Cat#89477), and β‐Actin (8H10D10, Cat#3700) for evaluation of mitochondrial induced apoptosis were from Cell Signaling Technology. The primary antibodies against Caspase‐1 (Cat#81482‐1‐RR) and GSDMD (Cat#ab210070) for evaluation of mitochondrial induced pyroptosis were from Proteintech Group Inc and Abcam, respectively. The experimental procedure was the same as above.

### Fluorescence Microscopy Assays

After days of culture, medium was removed and attached cells were stained with fresh DMEM supplement with MitoTracker Deep Red (diluted 1:5000 in DMEM). Cells were incubated for 30 min at 37 ˚C and 5% CO_2_, and then fixed with a 4% para‐formaldehyde solution for 30 min at room temperature. After fixation, cells were washed with PBS and subsequently mounted with mounting media containing DAPI. Fluorescence microscopy images were acquired on a Leica TCS SP8 confocal laser scanning microscope.

### Drug Affinity Responsive Target Stability

Drug affinity Response target Stability (dart), based on the principle that the protease sensitivity of the target protein was reduced upon drug binding, was a commonly used method for identifying small molecular‐protein interactions. As it does not require modification of the drug and does not depend on the mechanism of drug action, it has universal applicability.SK‐N‐SH cells were lysed with M‐PER supplemented with protease and phosphatase inhibitors. Then a BCA protein assay was performed to determine the protein concentration of cell lysate. DMSO (1 µL) or 100x different concentration SG stock solution was added into the cell lysate of 99 µL and incubated at room temperature for 2 h. After incubation, the samples were divided into 20 µL for use. 2 µL protease solution (predetermined appropriate protease concentration, protease: protein = 1:3000) was added to the sample tube, and 2 µL buffer was added to the control group instead of protease. Incubate at room temperature for 15 min. To stop proteolysis, a protease inhibitor solution was added to each sample, mixed well, and iced for 10 min. Finally, 6 µL 5 x electrophoresis sample buffer was added, and the expression of target proteins was analyzed by western blotting to verify the binding of protein to SG.

### Cellular Thermal Shift Assay, *CTESA*

CETSA was an experiment to detect the binding efficiency of intracellular drugs and target proteins. Its principle was that the target proteins usually become stable when combined with drug molecules. That is, with the increase of temperature, the protein will degrade; When protein was combined with drugs, the amount of undegraded protein will increase at the same temperature. We first incubated SG with SK‐N‐SH cells for 1 h (the control group was the same volume of DMSO). Next, cells were collected, and the cells were lysed on ice with M‐PER lysate for 30 min, then evenly divided and heated for 3 min at different temperatures. Then, the cells were freeze‐thawed repeatedly in liquid nitrogen for three times, and the supernatant was collected after centrifugation into a new tube. BCA method was used for protein quantification, and the subsequent operation was the same as western blotting.

### Co‐ IP and LiP‐MS

We added 1 mL cold IP lysis buffer (Thermo Scientific, 87788) to 80%–90% of fused monolayer SK‐N‐SH cells in 100mm^2^ cell culture plate and incubated at 4 °C for 30 min to lysate the cells. Then centrifuge at 10 000 x g at 4 °C for 10 min. The supernatant was transferred into a fresh 1.5 mL conical centrifuge tube, and the protein in cell lysate was quantitatively analyzed by BCA protein assay. Then 800 µg total cell protein was taken into 1.5 mL microcentrifuge tube, SG was added to the protein (1 mmol L^−1^, equal volume of DMSO was added to the control group), and incubated at 37 °C for 1 h. The solution was then added with 0.6 µg PDK2 monoclonal antibodies (a control group containing 0.6 µg of normal rabbit IgG corresponding to the PKD2 antibody of the host) and incubated at 4 °C. After 3 h, protein A/G PLUS Agarose with a 20 µL suspension volume (SANTA CRUZ, sc‐2003) was added and incubated overnight at 4 °C on a rotating device. After incubation, the immunoprecipitates were collected by centrifugation at 2500 rpm at 4 °C for 5 min, and the particles were washed with 1.0 mL PBS for 4 times. Before the final cleaning, the solution containing the particles was divided into two parts. One of the solutions was centrifuged and the supernatant discarded, then the particles were re‐suspended in a 40 µL 1x electrophoresis sample buffer and identified using western blotting. The other was processed for mass spectrometry.

A limited proteolysis step was performed with proteinase K (PK) under native conditions, followed by complete digestion with trypsin under denaturing conditions to generate MS‐measurable peptides. The immunoprecipitated beads extracted under native lysis conditions were treated or not with SG, with preincubated for 1 h at 1 mmol L^−1^. Sample preparation and analysis of control and SG group samples were performed based on Lip‐MS experimental protocols (*n* = 3).

### Stable Knockdown of PDK2 in the SK‐N‐SH Cells

The human gene was cloned into the lentiviral vector pLKO.1‐puro to generate pLKO.1‐puro/PDK2. Lentiviral particles were generated by the co‐transfection of HEK 293T cells with pLKO.1‐puro/PDK2 (alone with empty pLKO.1‐puro as control), pMD2G (Envelope plasmid), and psPAX2 (Packaging plasmid). After 48 h of transfection, the cell culture medium was collected for concentration and finally the virus (PDK2) was obtained. SK‐N‐SH cells were seeded into 6‐well plates at a density of 2×105 cells/well and infected with lentivirus containing either the PDK2 shRNAs or scramble shRNA the next day. After 48 h, the culture medium was replaced with fresh medium containing puromycin for the selection of stable transfectants. Then cells were collected to analyze the expression of PDK2 by western blotting.

### Mitochondrial Membrane Potential Assay

SK‐N‐SH cells were cultured overnight in 6‐well plates and then divided into four groups: NaN_3_‐induced mitochondrial damage model group, and SG (1, 5, 10 µmol L^−1^) + NaN_3_ treatment group, especially. SG was added in the culture medium of the SG + NaN_3_ treatment group after 4 h of cell culture. After another 4 h of cell culture, 1 mmol L^−1^ NaN_3_ was added to the NaN_3_ model group and SG + NaN_3_ treatment group, especially. Then, mitochondrial membrane potential was detected according to the instructions of Mitochondrial Membrane Potential Assay Kit with TMRE (C2001S, Beyotime, Shanghai, China).

### Mobility Shift Assay for Kinase Activity Evaluation

Mobility Shift Assays/IMAP assays (Carna Biosciences, Kobe, Japan) directly monitor phosphorylation by measuring the non‐phosphorylated and phosphorylated substrate in the kinase reaction. The percentage inhibition ratio of SG, against PDK2 (Carna Cat. No. 10–140) and PDK4 (Carna Cat. No. 10–125), were tested with dichloroacetic acid (DCA) as the positive control. Full‐length human PDK2 [1‐407(end) amino acids of accession number NP_002602.2] was expressed as N‐terminal GST‐fusion protein (74 kDa) using baculovirus expression system. Full‐length human PDK4 [1‐411(end) amino acids of accession number NP_002603.1] was expressed as N‐terminal GST‐fusion protein (73 kDa) using E.coli expression system. GST‐PDHK2 was purified by using glutathione sepharose chromatography. GST‐PDHK4 was purified by using glutathione affinity chromatography.

### Molecular Docking

All docking experiments were carried out using Discovery Studio 2016 software, and crystal structures of PDK2 with ligands binding to ATP‐binding domain (PDB:2Q8I), lipoamide‐binding domain (PDB:2BU6), and CoA‐binding domain (PDB:2BU7) were obtained from the RCSB Protein Data Bank. Scutellarin was used for ligand molecule. Protein preparation and minimization were carried out using the Protein Preparation tool in Discovery Studio. Hydrogen atoms were added to the protein‐ligand complex under the CHARMM force field. All water molecules were removed, and the pH environment was adjusted to neutral. The original ligand‐binding pockets were chosen as the active site for docking. The Libdock and CDOCKER scores were obtained by the Libdock and CDOCKER algorithms with the default parameters.

### Apoptosis Analysis

SK‐N‐SH cells were cultured in 6‐well plates at a density of 3 × 105 cells per well. Overnight, cells were treated with different concentrations of SG (0, 1, 5, and 10 µmol L^−1^) After another 4 h of cell culture, 1 mmol L^−1^ NaN_3_ was added to each group, except for the control group. The next day, cells were trypsinized without EDTA and stained according to the instructions of FITC Annexin V Apoptosis Detection Kit (BD Pharmingen, USA). Data were acquired by BD FACS verse flow cytometer (BD Biosciences, NJ, USA) and analyzed with FlowJo software.

### Statistical Analysis

Statistical analyses were performed using SPSS software program (version 19.0, Chicago, IL, USA). All data represent biological replicates (n) and were expressed as mean ± SEM. One‐way analysis of variance (ANOVA) with the post hoc test followed by Student's *t*‐test was performed. P values < 0.05 were considered to be statistically significant.

## Author Contributions

N.S., Z.Z., and H.Z. contributed equally to this work. N.S. performed conceptualization, formal analysis, investigation, project administration, visualization, and wrote original draft. Z.Z. performed formal analysis and investigation. H.Z. performed data curation and formal analysis. C.M. performed investigation and validation. M.L., Z.W., and L.W. performed investigation. J.J. performed conceptualization, investigation, resources, and supervision. J.Z. performed conceptualization, investigation, project administration, resources and supervision.

## Conflict of Interest

The authors declare no conflict of interest.

## Supporting information

Supporting Information

## Data Availability

The data that support the findings of this study are available in the supplementary material of this article.
