# Supplementary material for: Scutellarin Rescued Mitochondrial Damage through Ameliorating Mitochondrial Glucose Oxidation via the Pdk‐Pdc Axis
Source: Adv Sci (Weinh). 2023 Sep 26;10(32):2303584. doi: 10.1002/advs.202303584 (PMC10646256; doi:10.1002/advs.202303584)
Supplement: Supplementary file 1 — Supporting Information [file ADVS-10-2303584-s001.pdf]

ADVANCED SCIENCE
Open Access

Supporting Information

for *Adv. Sci.*, DOI 10.1002/advs.202303584

Scutellarin Rescued Mitochondrial Damage through Ameliorating Mitochondrial Glucose Oxidation via the Pdk-Pdc Axis

Ning Sheng, Zhihui Zhang, Hao Zheng, Congyu Ma, Menglin Li, Zhe Wang, Lulu Wang, Jiandong Jiang and Jinlan Zhang**

Supplementary material

Scutellarin rescued mitochondrial damage through ameliorating mitochondrial glucose oxidation *via* the PDK-PDC axis

Ning Sheng [a, #], Zhihui Zhang [a, #], Hao Zheng [a, #], Congyu Ma [a], Menglin Li [a], Zhe Wang [a], Lulu Wang [b], Jiandong Jiang [a, b, *], Jinlan Zhang [a, *]

a, State Key Laboratory of Bioactive Substance and Function of Natural Medicines, Institute of Materia Medica, Chinese Academy of Medical Science and Peking Union Medical College, Beijing, 100050, China.

b, Institute of Medicinal Biotechnology, Chinese Academy of Medical Science and Peking Union Medical College, Beijing, 100050, China.

[#] Co-first author: Ning Sheng, Zhihui Zhang and Hao Zheng

* Corresponding author: Prof. Jiandong Jiang and Prof. Jinlan Zhang

Tel.: +86-10-83154880; Fax: +86-10-63017757

E-mail: zhjl@imm.ac.cn (Jinlan Zhang)

E-mail: jiang.jdong@163.com (Jiandong Jiang)

CONTENT

Animal experiments and drug administration

Supplementary Figure S1 The impact of SG on mitochondrial parameters and proteins associated with oxidative phosphorylation activity.

Supplementary Figure S2 The cytotoxicity evaluation of SG to SK-N-SH cells

Supplementary Figure S3 The validation model and the permutation test of the OPLS-DA model compared control group with model group

Supplementary Figure S4 The validation model and the permutation test of the OPLS-DA model compared model group with SG group

Supplementary Figure S5 The original diagrams of three biological repeats of the western blotting experiment of *Dlat* and *ATP5o* (3 replicates)

Supplementary Figure S6 The LibDock score or CDOCKER_Interation_Energy of SG and the ligands

Supplementary Figure S7 The specific amino acids that SG bind to each domain through hydrogen bonding

Supplementary Figure S8 The western blot of the DARTS experiments

Supplementary Figure S9 The western blot of the CETSA experiments (2 replicates)

Supplementary Figure S10 The western blot of the control group, vehicle group, and shPDK2 group SK-N-SH cells

Supplementary Figure S11 The western blot of the co-IP experiment

Supplementary Figure S12 The proteomics study to explore the effect of SG on mitochondrial damaged cells, **a**, proteomics analysis of mitochondria damage model group and SG group; **b**, mitochondrial protein with FC greater than 2 compared SG with model group; **c**, protein-protein interaction enrichment analysis of the 28 proteins with FC greater than 2 compared SG with model group; **d**, the biological process analysis of the mitochondrial proteins with FC greater than 1.5 compared SG with model group

Supplementary Figure S13 The western blot of apoptosis-related proteins of the vehicle group, and shPDK2 group SK-N-SH cells

Supplementary Figure S14 The western blot of pyroptosis-related proteins of the vehicle group, and shPDK2 group SK-N-SH cells

Supplementary Table S1 Mitochondrial proteins identified from rat brain tissue

Supplementary Table S2 Mitochondrial DEMPs both belong of the model vs sham-operation group and SG vs model group in rat brain tissue

Supplementary Table S3 The identified peptides associated with the PDK2 binding pockets

Animal experiments and drug administration

Male Sprague-Dawley rats (6 weeks old) weighing 200 ± 10 grams (g) were purchased from Beijing Vital River Laboratory Animal Co. Ltd. (Beijing, China). Rats were housed in environmentally controlled facilities on a 12:12-hour light-dark cycle at constant temperature (22 °C ± 2 °C) and humidity (50 ± 10%) with free access to food and water. Animal experimentation began after 2 weeks of acclimation. The rats were randomly assigned to receive permanent bilateral occlusion of common carotid arteries induction (CCH model) or sham operation as previously described and sodium pentobarbital was chosen for this study as it was the most suitable anesthetic for the measurement of systemic responses to permanent bilateral occlusion of common carotid arteries. Briefly, rats were anesthetized with sodium pentobarbital [50 $mg \cdot kg^{-1}$, intraperitoneal (i.p.)] to ensure that the experimental subjects suffered minimal discomfort, and a neck ventral midline incision was made, then both common carotid arteries were exposed and gently ligated with 5-0 silk sutures. Sham operation group rats received the similar surgical procedures without carotid artery ligation, which served as a control. The body temperature was maintained at 37°C throughout surgery, and operation time per animal did not exceed 15 min. After surgery, rats were placed in a recovery area with thermal support until fully recovered. In the animal experiment, the sham operation group (n=10) served as a control and the CCH modeled rats were randomly divided into the model group (n=10) and scutellarin group (n=10, 111.3 $mg \cdot kg^{-1}$). Nimodipine, a well validated drug that is effective in the CCH pharmacological model, was used as a positive control to evaluate the efficacy of pharmacotherapies in two independent animal experiments (n=6). The doses used *in vivo* are relevant for clinical translation. Rats in drug-treated groups were received daily orally administered with the respective drugs for 4 weeks, and rats in other groups were received daily orally administered an equal amount of vehicle.

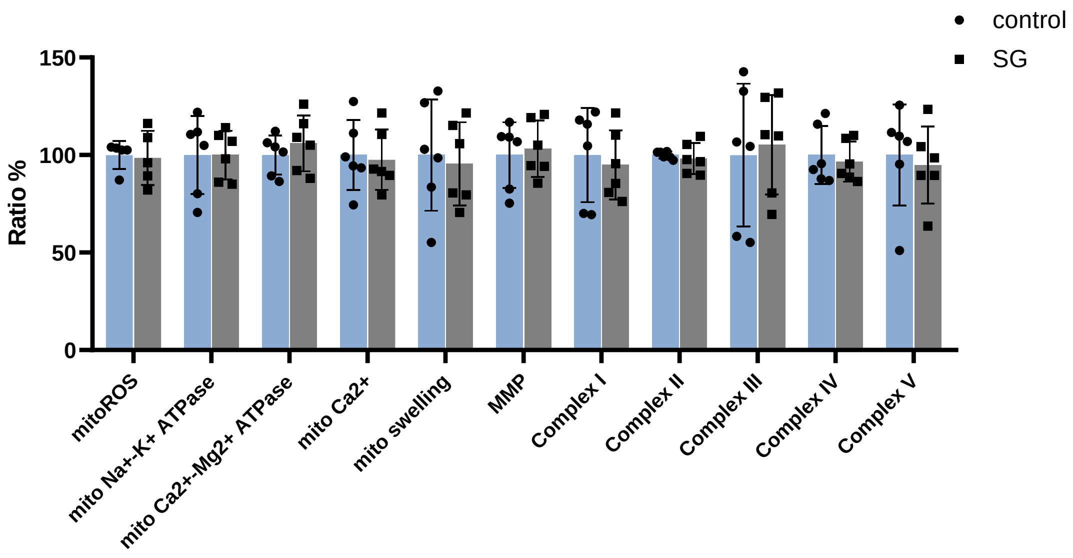

Supplementary Figure S1 the impact of SG on mitochondrial parameters and proteins associated with oxidative phosphorylation activity.

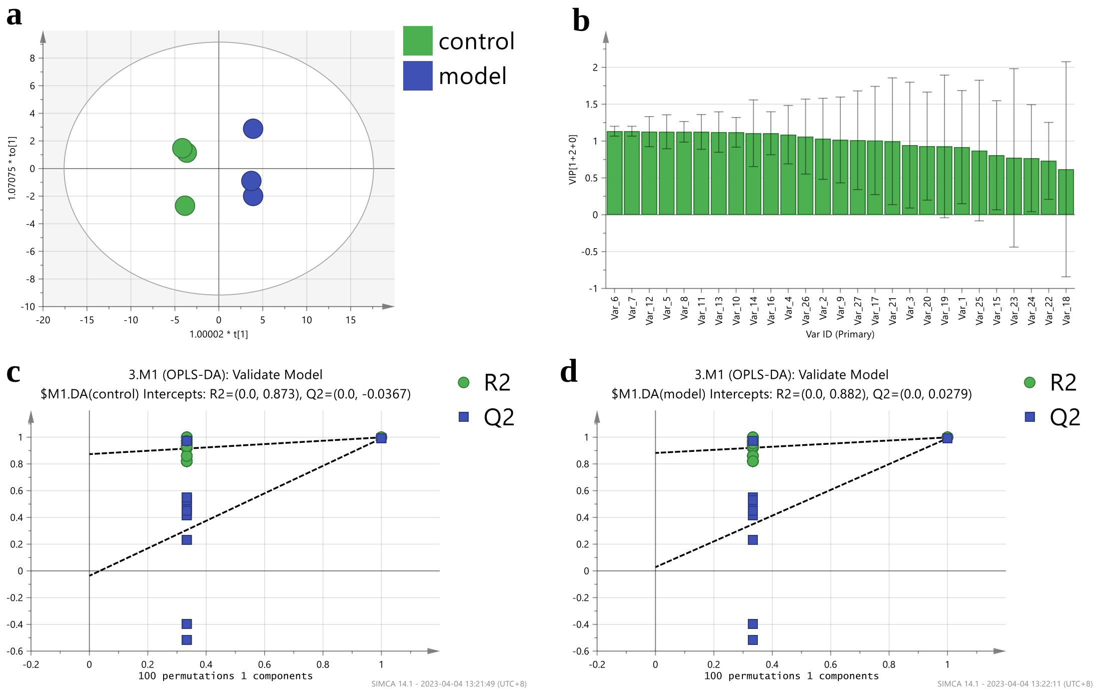

Supplementary Figure S2 The validation model and the permutation test of the OPLS-DA model compared control group with model group

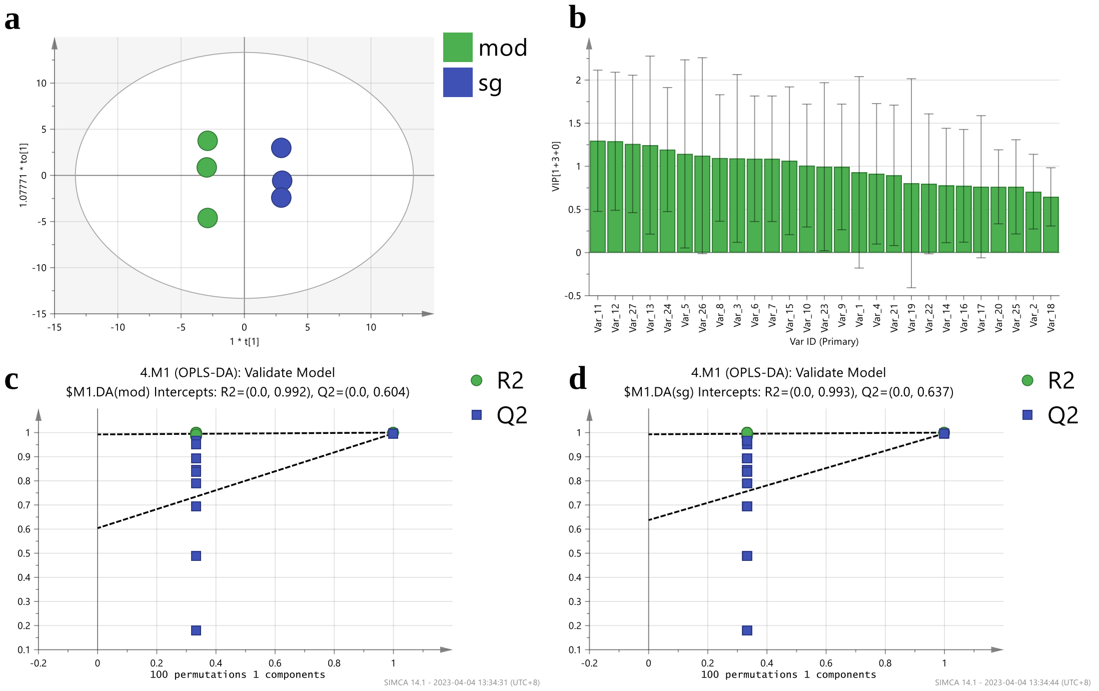

Supplementary Figure S3 The validation model and the permutation test of the OPLS-DA model compared model group with SG group

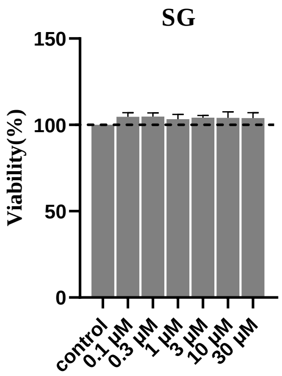

Supplementary Figure S4 the cytotoxicity evaluation of SG to SK-N-SH cells

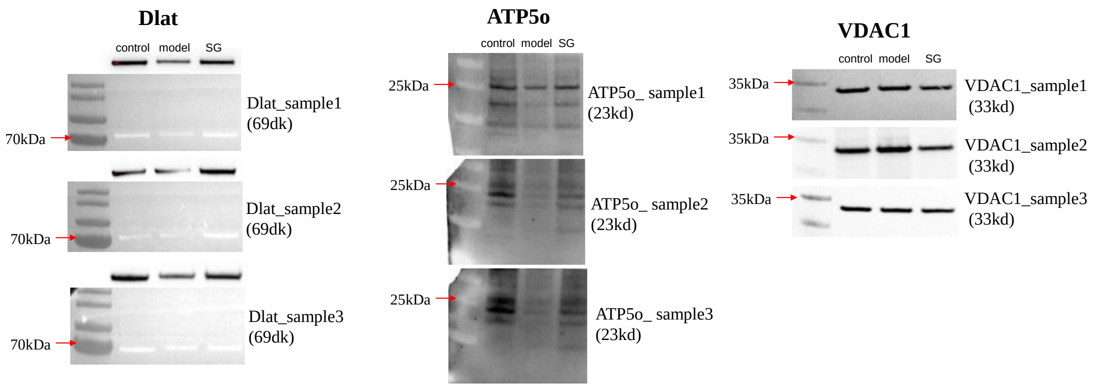

Supplementary Figure S5 The original diagrams of three biological repeats of the western blotting experiment of *Dlat* and *ATP5o* (3 replicates)

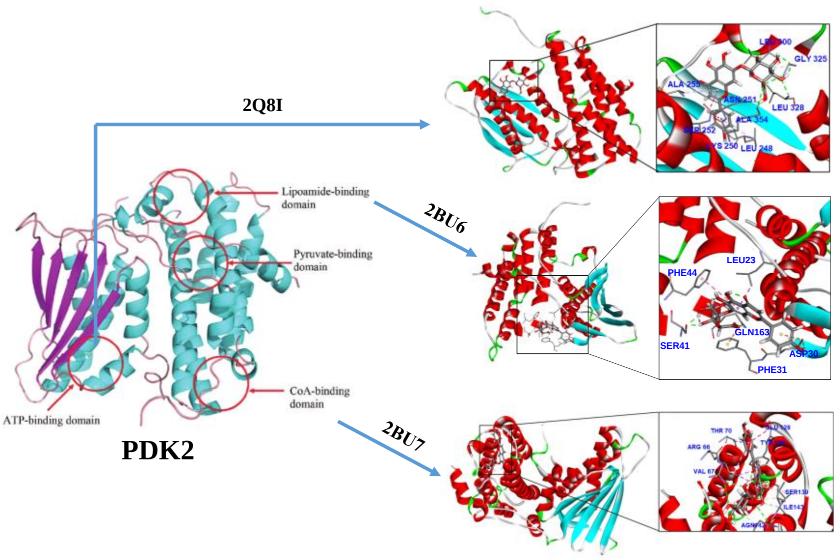

2Q8I

Ligand	LibDock score
Radicicol	95.70
SG	128.98

2BU6

Ligand	CDOCKER_Interation_Energy
AZ12	41.27
SG	47.29

2BU7

Ligand	LibDock score
Pfz3	124.50
SG	147.20

Supplementary Figure S6 The LibDock score or CDOCKER_Interation_Energy of SG and the ligands

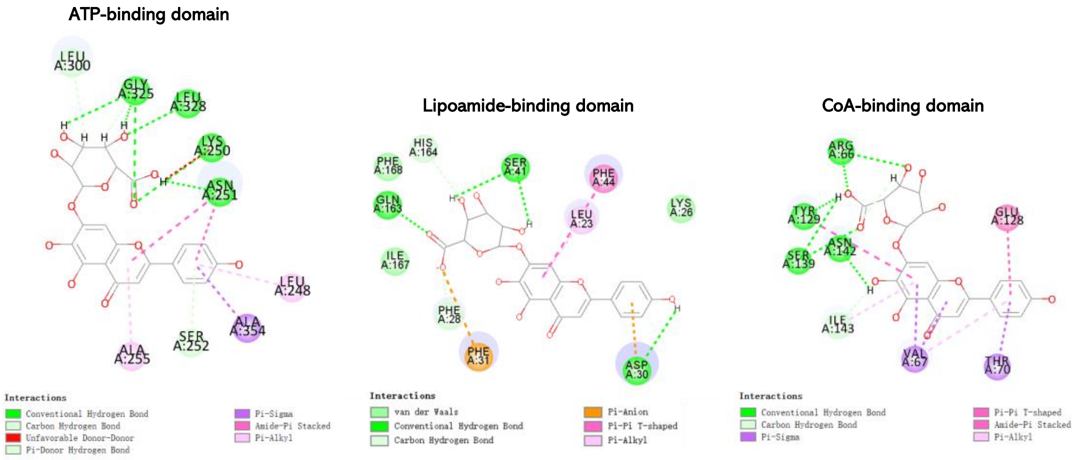

Supplementary Figure S7 The specific amino acids that SG bind to each domain through hydrogen bonding

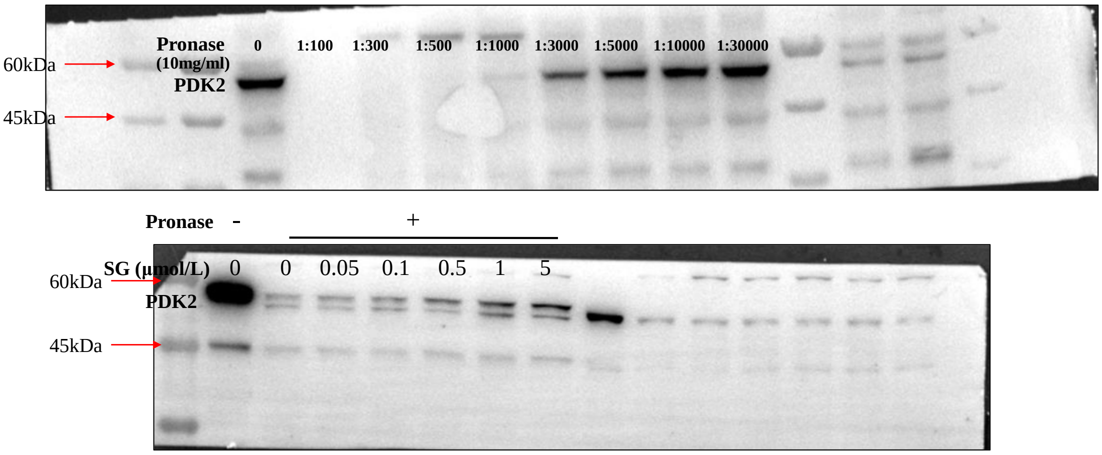

Supplementary Figure S8 The western blot of the DARTS experiments

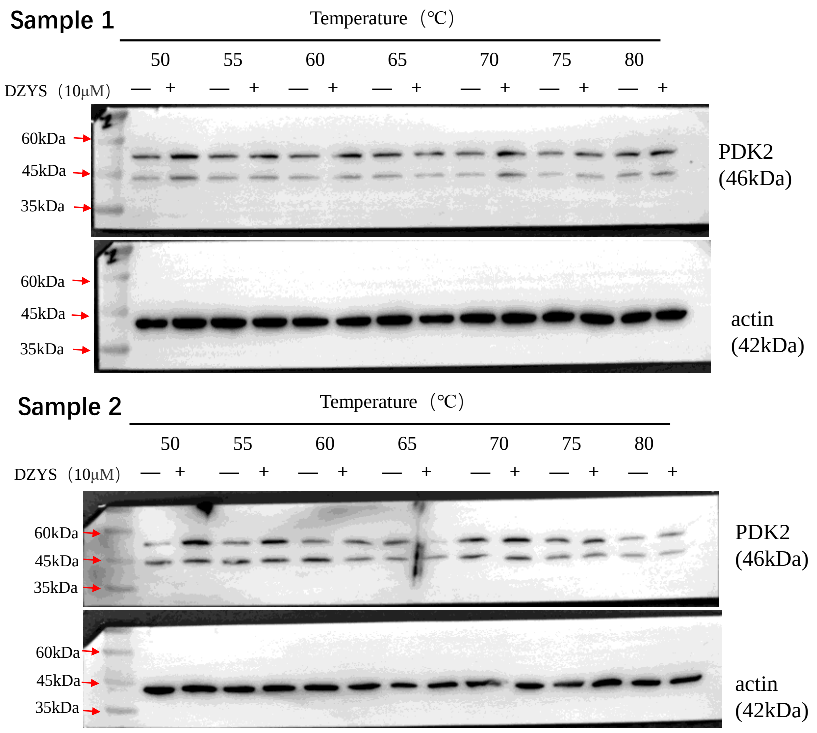

Supplementary Figure S9 The western blot of the CETSA experiments (2 replicates)

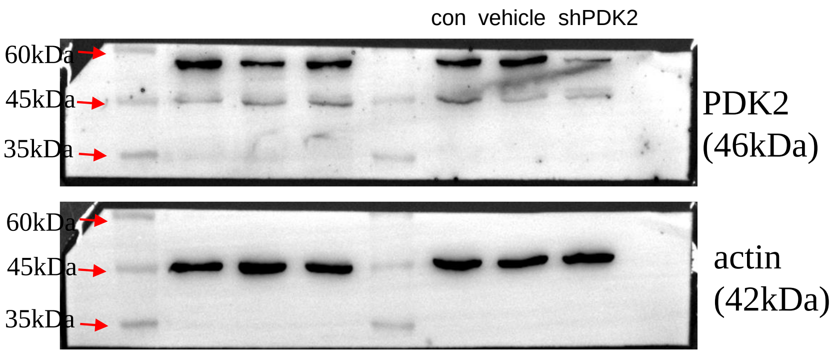

Supplementary Figure S10 The western blot of the control group, vehicle group, and shPDK2 group SK-N-SH cells

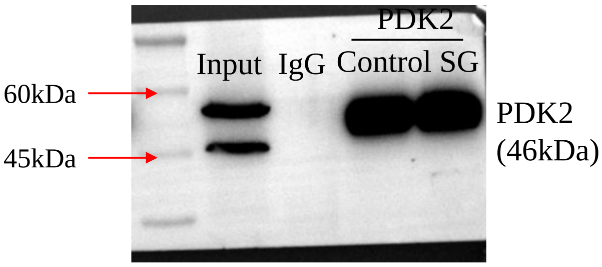

Supplementary Figure S11 The western blot of the co-IP experiment

a

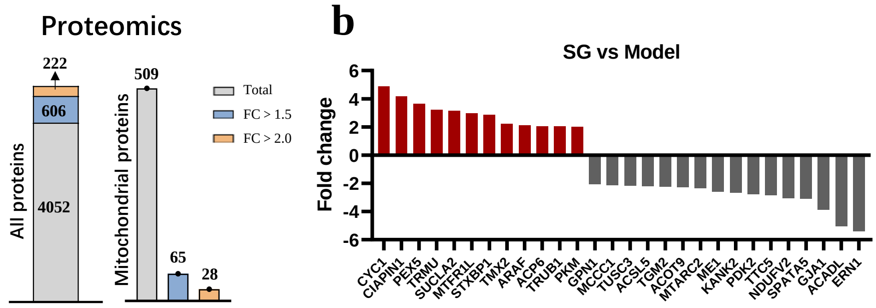

b

c

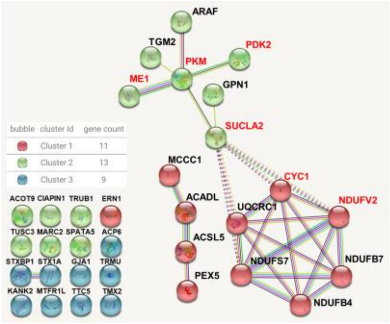

d

Biological process	Counts	-Log P
positive regulation of apoptotic process	7	3.1
regulation of apoptotic process	6	3.0
mitochondrion organization	4	2.6
apoptotic process	8	2.5
lysobisphosphatidic acid metabolic process	2	2.2
positive regulation of pyruvate dehydrogenase activity	2	2.0
carnitine metabolic process, CoA-linked	2	2.0
negative regulation of neuron death	3	1.7
cellular protein localization	3	1.7
leucine catabolic process	2	1.6
cellular response to starvation	3	1.6
mRNA cleavage	2	1.6
negative regulation of apoptotic process	6	1.5
mitochondrial translation	3	1.4
programmed cell death	2	1.3
response to oxidative stress	3	1.2
mitochondrial fission	2	1.2
long term synaptic depression	2	1.2
regulation of glucose metabolic process	2	1.1
protein tetramerization	2	1.1
DNA metabolic process	2	1.1
acyl-CoA metabolic process	2	1.1

Supplementary Figure S12 The proteomics study to explore the effect of SG on mitochondrial damaged cells, **a**, proteomics analysis of mitochondria damage model group and SG group; **b**, mitochondrial protein with FC greater than 2 compared SG with model group; **c**, protein-protein interaction enrichment analysis of the 28 proteins with FC greater than 2 compared SG with model group; **d**, the biological process analysis of the mitochondrial proteins with FC greater than 1.5 compared SG with model group

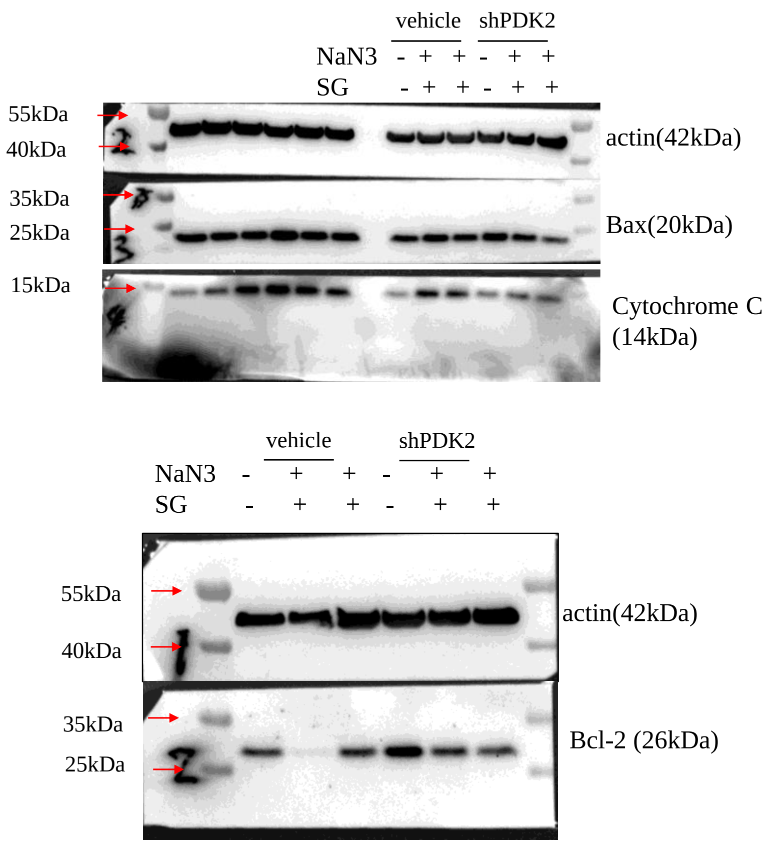

Supplementary Figure S13 The western blot of apoptosis-related proteins of the vehicle group, and shPDK2 group SK-N-SH cells

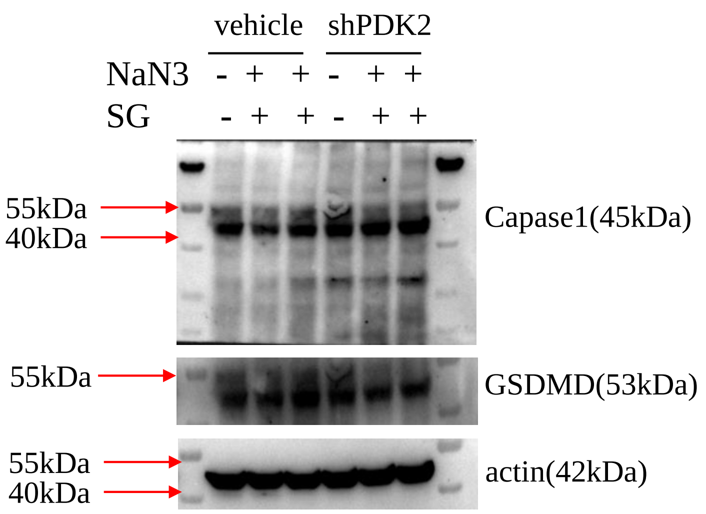

Supplementary Figure S14 The western blot of pyroptosis-related proteins of the vehicle group, and shPDK2 group SK-N-SH cells

Supplementary Table S1 Mitochondrial proteins identified from rat brain tissue

UniProt Accession	Ensembl Primary Identifier	Gene Symbol	Mito Evidence Mass-Spec Experiments	Mito Evidence GO Annotation	Mito Evidence IMPI
A0A096MIV5	ENSRNOG00000010609	Abcf2	0	FALSE	Predicted mitochondrial
A0A096MJF7	ENSRNOG00000003063	Phka1	1	FALSE	Predicted mitochondrial
A0A096MJN4	ENSRNOG00000007367	4-Sep	0	FALSE	Known mitochondrial
A0A096MKB0	ENSRNOG00000016539	Rab24	0	FALSE	Known mitochondrial
A0A096MKG5	ENSRNOG00000054157	Nadk2	2	TRUE	Known mitochondrial
A0A0A0MXU4	ENSRNOG00000005123	Emc2	2	FALSE	Predicted mitochondrial
A0A0A0MXW1	ENSRNOG00000009928	Bckdhb	5	TRUE	Known mitochondrial
A0A0A0MXX7	ENSRNOG00000015213	Fxn	2	TRUE	Known mitochondrial
A0A0A0MXZ0	ENSRNOG00000018343	Isca1	1	FALSE	Known mitochondrial
A0A0A0MY00	ENSRNOG00000020624	Acadsb	3	FALSE	Known mitochondrial
A0A0A0MY09	ENSRNOG00000026963	Hsp90b1	4	FALSE	Predicted mitochondrial
A0A0A6YYM0	ENSRNOG00000026745	Acsl6	2	TRUE	Predicted mitochondrial
A0A0G2JSG6	ENSRNOG00000000122	Ak2	6	TRUE	Known mitochondrial
A0A0G2JSH2	ENSRNOG00000001736	Bdh1	9	TRUE	Known mitochondrial
A0A0G2JSH9	ENSRNOG00000003520	Prdx2	1	FALSE	Predicted mitochondrial
A0A0G2JSI1	ENSRNOG00000004027	Aldh9a1	4	FALSE	Known mitochondrial
A0A0G2JSR0	ENSRNOG00000019277	Vdac3	5	TRUE	Known mitochondrial
A0A0G2JSS8	ENSRNOG00000021125	Prdx5	6	TRUE	Known mitochondrial
A0A0G2JSY3	ENSRNOG00000011561	Nln	4	FALSE	Known mitochondrial
A0A0G2JSZ9	ENSRNOG00000047816	Ccs	0	FALSE	Known mitochondrial
A0A0G2JT25	ENSRNOG00000050315	Dcxr	2	FALSE	Known mitochondrial
A0A0G2JTG1	ENSRNOG00000051624	Hspe1	6	TRUE	Known mitochondrial
A0A0G2JTL5	ENSRNOG00000019372	Pc	7	TRUE	Known mitochondrial
A0A0G2JTN3	ENSRNOG00000027012	Usp54	0	FALSE	Predicted mitochondrial
A0A0G2JTP0	ENSRNOG00000043060	Dnajc4	0	FALSE	Predicted mitochondrial
A0A0G2JTY6	ENSRNOG00000003392	Grsf1	2	TRUE	Known mitochondrial
A0A0G2JU15	ENSRNOG00000009484	Ptcd3	2	TRUE	Known mitochondrial
A0A0G2JU49	ENSRNOG00000042977	Mtx1	1	TRUE	Known mitochondrial
A0A0G2JUM8	ENSRNOG00000052701	Coa6	0	TRUE	Known mitochondrial
A0A0G2JUS7	ENSRNOG00000005377	Tmem11	3	TRUE	Known mitochondrial
A0A0G2JUZ5	ENSRNOG00000011599	Gldc	1	TRUE	Known mitochondrial
A0A0G2JV65	ENSRNOG00000008195	Ywhaz	4	TRUE	Predicted mitochondrial
A0A0G2JV81	ENSRNOG00000054775	Fkbp11	1	FALSE	Predicted mitochondrial
A0A0G2JV84	ENSRNOG00000059579	Gpt2	3	FALSE	Known mitochondrial
A0A0G2JVC8	ENSRNOG00000054549	Lss	2	FALSE	Predicted mitochondrial
A0A0G2JVF1	ENSRNOG00000008463	Rundc3b	0	FALSE	Predicted mitochondrial
A0A0G2JVH4	ENSRNOG00000009097	Immt	8	TRUE	Known mitochondrial

A0A0G2JVK4	ENSRNOG00000011561	Nln	4	FALSE	Known mitochondrial
A0A0G2JVL6	ENSRNOG00000005668	Ndufa8	4	TRUE	Known mitochondrial
A0A0G2JVM6	ENSRNOG00000011914	Dtnb	0	FALSE	Predicted mitochondrial
A0A0G2JW34	ENSRNOG00000036894	Cisd3	1	FALSE	Known mitochondrial
A0A0G2JW51	ENSRNOG00000029194	Dhx30	1	TRUE	Known mitochondrial
A0A0G2JWY8	ENSRNOG00000013479	Stard7	1	FALSE	Known mitochondrial
A0A0G2JWZ9	ENSRNOG00000021384	Ankrd44	0	FALSE	Predicted mitochondrial
A0A0G2JX67	ENSRNOG00000007326	Prepl	2	FALSE	Known mitochondrial
A0A0G2JXH2	ENSRNOG00000020688	Ccdc51	4	FALSE	Known mitochondrial
A0A0G2JY43	ENSRNOG00000002342	Aldh3a2	6	FALSE	Known mitochondrial
A0A0G2JYU2	ENSRNOG00000019970	mrpl11	1	TRUE	Known mitochondrial
A0A0G2JYY6	ENSRNOG00000029571	Coq10a	0	TRUE	Known mitochondrial
A0A0G2JZ68	ENSRNOG00000056223	Tfb1m	1	TRUE	Known mitochondrial
A0A0G2JZA2	ENSRNOG00000006593	Grpel1	5	TRUE	Known mitochondrial
A0A0G2JZF6	ENSRNOG00000055079	Ndufaf2	0	TRUE	Known mitochondrial
A0A0G2JZH8	ENSRNOG00000006947	Pdhx	3	FALSE	Known mitochondrial
A0A0G2JZI2	ENSRNOG00000002393	Eprs	1	FALSE	Predicted mitochondrial
A0A0G2JZS5	ENSRNOG00000007946	Bcl2l1	2	TRUE	Known mitochondrial
A0A0G2K038	ENSRNOG00000018939	Rexo2	1	TRUE	Known mitochondrial
A0A0G2K059	ENSRNOG00000045920	Mcu	1	TRUE	Known mitochondrial
A0A0G2K0L0	ENSRNOG00000011311	Papss1	0	FALSE	Predicted mitochondrial
A0A0G2K0P8	ENSRNOG00000055089	Slc44a1	0	TRUE	Known mitochondrial
A0A0G2K0T5	ENSRNOG00000043436	Micu1	4	TRUE	Known mitochondrial
A0A0G2K0V3	ENSRNOG00000002378	Bpnt1	2	FALSE	Predicted mitochondrial
A0A0G2K121	ENSRNOG00000021725	Mlec	3	FALSE	Predicted mitochondrial
A0A0G2K1N9	ENSRNOG00000060120	Selenoo	1	TRUE	Predicted mitochondrial
A0A0G2K1W1	ENSRNOG00000056009	Rab11fip5	0	TRUE	Known mitochondrial
A0A0G2K1W9	ENSRNOG00000019036	Ldhd	1	TRUE	Known mitochondrial
A0A0G2K261	ENSRNOG00000002368	Iars2	2	TRUE	Known mitochondrial
A0A0G2K2M2	ENSRNOG00000015428	Mff	2	FALSE	Known mitochondrial
A0A0G2K2Q2	ENSRNOG00000055408	Gcat	3	TRUE	Known mitochondrial
A0A0G2K2T2	ENSRNOG00000056332	Miga1	0	FALSE	Predicted mitochondrial
A0A0G2K2U7	ENSRNOG00000015124	Gpam	4	TRUE	Known mitochondrial
A0A0G2K350	ENSRNOG00000018914	Napg	0	TRUE	Known mitochondrial
A0A0G2K398	ENSRNOG00000013604	Gpx4	1	TRUE	Known mitochondrial
A0A0G2K3F1	ENSRNOG00000021324	RGD1565059	0	FALSE	Known mitochondrial
A0A0G2K3J4	ENSRNOG00000016388	Sphkap	1	FALSE	Known mitochondrial
A0A0G2K3K1	ENSRNOG00000028556	Usp30	1	TRUE	Known mitochondrial
A0A0G2K3K2	ENSRNOG00000034254	Actb	4	FALSE	Known mitochondrial
A0A0G2K3U1	ENSRNOG00000019240	Ampd2	1	FALSE	Predicted mitochondrial
A0A0G2K3V4	ENSRNOG00000003359	Ogt	0	FALSE	Known mitochondrial
A0A0G2K3W1	ENSRNOG00000053145	Vwa8	2	FALSE	Known mitochondrial
A0A0G2K459	ENSRNOG00000058658	Mtch2	2	TRUE	Known mitochondrial

A0A0G2K466	ENSRNOG00000019760	Oxnad1	2	TRUE	Known mitochondrial
A0A0G2K478	ENSRNOG00000011617	Dguok	2	TRUE	Known mitochondrial
A0A0G2K4A0	ENSRNOG00000056009	Rab11fip5	0	TRUE	Known mitochondrial
A0A0G2K4C6	ENSRNOG00000017311	Me3	0	TRUE	Known mitochondrial
A0A0G2K4T8	ENSRNOG00000013876	Mipep	3	TRUE	Known mitochondrial
A0A0G2K4Y1	ENSRNOG00000003855	Dnaja3	5	TRUE	Known mitochondrial
A0A0G2K502	ENSRNOG00000015582	Me2	1	TRUE	Known mitochondrial
A0A0G2K513	ENSRNOG00000021096	Tmem143	0	TRUE	Predicted mitochondrial
A0A0G2K5E7	ENSRNOG00000016924	Acly	3	FALSE	Known mitochondrial
A0A0G2K5F1	ENSRNOG00000021174	Macrod1	4	FALSE	Known mitochondrial
A0A0G2K5L2	ENSRNOG00000018450	LOC100911440	2	TRUE	Known mitochondrial
A0A0G2K642	ENSRNOG00000013766	Acaa2	10	TRUE	Known mitochondrial
A0A0G2K6D5	ENSRNOG00000016511	Pitrm1	2	TRUE	Known mitochondrial
A0A0G2K6E2	ENSRNOG00000001890	Txnrd2	4	TRUE	Known mitochondrial
A0A0G2K6E7	ENSRNOG00000030334	Adck5	1	FALSE	Predicted mitochondrial
A0A0G2K6H2	ENSRNOG00000047708	Gstz1	3	TRUE	Known mitochondrial
A0A0G2K6J5	ENSRNOG00000054140	Myl6	4	FALSE	Predicted mitochondrial
A0A0G2K719	ENSRNOG00000023383	Ddx3x	1	FALSE	Predicted mitochondrial
A0A0G2K730	ENSRNOG00000022354	Pusl1	0	FALSE	Known mitochondrial
A0A0G2K747	ENSRNOG00000012100	Ssbp1	4	TRUE	Known mitochondrial
A0A0G2K756	ENSRNOG00000003185	Acbd3	0	FALSE	Known mitochondrial
A0A0G2K757	ENSRNOG00000007492	Rpn2	3	FALSE	Predicted mitochondrial
A0A0G2K761	ENSRNOG00000015292	Cul2	0	FALSE	Known mitochondrial
A0A0G2K777	ENSRNOG00000004093	Rhot1	2	TRUE	Known mitochondrial
A0A0G2K785	ENSRNOG00000034116	Gk	0	TRUE	Known mitochondrial
A0A0G2K791	ENSRNOG00000025388	Mrpl33	0	TRUE	Known mitochondrial
A0A0G2K7J6	ENSRNOG00000016560	Card19	1	TRUE	Predicted mitochondrial
A0A0G2K7K2	ENSRNOG00000006067	Aifm1	9	TRUE	Known mitochondrial
A0A0G2K7L0	ENSRNOG00000022609	Mrps10	1	TRUE	Known mitochondrial
A0A0G2K7Q7	ENSRNOG00000016833	Ide	1	TRUE	Known mitochondrial
A0A0G2K7R8	ENSRNOG00000055089	Slc44a1	0	TRUE	Known mitochondrial
A0A0G2K7Y2	ENSRNOG00000056487	Oxr1	0	TRUE	Known mitochondrial
A0A0G2K8L9	ENSRNOG00000032630	Mrps28	1	TRUE	Known mitochondrial
A0A0G2K8Q8	ENSRNOG00000059061	Uqcr10	0	TRUE	Known mitochondrial
A0A0G2K8T0	ENSRNOG00000010034	Asah1	3	FALSE	Predicted mitochondrial
A0A0G2K9B4	ENSRNOG00000008566	Mrpl15	1	TRUE	Known mitochondrial
A0A0G2K9E5	ENSRNOG00000004760	Lars2	1	TRUE	Known mitochondrial
A0A0G2K9G3	ENSRNOG00000011979	Mrps24	2	TRUE	Known mitochondrial
A0A0G2K9H8	ENSRNOG00000054775	Fkbp11	1	FALSE	Predicted mitochondrial
A0A0G2K9J0	ENSRNOG00000052840	Tanc2	0	FALSE	Predicted mitochondrial
A0A0G2K9R3	ENSRNOG00000000392	Supv3l1	3	TRUE	Known mitochondrial
A0A0G2KA12	ENSRNOG00000057626	Kif1b	0	TRUE	Known mitochondrial
A0A0G2KA23	ENSRNOG00000034116	Gk	0	TRUE	Known mitochondrial

A0A0G2KA25	ENSRNOG00000009406	Tm9sf4	1	FALSE	Predicted mitochondrial
A0A0G2KAA3	ENSRNOG00000060293	Ndufa3	2	TRUE	Known mitochondrial
A0A0G2KAE2	ENSRNOG00000013061	Tusc3	0	TRUE	Predicted mitochondrial
A0A0G2KAE6	ENSRNOG00000017513	Miga2	1	FALSE	Predicted mitochondrial
A0A0G2KAL9	ENSRNOG00000015428	Mff	2	FALSE	Known mitochondrial
A0A0G2KAM3	ENSRNOG00000007895	Pdhb	8	TRUE	Known mitochondrial
A0A0G2KAN7	ENSRNOG00000056246	Gls	3	TRUE	Known mitochondrial
A0A0G2KAP2	ENSRNOG00000021347	Mthfd2l	0	TRUE	Known mitochondrial
A0A0G2KB63	ENSRNOG00000012999	Phb2	7	TRUE	Known mitochondrial
A0A0G2QC17	ENSRNOG00000016180	Pdp1	3	FALSE	Known mitochondrial
A0A0H2UHE1	ENSRNOG00000005587	Suclg1	6	TRUE	Known mitochondrial
A0A0H2UHK3	ENSRNOG00000023923	Fastkd2	0	TRUE	Known mitochondrial
A0A0H2UHM5	ENSRNOG00000015018	Pdia3	7	FALSE	Predicted mitochondrial
A0A0H2UHS3	ENSRNOG00000020688	Ccdc51	4	FALSE	Known mitochondrial
A0A0H2UHS7	ENSRNOG00000021035	Rpl18	5	FALSE	Predicted mitochondrial
A0A0H2UHT3	ENSRNOG00000027039	Mrpl22	2	TRUE	Known mitochondrial
A0A0H2UHW2	ENSRNOG00000026691	Coq4	1	TRUE	Known mitochondrial
A0A0H2UHY0	ENSRNOG00000000833	Vars2	3	FALSE	Known mitochondrial
A0A0H2UI06	ENSRNOG00000040040	Ndufaf6	1	TRUE	Known mitochondrial
A0A0H2UI21	ENSRNOG00000018145	Crat	3	TRUE	Known mitochondrial
A0A0H2UI42	ENSRNOG00000049330	NEWGENE_1308196	2	TRUE	Known mitochondrial
A0A140TA89	ENSRNOG00000000168	Gatm	2	TRUE	Known mitochondrial
A0A140TAH5	ENSRNOG00000001709	Ap2m1	3	FALSE	Predicted mitochondrial
A0JN30	ENSRNOG00000003549	Cnpy2	1	FALSE	Predicted mitochondrial
A1L1J4	ENSRNOG00000016180	Pdp1	3	FALSE	Known mitochondrial
A1L1M0	ENSRNOG00000005257	Prkaca	0	TRUE	Known mitochondrial
A2VD14	ENSRNOG00000010609	Abcf2	0	FALSE	Predicted mitochondrial
A4F267	ENSRNOG00000003398	Tomm40l	0	TRUE	Known mitochondrial
A9UMV2	ENSRNOG00000011979	Mrps24	2	TRUE	Known mitochondrial
A9UMV7	ENSRNOG00000016952	Uqcr11	1	TRUE	Known mitochondrial
A9UMV9	ENSRNOG00000006939	Ndufa7	2	TRUE	Known mitochondrial
B0BMT9	ENSRNOG00000000172	Sqor	3	TRUE	Known mitochondrial
B0BMW2	ENSRNOG00000003049	Hsd17b10	8	TRUE	Known mitochondrial
B0BMY5	ENSRNOG00000018491	Chchd5	0	TRUE	Known mitochondrial
B0BN30	ENSRNOG00000000527	Mtch1	1	TRUE	Known mitochondrial
B0BN46	ENSRNOG00000012794	Grhpr	0	FALSE	Known mitochondrial
B0BN56	ENSRNOG00000011839	Mrps31	3	TRUE	Known mitochondrial
B0BN68	ENSRNOG00000016201	Mrps9	2	TRUE	Known mitochondrial
B0BN83	ENSRNOG00000013253	Armc1	0	FALSE	Known mitochondrial
B0BN86	ENSRNOG00000005377	Tmem11	3	TRUE	Known mitochondrial
B0BN94	ENSRNOG00000016273	Fam136a	3	FALSE	Known mitochondrial
B0BNB9	ENSRNOG00000022448	Htra2	2	TRUE	Known mitochondrial
B0BNE6	ENSRNOG00000017446	Ndufs8	3	TRUE	Known mitochondrial

B0BNE9	ENSRNOG00000047329	Mtif3	1	TRUE	Known mitochondrial
B0BNF6	ENSRNOG00000017396	5-Mar	2	TRUE	Known mitochondrial
B0BNI6	ENSRNOG00000004668	Slc25a35	1	TRUE	Known mitochondrial
B0BNJ4	ENSRNOG00000019982	Ethe1	2	TRUE	Known mitochondrial
B0BNJ9	ENSRNOG00000015437	Tmem14c	1	FALSE	Known mitochondrial
B0BNK1	ENSRNOG00000018568	Rab5c	2	FALSE	Known mitochondrial
B0BNM1	ENSRNOG00000019201	Naxe	2	TRUE	Known mitochondrial
B0K017	ENSRNOG00000010849	Adprhl2	1	TRUE	Known mitochondrial
B0K020	ENSRNOG00000000610	Cisd1	3	TRUE	Known mitochondrial
B0K031	ENSRNOG00000006992	Rpl7	4	FALSE	Predicted mitochondrial
B0K036	ENSRNOG00000037541	LOC108348111	0	TRUE	Known mitochondrial
B1H271	ENSRNOG00000020345	Slc25a42	4	TRUE	Known mitochondrial
B1WBR8	ENSRNOG00000005641	Fbxl4	0	TRUE	Known mitochondrial
B1WBW4	ENSRNOG00000012785	Armc10	2	TRUE	Known mitochondrial
B1WBY5	ENSRNOG00000008802	Dnajc11	2	TRUE	Known mitochondrial
B1WC37	ENSRNOG00000016465	Trmu	1	TRUE	Known mitochondrial
B1WC61	ENSRNOG00000014178	Acad9	3	TRUE	Known mitochondrial
B1WC67	ENSRNOG00000020470	Slc25a24	1	TRUE	Known mitochondrial
B2GUW4	ENSRNOG00000027707	Exd2	0	FALSE	Known mitochondrial
B2GUZ3	ENSRNOG00000019582	Mthfd1l	1	TRUE	Known mitochondrial
B2GUZ6	ENSRNOG00000000279	Rtn4ip1	2	TRUE	Known mitochondrial
B2GV06	ENSRNOG00000043094	Oxct1	2	TRUE	Known mitochondrial
B2GV15	ENSRNOG00000015029	Dbt	4	TRUE	Known mitochondrial
B2GV53	ENSRNOG00000004403	Slc25a32	1	TRUE	Known mitochondrial
B2GV57	ENSRNOG00000014526	Cars2	2	FALSE	Known mitochondrial
B2GV62	ENSRNOG00000018647	Mrpl20	1	TRUE	Known mitochondrial
B2GV65	ENSRNOG00000017372	Higd2a	0	FALSE	Known mitochondrial
B2GV71	ENSRNOG00000004784	Ndufaf5	1	TRUE	Known mitochondrial
B2RYM8	ENSRNOG00000004466	Fam210b	2	TRUE	Known mitochondrial
B2RYS2	ENSRNOG00000024967	Uqcrb	5	TRUE	Known mitochondrial
B2RYS8	ENSRNOG00000014078	Ndufb8	2	TRUE	Known mitochondrial
B2RYT4	ENSRNOG00000002569	Mrps14	1	TRUE	Known mitochondrial
B2RYT7	ENSRNOG00000015195	Hdhd3	3	FALSE	Known mitochondrial
B2RYU0	ENSRNOG00000026616	Ndufb2	1	TRUE	Known mitochondrial
B2RYV0	ENSRNOG00000031721	Dnajc30	0	FALSE	Known mitochondrial
B2RYW3	ENSRNOG00000009364	Ndufb9	2	TRUE	Known mitochondrial
B2RYW4	ENSRNOG00000053109	Mrpl53	2	FALSE	Known mitochondrial
B2RYW9	ENSRNOG00000013974	Fahd2a	3	FALSE	Known mitochondrial
B2RZ08	ENSRNOG00000006987	Tmem263	0	FALSE	Known mitochondrial
B2RZ79	ENSRNOG00000000701	Iscu	2	TRUE	Known mitochondrial
B2RZ89	ENSRNOG00000016949	Slc25a33	0	TRUE	Known mitochondrial
B2RZD2	ENSRNOG00000022732	Tmem126b	0	TRUE	Known mitochondrial
B2RZD4	ENSRNOG00000016387	Rpl34	0	FALSE	Predicted mitochondrial

B2RZD6	ENSRNOG00000005512	Ndufa4	2	TRUE	Known mitochondrial
B2RZD7	ENSRNOG00000037204	Lyrm9	1	FALSE	Predicted mitochondrial
B3DMA2	ENSRNOG00000010940	Acad11	1	TRUE	Known mitochondrial
B4F774	ENSRNOG00000008790	Gdap1l1	1	FALSE	Predicted mitochondrial
B4F7A1	ENSRNOG00000045961	Lyrm7	0	TRUE	Known mitochondrial
B4F7C7	ENSRNOG00000000024	Hebp1	1	FALSE	Known mitochondrial
B5DEL8	ENSRNOG00000026646	Ndufs5	3	TRUE	Known mitochondrial
B5DEN5	ENSRNOG00000024186	Eef1b2	0	FALSE	Predicted mitochondrial
B5DEP4	ENSRNOG00000042740	Mrpl42	1	FALSE	Known mitochondrial
B5DER5	ENSRNOG00000009297	Chchd1	0	TRUE	Known mitochondrial
B5DF07	ENSRNOG00000007043	RGD1305089	1	TRUE	Known mitochondrial
B5DF41	ENSRNOG00000009588	Snph	0	TRUE	Known mitochondrial
B5DFI9	ENSRNOG00000012513	Pdk3	1	TRUE	Known mitochondrial
B5DFN3	ENSRNOG00000025909	Uqcc2	0	TRUE	Known mitochondrial
C6ZII9	ENSRNOG00000013828	Sirt3	2	TRUE	Known mitochondrial
D3Z7Z4	ENSRNOG00000018464	Lipt1	0	FALSE	Known mitochondrial
D3Z900	ENSRNOG00000037850	2-Mar	4	FALSE	Known mitochondrial
D3Z946	ENSRNOG00000006608	Tmem70	1	TRUE	Known mitochondrial
D3Z9I1	ENSRNOG00000020487	Coa3	1	TRUE	Known mitochondrial
D3Z9K2	ENSRNOG00000020464	Mrpl54	0	TRUE	Known mitochondrial
D3Z9L0	ENSRNOG00000011509	Agk	2	TRUE	Known mitochondrial
D3Z9Y3	ENSRNOG00000017022	Cerk	0	FALSE	Predicted mitochondrial
D3Z9Z2	ENSRNOG00000016906	Lipt2	1	TRUE	Known mitochondrial
D3ZA85	ENSRNOG00000018410	Nfu1	2	TRUE	Known mitochondrial
D3ZA93	ENSRNOG00000018415	Acot13	0	TRUE	Predicted mitochondrial
D3ZAF6	ENSRNOG00000027049	Atp5mf	2	TRUE	Known mitochondrial
D3ZAI6	ENSRNOG00000026793	Nt5dc3	1	FALSE	Known mitochondrial
D3ZAN3	ENSRNOG00000019724	Ganab	2	FALSE	Predicted mitochondrial
D3ZAN9	ENSRNOG00000017954	Prelid3a	0	TRUE	Predicted mitochondrial
D3ZAQ0	ENSRNOG00000024066	Fundc2	1	TRUE	Known mitochondrial
D3ZAW2	ENSRNOG00000018319	Pisd	2	TRUE	Known mitochondrial
D3ZB55	ENSRNOG00000018102	Coa5	0	TRUE	Known mitochondrial
D3ZBE9	ENSRNOG00000032660	Adgrl2	1	FALSE	Predicted mitochondrial
D3ZBM3	ENSRNOG00000018053	Fech	2	TRUE	Known mitochondrial
D3ZBU7	ENSRNOG00000018366	RGD1310819	0	FALSE	Predicted mitochondrial
D3ZC63	ENSRNOG00000007690	Cmpk2	1	TRUE	Known mitochondrial
D3ZCA0	ENSRNOG00000013751	Plpbp	2	FALSE	Known mitochondrial
D3ZCG9	ENSRNOG00000004276	Itga3	1	FALSE	Known mitochondrial
D3ZCI0	ENSRNOG00000034116	Gk	0	TRUE	Known mitochondrial
D3ZCZ9	ENSRNOG00000049394	LOC679739	3	TRUE	Known mitochondrial
D3ZD09	ENSRNOG00000034161	Cox6b1	2	TRUE	Known mitochondrial
D3ZD11	ENSRNOG00000018164	Spcs2	2	FALSE	Predicted mitochondrial
D3ZD23	ENSRNOG00000018345	Abce1	0	TRUE	Predicted mitochondrial

D3ZDE4	ENSRNOG00000011617	Dguok	2	TRUE	Known mitochondrial
D3ZDP2	ENSRNOG00000032780	Mrpl58	1	TRUE	Known mitochondrial
D3ZDX7	ENSRNOG00000018042	Mrpl48	1	TRUE	Known mitochondrial
D3ZEG8	ENSRNOG00000009255	Timm29	2	TRUE	Predicted mitochondrial
D3ZEH2	ENSRNOG00000060379	Foxred1	2	TRUE	Known mitochondrial
D3ZEJ2	ENSRNOG00000017988	Mcur1	1	TRUE	Known mitochondrial
D3ZF03	ENSRNOG00000037957	Aifm3	0	TRUE	Known mitochondrial
D3ZF13	ENSRNOG00000018129	Ndufab1	2	TRUE	Known mitochondrial
D3ZFJ6	ENSRNOG00000018081	Lactb	2	TRUE	Known mitochondrial
D3ZFQ3	ENSRNOG00000027444	Nubpl	0	TRUE	Known mitochondrial
D3ZFQ8	ENSRNOG00000012457	Cyc1	2	TRUE	Known mitochondrial
D3ZFR9	ENSRNOG00000027919	Rdh13	1	FALSE	Predicted mitochondrial
D3ZFS7	ENSRNOG00000023130	Lyplal1	0	FALSE	Predicted mitochondrial
D3ZG43	ENSRNOG00000009155	Ndufs3	7	TRUE	Known mitochondrial
D3ZG78	ENSRNOG00000024535	Zzef1	0	FALSE	Predicted mitochondrial
D3ZG95	ENSRNOG00000012767	Micu3	1	FALSE	Known mitochondrial
D3ZGM1	ENSRNOG00000009484	Ptcd3	2	TRUE	Known mitochondrial
D3ZGQ2	ENSRNOG00000012853	Tk2	0	TRUE	Known mitochondrial
D3ZHD3	ENSRNOG00000050169	Pet100	0	TRUE	Known mitochondrial
D3ZHF8	ENSRNOG00000002207	Guf1	1	TRUE	Known mitochondrial
D3ZIC4	ENSRNOG00000051440	Ppp1r12b	0	FALSE	Predicted mitochondrial
D3ZIE9	ENSRNOG00000015267	LOC108348083	1	TRUE	Known mitochondrial
D3ZII8	ENSRNOG00000015589	Smyd5	0	FALSE	Predicted mitochondrial
D3ZIN7	ENSRNOG00000010363	Mrps23	2	TRUE	Known mitochondrial
D3ZIS5	ENSRNOG00000050052	Cox19	0	TRUE	Known mitochondrial
D3ZJ86	ENSRNOG00000000879	Slc9a6	0	FALSE	Known mitochondrial
D3ZJG4	ENSRNOG00000014744	Pacs2	0	TRUE	Known mitochondrial
D3ZJH9	ENSRNOG00000015582	Me2	1	TRUE	Known mitochondrial
D3ZJX5	ENSRNOG00000037638	Timm50	1	TRUE	Known mitochondrial
D3ZJY1	ENSRNOG00000042720	Mrpl28	1	TRUE	Known mitochondrial
D3ZKG1	ENSRNOG00000050843	Mut	2	FALSE	Known mitochondrial
D3ZKT0	ENSRNOG00000007874	Tamm41	1	TRUE	Known mitochondrial
D3ZL85	ENSRNOG00000025910	Hccs	2	TRUE	Known mitochondrial
D3ZLF0	ENSRNOG00000048168	Gtpbp6	0	FALSE	Known mitochondrial
D3ZLK9	ENSRNOG00000015021	Naxd	2	TRUE	Known mitochondrial
D3ZLT1	ENSRNOG00000028717	Ndufb7	2	TRUE	Known mitochondrial
D3ZLT7	ENSRNOG00000007968	RGD1305350	0	FALSE	Predicted mitochondrial
D3ZLU4	ENSRNOG00000016465	Trmu	1	TRUE	Known mitochondrial
D3ZM09	ENSRNOG00000019962	Sars2	2	TRUE	Known mitochondrial
D3ZM21	ENSRNOG00000013968	Comtd1	2	FALSE	Known mitochondrial
D3ZML4	ENSRNOG00000029095	Trabd	1	FALSE	Known mitochondrial
D3ZMR2	ENSRNOG00000000495	Uhrf1bp1	0	FALSE	Predicted mitochondrial
D3ZMR9	ENSRNOG00000013845	Mrpl21	0	TRUE	Known mitochondrial

D3ZNG0	ENSRNOG00000032660	Adgrl2	1	FALSE	Predicted mitochondrial
D3ZNK1	ENSRNOG00000023837	Mtx3	1	TRUE	Known mitochondrial
D3ZNY3	ENSRNOG00000011618	Mmaa	1	FALSE	Known mitochondrial
D3ZP13	ENSRNOG00000018574	Qsox2	0	FALSE	Predicted mitochondrial
D3ZP87	ENSRNOG00000016338	Fam92a	0	FALSE	Predicted mitochondrial
D3ZPE6	ENSRNOG00000019165	Mrpl51	0	TRUE	Known mitochondrial
D3ZPF2	ENSRNOG00000010539	Mcat	2	TRUE	Known mitochondrial
D3ZPG5	ENSRNOG00000028556	Usp30	1	TRUE	Known mitochondrial
D3ZPN5	ENSRNOG00000016578	Mtpap	1	FALSE	Known mitochondrial
D3ZPP9	ENSRNOG00000016560	Card19	1	TRUE	Predicted mitochondrial
D3ZPR0	ENSRNOG00000007665	Cse1l	1	FALSE	Predicted mitochondrial
D3ZQB6	ENSRNOG00000011338	Hdhd5	2	TRUE	Known mitochondrial
D3ZQD3	ENSRNOG00000019955	Ogdhl	1	TRUE	Known mitochondrial
D3ZQG0	ENSRNOG00000037238	Mrm3	1	TRUE	Known mitochondrial
D3ZQM3	ENSRNOG00000004276	Itga3	1	FALSE	Known mitochondrial
D3ZQX3	ENSRNOG00000019949	Mrps12	1	TRUE	Known mitochondrial
D3ZRC4	ENSRNOG00000039091	Pnpla8	1	FALSE	Known mitochondrial
D3ZRH1	ENSRNOG00000009713	Oxa1l	1	TRUE	Known mitochondrial
D3ZRJ0	ENSRNOG00000012685	Adck1	1	FALSE	Predicted mitochondrial
D3ZS58	ENSRNOG00000017571	Ndufa2	2	TRUE	Known mitochondrial
D3ZSA9	ENSRNOG00000021118	Nomo1	1	FALSE	Predicted mitochondrial
D3ZSD8	ENSRNOG00000021096	Tmem143	0	TRUE	Predicted mitochondrial
D3ZSN7	ENSRNOG00000025269	Slc25a44	0	TRUE	Known mitochondrial
D3ZT71	ENSRNOG00000012394	Bcl2l13	1	TRUE	Known mitochondrial
D3ZT90	ENSRNOG00000003307	Gcdh	2	TRUE	Known mitochondrial
D3ZT98	ENSRNOG00000021866	Bola3	2	TRUE	Known mitochondrial
D3ZTG3	ENSRNOG00000004783	Fam171b	0	FALSE	Predicted mitochondrial
D3ZTN2	ENSRNOG00000010149	Cmc1	1	TRUE	Known mitochondrial
D3ZTR1	ENSRNOG00000000926	Mrps17	0	TRUE	Known mitochondrial
D3ZTR5	ENSRNOG00000000918	Zbed5	0	FALSE	Predicted mitochondrial
D3ZTW7	ENSRNOG00000003719	Atpaf2	1	FALSE	Known mitochondrial
D3ZTW8	ENSRNOG00000003724	Mrpl27	2	TRUE	Known mitochondrial
D3ZTW9	ENSRNOG00000014801	Exog	2	TRUE	Known mitochondrial
D3ZUA0	ENSRNOG00000021347	Mthfd2l	0	TRUE	Known mitochondrial
D3ZUF9	ENSRNOG00000016511	Pitrm1	2	TRUE	Known mitochondrial
D3ZUI9	ENSRNOG00000042653	Ndufaf8	0	TRUE	Predicted mitochondrial
D3ZUJ5	ENSRNOG00000018904	Dtymk	0	TRUE	Predicted mitochondrial
D3ZUJ8	ENSRNOG00000006749	Tmtc3	0	FALSE	Predicted mitochondrial
D3ZUM2	ENSRNOG00000010244	Sarm1	1	TRUE	Known mitochondrial
D3ZUX5	ENSRNOG00000013211	Chchd3	1	TRUE	Known mitochondrial
D3ZUX7	ENSRNOG00000015077	Acsf3	1	TRUE	Known mitochondrial
D3ZUY0	ENSRNOG00000039551	Rdh14	1	FALSE	Predicted mitochondrial
D3ZVN7	ENSRNOG00000003567	Ppox	2	TRUE	Known mitochondrial

D3ZVS2	ENSRNOG00000004857	L2hgdh	2	TRUE	Known mitochondrial
D3ZVU9	ENSRNOG00000049351	Nat8l	0	TRUE	Known mitochondrial
D3ZWB7	ENSRNOG00000019031	Neu4	0	FALSE	Known mitochondrial
D3ZWF3	ENSRNOG00000004829	Immp1l	0	TRUE	Known mitochondrial
D3ZWW5	ENSRNOG00000002246	Slc30a9	1	FALSE	Known mitochondrial
D3ZX69	ENSRNOG00000009567	Mrpl10	3	TRUE	Known mitochondrial
D3ZX74	ENSRNOG00000029571	Coq10a	0	TRUE	Known mitochondrial
D3ZXA6	ENSRNOG00000022593	Pdpr	1	TRUE	Known mitochondrial
D3ZXF8	ENSRNOG00000014890	Mrpl43	1	TRUE	Known mitochondrial
D3ZXF9	ENSRNOG00000036695	Mrpl12	2	TRUE	Known mitochondrial
D3ZXI0	ENSRNOG00000036682	Pycr1	1	TRUE	Known mitochondrial
D3ZXK4	ENSRNOG00000018910	Abhd11	2	FALSE	Known mitochondrial
D3ZXQ6	ENSRNOG00000026180	Letm2	0	TRUE	Known mitochondrial
D3ZY44	ENSRNOG00000010164	Mrps2	1	TRUE	Known mitochondrial
D3ZY50	ENSRNOG00000010169	Atpaf1	0	TRUE	Known mitochondrial
D3ZY71	ENSRNOG00000047812	Triap1	0	TRUE	Known mitochondrial
D3ZYL4	ENSRNOG00000053615	Mrpl50	2	TRUE	Known mitochondrial
D3ZYM7	ENSRNOG00000047970	Lyrm4	0	FALSE	Known mitochondrial
D3ZYT2	ENSRNOG00000015192	Mrps5	1	TRUE	Known mitochondrial
D3ZYU4	ENSRNOG00000004553	Cox20	0	TRUE	Known mitochondrial
D3ZYY0	ENSRNOG00000020544	Sdr39u1	2	FALSE	Predicted mitochondrial
D3ZZ21	ENSRNOG00000024539	Ndufb6	2	TRUE	Known mitochondrial
D3ZZ32	ENSRNOG00000007326	Prepl	2	FALSE	Known mitochondrial
D3ZZN3	ENSRNOG00000007102	Acss1	0	TRUE	Known mitochondrial
D3ZZR9	ENSRNOG00000021153	Fkbp2	2	FALSE	Predicted mitochondrial
D3ZZU4	ENSRNOG00000015304	Tmem160	2	FALSE	Predicted mitochondrial
D3ZZV1	ENSRNOG00000004608	Pam16	1	TRUE	Known mitochondrial
D3ZZX9	ENSRNOG00000036572	Sfxn4	0	FALSE	Known mitochondrial
D4A040	ENSRNOG00000018531	Mrps11	2	TRUE	Known mitochondrial
D4A099	ENSRNOG00000012020	Nsun4	1	TRUE	Known mitochondrial
D4A0T0	ENSRNOG00000014568	Ndufb10	3	TRUE	Known mitochondrial
D4A0T8	ENSRNOG00000005589	Dhrs7	0	FALSE	Predicted mitochondrial
D4A0Y4	ENSRNOG00000019760	Oxnad1	2	TRUE	Known mitochondrial
D4A104	ENSRNOG00000010540	Mrpl45	1	TRUE	Known mitochondrial
D4A131	ENSRNOG00000020659	Mrpl4	2	TRUE	Known mitochondrial
D4A133	ENSRNOG00000001992	Atp6v1a	4	FALSE	Predicted mitochondrial
D4A197	ENSRNOG00000016327	Mcee	2	FALSE	Known mitochondrial
D4A1G1	ENSRNOG00000042419	Acyp2	1	FALSE	Predicted mitochondrial
D4A1H7	ENSRNOG00000016010	Mul1	0	TRUE	Known mitochondrial
D4A1T7	ENSRNOG00000019684	Fam173a	1	FALSE	Predicted mitochondrial
D4A264	ENSRNOG00000016239	Zadh2	2	FALSE	Predicted mitochondrial
D4A280	ENSRNOG00000005509	Pak7	0	TRUE	Known mitochondrial
D4A305	ENSRNOG00000031653	Ccdc58	1	TRUE	Known mitochondrial

D4A3E8	ENSRNOG00000017272	Mrps27	2	TRUE	Known mitochondrial
D4A3V2	ENSRNOG00000008569	Ndufa6	2	TRUE	Known mitochondrial
D4A414	ENSRNOG00000017230	Cox15	2	TRUE	Known mitochondrial
D4A471	ENSRNOG00000011279	Cmc2	0	TRUE	Known mitochondrial
D4A4A9	ENSRNOG00000006968	Mrpl19	1	TRUE	Known mitochondrial
D4A4P3	ENSRNOG00000011825	Ndufb3	1	TRUE	Known mitochondrial
D4A4P4	ENSRNOG00000020642	Flad1	2	FALSE	Known mitochondrial
D4A4Q4	ENSRNOG00000002196	Ociad2	1	FALSE	Known mitochondrial
D4A4V1	ENSRNOG00000002178	Mrps18c	0	TRUE	Known mitochondrial
D4A4W6	ENSRNOG00000012314	Slirp	2	TRUE	Known mitochondrial
D4A4W7	ENSRNOG00000011923	Mgarp	0	TRUE	Known mitochondrial
D4A520	ENSRNOG00000021440	Pptc7	1	FALSE	Known mitochondrial
D4A565	ENSRNOG00000011949	Ndufb5	5	TRUE	Known mitochondrial
D4A568	ENSRNOG00000003052	Cox18	0	FALSE	Known mitochondrial
D4A5J2	ENSRNOG00000003332	Nt5m	0	FALSE	Known mitochondrial
D4A5Q9	ENSRNOG00000011599	Gldc	1	TRUE	Known mitochondrial
D4A5W8	ENSRNOG00000002949	Pgs1	1	TRUE	Known mitochondrial
D4A5X7	ENSRNOG00000005850	Gdap1	1	TRUE	Known mitochondrial
D4A601	ENSRNOG00000008405	Taco1	1	TRUE	Known mitochondrial
D4A634	ENSRNOG00000053859	Ranbp6	1	FALSE	Known mitochondrial
D4A6D7	ENSRNOG00000002977	Ttc19	1	TRUE	Known mitochondrial
D4A6Y6	ENSRNOG00000012831	Slc25a26	2	TRUE	Known mitochondrial
D4A742	ENSRNOG00000045555	Romo1	0	FALSE	Known mitochondrial
D4A746	ENSRNOG00000037229	Gmppb	0	FALSE	Predicted mitochondrial
D4A7F2	ENSRNOG00000017166	Mycbp	0	FALSE	Known mitochondrial
D4A7L4	ENSRNOG00000008329	Ndufb11	2	FALSE	Known mitochondrial
D4A7N1	ENSRNOG00000060248	Chchd6	2	TRUE	Known mitochondrial
D4A7X1	ENSRNOG00000006898	Mrps16	2	TRUE	Known mitochondrial
D4A7X5	ENSRNOG00000006893	Ppm1k	0	TRUE	Known mitochondrial
D4A7Y9	ENSRNOG00000008461	Smdt1	1	TRUE	Known mitochondrial
D4A830	ENSRNOG00000012091	Ppa2	1	FALSE	Known mitochondrial
D4A833	ENSRNOG00000012136	Mrps30	2	TRUE	Known mitochondrial
D4A899	ENSRNOG00000025539	Vps13a	0	FALSE	Known mitochondrial
D4A8N2	ENSRNOG00000023020	Fdx1l	1	FALSE	Known mitochondrial
D4A929	ENSRNOG00000003243	Wdr81	0	TRUE	Known mitochondrial
D4A9H7	ENSRNOG00000016167	Spata2L	0	FALSE	Predicted mitochondrial
D4A9I4	ENSRNOG00000028403	Ptcd2	1	TRUE	Known mitochondrial
D4A9M6	ENSRNOG00000002373	Akap1	2	TRUE	Known mitochondrial
D4A9V7	ENSRNOG00000050002	Mars2	1	TRUE	Known mitochondrial
D4A9Z6	ENSRNOG00000001842	Mrps35	2	TRUE	Known mitochondrial
D4AAE9	ENSRNOG00000048258	Cisd2	0	TRUE	Known mitochondrial
D4AB01	ENSRNOG00000015866	Hint2	2	TRUE	Known mitochondrial
D4ABI7	ENSRNOG00000030232	Hacd3	1	TRUE	Predicted mitochondrial

D4ABL7	ENSRNOG00000025388	Mrpl33	0	TRUE	Known mitochondrial
D4ABM5	ENSRNOG00000015479	Mrps34	2	TRUE	Known mitochondrial
D4AC23	ENSRNOG00000015630	Cct7	0	FALSE	Predicted mitochondrial
D4AC65	ENSRNOG00000010636	Coa7	0	TRUE	Known mitochondrial
D4AC73	ENSRNOG00000031769	Chchd7	0	FALSE	Predicted mitochondrial
D4ACA5	ENSRNOG00000025156	LOC100911130	2	TRUE	Known mitochondrial
D4ACE9	ENSRNOG00000039494	Aass	2	TRUE	Known mitochondrial
D4ACG2	ENSRNOG00000028512	Ilvbl	1	FALSE	Predicted mitochondrial
D4ACN8	ENSRNOG00000015932	Plgrkt	1	FALSE	Known mitochondrial
D4ACN9	ENSRNOG00000013802	Slc25a36	1	TRUE	Known mitochondrial
D4ACP2	ENSRNOG00000014352	Smim12	1	FALSE	Predicted mitochondrial
D4ACP8	ENSRNOG00000017889	Serac1	0	FALSE	Known mitochondrial
D4ACY1	ENSRNOG00000029415	RGD1565784	1	FALSE	Known mitochondrial
D4ADG2	ENSRNOG00000022725	Iba57	2	TRUE	Known mitochondrial
D4ADS4	ENSRNOG00000004245	Mgst3	2	FALSE	Known mitochondrial
D4AE56	ENSRNOG00000014050	Ptges2	2	TRUE	Known mitochondrial
D4AE90	ENSRNOG00000001483	Rcc1l	1	FALSE	Known mitochondrial
D4AEG6	ENSRNOG00000015989	Mrpl32	1	TRUE	Known mitochondrial
E9PSS2	ENSRNOG00000047816	Ccs	0	FALSE	Known mitochondrial
E9PT51	ENSRNOG00000009252	Poldip2	2	TRUE	Known mitochondrial
E9PT90	ENSRNOG00000014155	Spart	0	TRUE	Known mitochondrial
E9PTB3	ENSRNOG00000018860	Mtg1	0	TRUE	Known mitochondrial
E9PTK4	ENSRNOG00000033076	Thnsl1	1	FALSE	Known mitochondrial
E9PTV0	ENSRNOG00000002928	Guk1	1	FALSE	Known mitochondrial
E9PU34	ENSRNOG00000019501	Rmnd1	2	FALSE	Known mitochondrial
F1LLZ7	ENSRNOG00000003063	Phka1	1	FALSE	Predicted mitochondrial
F1LM47	ENSRNOG00000017481	Sucla2	2	TRUE	Known mitochondrial
F1LMJ8	ENSRNOG00000011168	Micu2	0	TRUE	Known mitochondrial
F1LMM8	ENSRNOG00000004172	Pdk2	2	TRUE	Known mitochondrial
F1LMZ4	ENSRNOG00000025285	Gfm2	4	TRUE	Known mitochondrial
F1LN92	ENSRNOG00000017965	Afg3l2	2	TRUE	Known mitochondrial
F1LNB3	ENSRNOG00000002899	Akap10	0	TRUE	Known mitochondrial
F1LNF7	ENSRNOG00000010277	Idh3a	5	TRUE	Known mitochondrial
F1LNG8	ENSRNOG00000049430	Mpv17	2	TRUE	Known mitochondrial
F1LNT0	ENSRNOG00000027582	Dpysl4	0	FALSE	Predicted mitochondrial
F1LNV5	ENSRNOG00000043436	Micu1	4	TRUE	Known mitochondrial
F1LP30	ENSRNOG00000013293	Mccc1	3	TRUE	Known mitochondrial
F1LP46	ENSRNOG00000000392	Supv3l1	3	TRUE	Known mitochondrial
F1LP77	ENSRNOG00000026180	Letm2	0	TRUE	Known mitochondrial
F1LPB3	ENSRNOG00000016265	Acsl5	3	TRUE	Known mitochondrial
F1LPD6	ENSRNOG00000032908	Acaa1a	6	FALSE	Predicted mitochondrial
F1LPG5	ENSRNOG00000002721	Ndufb4	2	TRUE	Known mitochondrial
F1LPV8	ENSRNOG00000005686	Suclg2	3	TRUE	Known mitochondrial

F1LQ14	ENSRNOG00000016387	Rpl34	0	FALSE	Predicted mitochondrial
F1LQI1	ENSRNOG00000014743	Hagh	2	FALSE	Known mitochondrial
F1LQL9	ENSRNOG00000007377	Slit3	0	FALSE	Known mitochondrial
F1LQZ0	ENSRNOG00000008934	Tmem65	0	TRUE	Known mitochondrial
F1LRK4	ENSRNOG00000003392	Grsf1	2	TRUE	Known mitochondrial
F1LRV6	ENSRNOG00000017250	Gmpr	0	FALSE	Predicted mitochondrial
F1LS98	ENSRNOG00000003491	Prkca	0	FALSE	Predicted mitochondrial
F1LSM2	ENSRNOG00000012287	Slc35e1	1	FALSE	Predicted mitochondrial
F1LT13	ENSRNOG00000003795	Angel2	1	FALSE	Known mitochondrial
F1LU71	ENSRNOG00000011684	Auh	2	TRUE	Known mitochondrial
F1LUC0	ENSRNOG00000004200	Sybu	0	FALSE	Known mitochondrial
F1LVS1	ENSRNOG00000017893	Baiap3	0	FALSE	Predicted mitochondrial
F1LVY9	ENSRNOG00000022303	Pdf	0	TRUE	Known mitochondrial
F1LWG4	ENSRNOG00000005006	Ndufaf1	2	FALSE	Known mitochondrial
F1LWN5	ENSRNOG00000001073	RGD1563482	1	TRUE	Known mitochondrial
F1LXA0	ENSRNOG00000007407	Ndufa12	3	TRUE	Known mitochondrial
F1LYH3	ENSRNOG00000037659	Mto1	1	TRUE	Known mitochondrial
F1LYJ8	ENSRNOG00000016968	Gramd4	0	TRUE	Known mitochondrial
F1LZH9	ENSRNOG00000021384	Ankrd44	0	FALSE	Predicted mitochondrial
F1LZW6	ENSRNOG00000009957	Slc25a13	2	TRUE	Known mitochondrial
F1M038	ENSRNOG00000038190	Dok6	0	FALSE	Predicted mitochondrial
F1M1E4	ENSRNOG00000014806	Pnkd	1	TRUE	Known mitochondrial
F1M2P8	ENSRNOG00000003491	Prkca	0	FALSE	Predicted mitochondrial
F1M3T8	ENSRNOG00000017406	Atrnl1	0	FALSE	Predicted mitochondrial
F1M4J0	ENSRNOG00000011341	Rictor	0	FALSE	Predicted mitochondrial
F1M5N4	ENSRNOG00000017311	Me3	0	TRUE	Known mitochondrial
F1M6D0	ENSRNOG00000059381	Mrps6	1	TRUE	Known mitochondrial
F1M6X5	ENSRNOG00000001890	Txnrd2	4	TRUE	Known mitochondrial
F1M779	ENSRNOG00000004291	Cltc	5	FALSE	Predicted mitochondrial
F1M7T0	ENSRNOG00000032660	Adgrl2	1	FALSE	Predicted mitochondrial
F1M853	ENSRNOG00000005958	Rrbp1	1	FALSE	Predicted mitochondrial
F1M8H2	ENSRNOG00000019508	Wars2	1	TRUE	Known mitochondrial
F1M8Y4	ENSRNOG00000004328	Deptor	0	FALSE	Predicted mitochondrial
F1M953	ENSRNOG00000019525	Hspa9	8	TRUE	Known mitochondrial
F1M9C9	ENSRNOG00000016087	Hars2	1	TRUE	Known mitochondrial
F1MAF8	ENSRNOG00000004519	Atg2b	0	FALSE	Predicted mitochondrial
F1MAR6	ENSRNOG00000000281	Prodh1	1	TRUE	Known mitochondrial
F6Q5K7	ENSRNOG00000000804	Mrps18b	1	TRUE	Known mitochondrial
F7EPE0	ENSRNOG00000000571	Psap	4	FALSE	Predicted mitochondrial
F7EV94	ENSRNOG00000020607	Bckdha	5	TRUE	Known mitochondrial
F7EZ89	ENSRNOG00000003889	Tbc1d15	0	FALSE	Known mitochondrial
F7EZZ0	ENSRNOG00000020373	Dap3	2	TRUE	Known mitochondrial
F7F172	ENSRNOG00000004926	Mettl15	0	FALSE	Known mitochondrial

F7F557	ENSRNOG00000015187	Acot8	0	FALSE	Predicted mitochondrial
F7F588	ENSRNOG00000013585	Nmnat3	2	TRUE	Known mitochondrial
F7FFR1	ENSRNOG00000008643	Rars2	1	TRUE	Known mitochondrial
F7FHF7	ENSRNOG00000046447	LOC100911485	0	TRUE	Known mitochondrial
F8WFK6	ENSRNOG00000013604	Gpx4	1	TRUE	Known mitochondrial
F8WFW5	ENSRNOG00000019560	Pde2a	0	TRUE	Known mitochondrial
F8WG67	ENSRNOG00000010580	Acot7	2	FALSE	Known mitochondrial
G3V628	ENSRNOG00000000812	RGD1302996	1	FALSE	Predicted mitochondrial
G3V640	ENSRNOG00000001058	Timm44	4	TRUE	Known mitochondrial
G3V644	ENSRNOG00000001182	Ndufv3	2	TRUE	Known mitochondrial
G3V698	ENSRNOG00000002194	Coq2	0	TRUE	Known mitochondrial
G3V6D3	ENSRNOG00000002840	Atp5f1b	14	TRUE	Known mitochondrial
G3V6G7	ENSRNOG00000003600	Pnpt1	2	TRUE	Known mitochondrial
G3V6H0	ENSRNOG00000050510	LOC100363782	2	FALSE	Predicted mitochondrial
G3V6H5	ENSRNOG00000003815	Slc25a11	4	FALSE	Known mitochondrial
G3V6P2	ENSRNOG00000005061	Dlst	6	TRUE	Known mitochondrial
G3V6P7	ENSRNOG00000004860	Myh9	4	FALSE	Known mitochondrial
G3V6R5	ENSRNOG00000005987	Suox	6	FALSE	Known mitochondrial
G3V6R7	ENSRNOG00000005993	Oxsm	3	TRUE	Known mitochondrial
G3V6S3	ENSRNOG00000006197	Calu	2	FALSE	Predicted mitochondrial
G3V6S5	ENSRNOG00000005602	Mthfd1	3	FALSE	Predicted mitochondrial
G3V728	ENSRNOG00000008374	Nipsnap1	4	TRUE	Known mitochondrial
G3V734	ENSRNOG00000008236	Decr1	6	TRUE	Known mitochondrial
G3V741	ENSRNOG00000008289	Slc25a3	8	TRUE	Known mitochondrial
G3V743	ENSRNOG00000008648	Mogs	4	FALSE	Predicted mitochondrial
G3V7B6	ENSRNOG00000009723	Ptpmt1	2	FALSE	Known mitochondrial
G3V7H4	ENSRNOG00000011171	Rims3	0	FALSE	Predicted mitochondrial
G3V7I0	ENSRNOG00000010958	Prdx3	6	TRUE	Known mitochondrial
G3V7I3	ENSRNOG00000010776	Atp13a1	1	FALSE	Predicted mitochondrial
G3V7I5	ENSRNOG00000011497	Aldh1b1	3	TRUE	Known mitochondrial
G3V7J0	ENSRNOG00000011419	Aldh6a1	8	TRUE	Known mitochondrial
G3V7K6	ENSRNOG00000012100	Ssbp1	4	TRUE	Known mitochondrial
G3V7P3	ENSRNOG00000012650	Mrpl3	2	TRUE	Known mitochondrial
G3V7Q3	ENSRNOG00000012775	Ccdc127	1	FALSE	Known mitochondrial
G3V7Y3	ENSRNOG00000014625	Atp5f1d	3	TRUE	Known mitochondrial
G3V7Z3	ENSRNOG00000015588	Nol3	0	TRUE	Known mitochondrial
G3V827	ENSRNOG00000016097	Kyat1	3	FALSE	Predicted mitochondrial
G3V879	ENSRNOG00000017012	Coq7	3	TRUE	Known mitochondrial
G3V886	ENSRNOG00000017100	LOC108348078	3	FALSE	Known mitochondrial
G3V888	ENSRNOG00000016924	Acly	3	FALSE	Known mitochondrial
G3V8C3	ENSRNOG00000018087	Vim	2	FALSE	Predicted mitochondrial
G3V8C4	ENSRNOG00000018109	Clic4	1	TRUE	Known mitochondrial
G3V8F5	ENSRNOG00000018556	Tomm40	4	TRUE	Known mitochondrial

G3V8F9	ENSRNOG00000018662	Amacr	3	TRUE	Known mitochondrial
G3V8M4	ENSRNOG00000019851	Cox6a2	0	TRUE	Known mitochondrial
G3V8R1	ENSRNOG00000020456	Nucb2	3	FALSE	Known mitochondrial
G3V8T5	ENSRNOG00000020793	Ruvbl2	0	FALSE	Known mitochondrial
G3V8T7	ENSRNOG00000020860	Tdrkh	0	TRUE	Known mitochondrial
G3V8T9	ENSRNOG00000020876	Bax	2	TRUE	Known mitochondrial
G3V8U8	ENSRNOG00000020956	Bcat2	3	TRUE	Known mitochondrial
G3V8V0	ENSRNOG00000021010	Arl2	1	FALSE	Known mitochondrial
G3V8V6	ENSRNOG00000021174	Macrod1	4	FALSE	Known mitochondrial
G3V8Z7	ENSRNOG00000023035	Smim8	0	FALSE	Known mitochondrial
G3V913	ENSRNOG00000023546	Hspb1	0	FALSE	Known mitochondrial
G3V915	ENSRNOG00000027017	Rnasel	0	FALSE	Known mitochondrial
G3V936	ENSRNOG00000023520	Cs	8	TRUE	Known mitochondrial
G3V945	ENSRNOG00000023538	Aldh5a1	5	TRUE	Known mitochondrial
G3V965	ENSRNOG00000028623	Agpat5	1	TRUE	Known mitochondrial
G3V985	ENSRNOG00000028699	Sco1	1	TRUE	Known mitochondrial
G3V996	ENSRNOG00000029855	Letmd1	2	TRUE	Predicted mitochondrial
G3V9G4	ENSRNOG00000016924	Acly	3	FALSE	Known mitochondrial
G3V9I7	ENSRNOG00000030334	Adck5	1	FALSE	Predicted mitochondrial
G3V9N1	ENSRNOG00000037443	Pgam5	2	TRUE	Known mitochondrial
G3V9N6	ENSRNOG00000020616	LOC103689986	1	FALSE	Predicted mitochondrial
G3V9S0	ENSRNOG00000003973	Cyb5r1	4	FALSE	Predicted mitochondrial
G3V9S6	ENSRNOG00000040005	LOC108348144	1	FALSE	Known mitochondrial
G3V9U2	ENSRNOG00000013766	Acaa2	10	TRUE	Known mitochondrial
G3V9W4	ENSRNOG00000019196	Xpnpep3	2	TRUE	Known mitochondrial
G3V9Y1	ENSRNOG00000002886	Myh10	2	FALSE	Known mitochondrial
M0R4L6	ENSRNOG00000037655	Gatb	1	TRUE	Known mitochondrial
M0R4V4	ENSRNOG00000047781	Slc25a23	1	TRUE	Known mitochondrial
M0R589	ENSRNOG00000023271	Parl	1	TRUE	Known mitochondrial
M0R6J0	ENSRNOG00000047563	Mrpl39	1	TRUE	Known mitochondrial
M0R776	ENSRNOG00000061213	Mrps36	1	TRUE	Known mitochondrial
M0R785	ENSRNOG00000051180	LOC100911516	0	TRUE	Known mitochondrial
M0R7G4	ENSRNOG00000046918	Apoo	1	TRUE	Known mitochondrial
M0R7R2	ENSRNOG00000047118	LOC683897	0	FALSE	Predicted mitochondrial
M0R7T2	ENSRNOG00000047089	Alkbh7	1	TRUE	Known mitochondrial
M0R7V3	ENSRNOG00000046918	Apoo	1	TRUE	Known mitochondrial
M0R8V0	ENSRNOG00000050317	Uhrf1bp1l	0	FALSE	Predicted mitochondrial
M0R959	ENSRNOG00000049426	Mmab	0	FALSE	Known mitochondrial
M0R9I6	ENSRNOG00000050214	Amt	2	TRUE	Known mitochondrial
M0RAD5	ENSRNOG00000047052	Clpp	2	TRUE	Known mitochondrial
M0RAI4	ENSRNOG00000025353	Ears2	2	TRUE	Known mitochondrial
M0RAK4	ENSRNOG00000015213	Fxn	2	TRUE	Known mitochondrial
M0RAM5	ENSRNOG00000048812	Gpx1	6	TRUE	Known mitochondrial

M0RCP9	ENSRNOG00000050051	Pin4	0	FALSE	Known mitochondrial
M0RCX0	ENSRNOG00000047796	Pcbd2	0	FALSE	Predicted mitochondrial
M0RDC8	ENSRNOG00000047106	Mtrf1	0	FALSE	Known mitochondrial
M0RDI5	ENSRNOG00000045920	Mcu	1	TRUE	Known mitochondrial
M0RDJ4	ENSRNOG00000047250	Gmfb	1	FALSE	Predicted mitochondrial
M0RDK9	ENSRNOG00000048164	Acad8	0	FALSE	Known mitochondrial
M0RDY2	ENSRNOG00000048371	AABR07059232.1	1	FALSE	Known mitochondrial
M0RE03	ENSRNOG00000052096	Cox11	0	TRUE	Known mitochondrial
O35077	ENSRNOG00000056457	Gpd1	4	FALSE	Predicted mitochondrial
O35092	ENSRNOG00000007040	Timm17a	2	TRUE	Known mitochondrial
O35244	ENSRNOG00000002896	Prdx6	3	FALSE	Predicted mitochondrial
O35303	ENSRNOG00000001813	Dnm1l	1	TRUE	Known mitochondrial
O35509	ENSRNOG00000007648	Rab11b	1	FALSE	Known mitochondrial
O35547	ENSRNOG00000019180	Acsl4	1	TRUE	Known mitochondrial
O35567	ENSRNOG00000015511	Atic	2	FALSE	Predicted mitochondrial
O35796	ENSRNOG00000006949	C1qbp	4	TRUE	Known mitochondrial
O70595	ENSRNOG00000018697	Abcb6	2	TRUE	Known mitochondrial
O88658	ENSRNOG00000057626	Kif1b	0	TRUE	Known mitochondrial
O88767	ENSRNOG00000018289	Park7	5	TRUE	Known mitochondrial
O88794	ENSRNOG00000046493	Pnpo	0	FALSE	Known mitochondrial
O89035	ENSRNOG00000036693	Slc25a10	5	TRUE	Known mitochondrial
P00173	ENSRNOG00000015205	Cyb5a	6	FALSE	Predicted mitochondrial
P00388	ENSRNOG00000001442	Por	4	TRUE	Predicted mitochondrial
P00406	ENSRNOG00000030371	Mt-co2	5	TRUE	Known mitochondrial
P00507	ENSRNOG00000011782	Got2	9	TRUE	Known mitochondrial
P03889	ENSRNOG00000030644	Mt-nd1	3	TRUE	Known mitochondrial
P04166	ENSRNOG00000011142	Cyb5b	5	TRUE	Known mitochondrial
P04182	ENSRNOG00000016807	Oat	6	TRUE	Known mitochondrial
P04636	ENSRNOG00000001440	Mdh2	13	TRUE	Known mitochondrial
P04644	ENSRNOG00000045885	LOC100365810	1	FALSE	Predicted mitochondrial
P04646	ENSRNOG00000031641	LOC108351058	1	FALSE	Predicted mitochondrial
P04764	ENSRNOG00000017895	Eno1	4	FALSE	Predicted mitochondrial
P04785	ENSRNOG00000036689	P4hb	8	FALSE	Known mitochondrial
P04906	ENSRNOG00000018237	Gstp1	2	TRUE	Predicted mitochondrial
P05197	ENSRNOG00000020266	Eef2	5	FALSE	Predicted mitochondrial
P05503	ENSRNOG00000034234	Mt-co1	1	TRUE	Known mitochondrial
P05504	ENSRNOG00000031979	Mt-atp6	0	TRUE	Known mitochondrial
P05505	ENSRNOG00000030700	Mt-co3	1	TRUE	Known mitochondrial
P05506	ENSRNOG00000033615	Mt-nd3	0	TRUE	Known mitochondrial
P05982	ENSRNOG00000012772	Nqo1	2	FALSE	Known mitochondrial
P06761	ENSRNOG00000018294	Hspa5	7	TRUE	Known mitochondrial
P07632	ENSRNOG00000002115	Sod1	4	TRUE	Predicted mitochondrial
P07895	ENSRNOG00000019048	Sod2	7	TRUE	Known mitochondrial

P07943	ENSRNOG00000009513	Akr1b1	2	FALSE	Predicted mitochondrial
P08009	ENSRNOG00000018937	Gstm2	3	FALSE	Predicted mitochondrial
P08050	ENSRNOG00000000805	Gja1	1	TRUE	Known mitochondrial
P08461	ENSRNOG00000009994	Dlat	8	TRUE	Known mitochondrial
P09034	ENSRNOG00000008837	Ass1	3	TRUE	Predicted mitochondrial
P09527	ENSRNOG00000012247	Rab7a	4	FALSE	Predicted mitochondrial
P09606	ENSRNOG00000049560	Glul	0	TRUE	Predicted mitochondrial
P09895	ENSRNOG00000023529	Rpl5	1	FALSE	Predicted mitochondrial
P0C2C1	ENSRNOG00000049330	NEWGENE_1308196	2	TRUE	Known mitochondrial
P0C2C4	ENSRNOG00000009567	Mrpl10	3	TRUE	Known mitochondrial
P0DMW0	ENSRNOG00000045654	Hspa1a	2	FALSE	Known mitochondrial
P10818	ENSRNOG00000001170	Cox6a1	5	TRUE	Known mitochondrial
P10860	ENSRNOG00000057367	Glud1	13	TRUE	Known mitochondrial
P10888	ENSRNOG00000017817	Cox4i1	9	TRUE	Known mitochondrial
P11030	ENSRNOG00000046889	Dbi	3	FALSE	Known mitochondrial
P11240	ENSRNOG00000018816	Cox5a	11	TRUE	Known mitochondrial
P11348	ENSRNOG00000003253	Qdpr	4	FALSE	Known mitochondrial
P11951	ENSRNOG00000010807	Cox6c	6	TRUE	Known mitochondrial
P11980	ENSRNOG00000011329	Pkm	5	FALSE	Predicted mitochondrial
P12007	ENSRNOG00000009421	Ivd	6	TRUE	Known mitochondrial
P12075	ENSRNOG00000016660	Cox5b	4	TRUE	Known mitochondrial
P13233	ENSRNOG00000017496	Cnp	3	TRUE	Known mitochondrial
P13803	ENSRNOG00000015233	Etfa	8	TRUE	Known mitochondrial
P14604	ENSRNOG00000018522	Echs1	11	TRUE	Known mitochondrial
P14942	ENSRNOG00000030449	Gsta4	0	TRUE	Predicted mitochondrial
P15650	ENSRNOG00000012966	Acadl	8	TRUE	Known mitochondrial
P16617	ENSRNOG00000058249	Pgk1	3	FALSE	Known mitochondrial
P16970	ENSRNOG00000011929	Abcd3	5	TRUE	Predicted mitochondrial
P17074	ENSRNOG00000037897	NEWGENE_68440	1	FALSE	Predicted mitochondrial
P17764	ENSRNOG00000007862	Acat1	9	TRUE	Known mitochondrial
P18163	ENSRNOG00000010633	Acsl1	10	TRUE	Known mitochondrial
P18297	ENSRNOG00000015455	Spr	4	FALSE	Predicted mitochondrial
P18418	ENSRNOG00000003029	Calr	5	FALSE	Known mitochondrial
P19234	ENSRNOG00000042503	Ndufv2	9	TRUE	Known mitochondrial
P19511	ENSRNOG00000016000	Atp5pb	11	TRUE	Known mitochondrial
P19643	ENSRNOG00000029778	Maob	6	TRUE	Known mitochondrial
P19804	ENSRNOG00000002671	Nme2	5	TRUE	Predicted mitochondrial
P19945	ENSRNOG00000001148	Rplp0	4	FALSE	Predicted mitochondrial
P20070	ENSRNOG00000009592	Cyb5r3	4	TRUE	Known mitochondrial
P20788	ENSRNOG00000018281	Uqcrfs1	10	TRUE	Known mitochondrial
P21531	ENSRNOG00000016896	Rpl3	3	FALSE	Predicted mitochondrial
P21533	ENSRNOG00000025936	Rpl6	4	FALSE	Predicted mitochondrial
P21571	ENSRNOG00000001551	Atp5pf	5	TRUE	Known mitochondrial

P21708	ENSRNOG00000053583	Mapk3	1	TRUE	Predicted mitochondrial
P21913	ENSRNOG00000007967	Sdhb	7	TRUE	Known mitochondrial
P22734	ENSRNOG00000001889	Comt	2	FALSE	Predicted mitochondrial
P23928	ENSRNOG00000010524	Cryab	1	TRUE	Predicted mitochondrial
P24329	ENSRNOG00000000186	Tst	9	TRUE	Known mitochondrial
P24368	ENSRNOG00000016781	Ppib	6	FALSE	Predicted mitochondrial
P24473	ENSRNOG00000016484	Gstk1	5	TRUE	Known mitochondrial
P25235	ENSRNOG00000007492	Rpn2	3	FALSE	Predicted mitochondrial
P26453	ENSRNOG00000008414	Bsg	3	TRUE	Predicted mitochondrial
P27008	ENSRNOG00000003084	Parp1	1	TRUE	Known mitochondrial
P27615	ENSRNOG00000002225	Scarb2	3	FALSE	Predicted mitochondrial
P27881	ENSRNOG00000006116	Hk2	2	TRUE	Known mitochondrial
P29117	ENSRNOG00000010558	Ppif	4	TRUE	Known mitochondrial
P29266	ENSRNOG00000008063	Hibadh	8	TRUE	Known mitochondrial
P29314	ENSRNOG00000058909	Rps9l1	2	FALSE	Predicted mitochondrial
P29418	ENSRNOG00000049912	Atp5f1e	3	TRUE	Known mitochondrial
P29419	ENSRNOG00000000064	Atp5i	6	TRUE	Known mitochondrial
P31399	ENSRNOG00000003626	Atp5h	10	TRUE	Known mitochondrial
P32089	ENSRNOG00000038001	Slc25a1	5	TRUE	Known mitochondrial
P32198	ENSRNOG00000014254	Cpt1a	4	TRUE	Known mitochondrial
P32551	ENSRNOG00000036742	Uqcrc2	13	TRUE	Known mitochondrial
P33124	ENSRNOG00000026745	Acsl6	2	TRUE	Predicted mitochondrial
P34058	ENSRNOG00000019834	Hsp90ab1	4	FALSE	Predicted mitochondrial
P35171	ENSRNOG00000042903	Cox7a2l2	6	TRUE	Known mitochondrial
P35559	ENSRNOG00000016833	Ide	1	TRUE	Known mitochondrial
P35565	ENSRNOG00000003343	Canx	3	FALSE	Known mitochondrial
P36970	ENSRNOG00000013604	Gpx4	1	TRUE	Known mitochondrial
P37377	ENSRNOG00000008656	Snca	0	TRUE	Known mitochondrial
P38718	ENSRNOG00000003150	Mpc2	5	TRUE	Known mitochondrial
P40329	ENSRNOG00000007739	Rars	0	FALSE	Predicted mitochondrial
P41123	ENSRNOG00000015335	Rpl13	2	FALSE	Predicted mitochondrial
P41499	ENSRNOG00000030124	Ptpn11	0	TRUE	Known mitochondrial
P41562	ENSRNOG00000015020	Idh1	3	TRUE	Predicted mitochondrial
P43138	ENSRNOG00000009663	Apex1	1	TRUE	Known mitochondrial
P47196	ENSRNOG00000028629	Akt1	0	TRUE	Known mitochondrial
P48500	ENSRNOG00000015290	Tpi1	7	FALSE	Predicted mitochondrial
P49432	ENSRNOG00000007895	Pdhb	8	TRUE	Known mitochondrial
P50554	ENSRNOG00000002636	Abat	4	TRUE	Known mitochondrial
P51583	ENSRNOG00000002101	Paics	1	FALSE	Known mitochondrial
P52631	ENSRNOG00000019742	Stat3	2	TRUE	Known mitochondrial
P52759	ENSRNOG00000005437	Rida	5	TRUE	Known mitochondrial
P52873	ENSRNOG00000019372	Pc	7	TRUE	Known mitochondrial
P53563	ENSRNOG00000007946	Bcl2l1	2	TRUE	Known mitochondrial

P54311	ENSRNOG00000016638	Gnb1	3	FALSE	Predicted mitochondrial
P54690	ENSRNOG00000015514	Bcat1	1	TRUE	Predicted mitochondrial
P56522	ENSRNOG00000058497	Fdxr	4	TRUE	Known mitochondrial
P56571	ENSRNOG00000001211	RGD1303003	4	TRUE	Known mitochondrial
P56574	ENSRNOG00000013949	Idh2	4	TRUE	Known mitochondrial
P57113	ENSRNOG00000047708	Gstz1	3	TRUE	Known mitochondrial
P60711	ENSRNOG00000034254	Actb	4	FALSE	Known mitochondrial
P60905	ENSRNOG00000015202	Dnajc5	2	FALSE	Predicted mitochondrial
P61206	ENSRNOG00000054775	Fkbp11	1	FALSE	Predicted mitochondrial
P61589	ENSRNOG00000050519	Rhoa	1	FALSE	Predicted mitochondrial
P61765	ENSRNOG00000015420	Stxbp1	2	FALSE	Predicted mitochondrial
P61980	ENSRNOG00000019113	Hnrnpk	3	TRUE	Known mitochondrial
P62074	ENSRNOG00000007883	Timm10	2	TRUE	Known mitochondrial
P62076	ENSRNOG00000019682	Timm13	3	TRUE	Known mitochondrial
P62243	ENSRNOG00000054626	Rps8	2	FALSE	Predicted mitochondrial
P62246	ENSRNOG00000018320	Rps15a	1	FALSE	Predicted mitochondrial
P62250	ENSRNOG00000019578	Rps16	2	FALSE	Predicted mitochondrial
P62260	ENSRNOG00000005290	Ywhae	4	FALSE	Predicted mitochondrial
P62271	ENSRNOG00000033152	Rps18	4	FALSE	Predicted mitochondrial
P62278	ENSRNOG00000028021	LOC684988	2	FALSE	Predicted mitochondrial
P62718	ENSRNOG00000018795	Rpl18a	3	FALSE	Predicted mitochondrial
P62832	ENSRNOG00000004107	Rpl23	4	FALSE	Predicted mitochondrial
P62890	ENSRNOG00000005975	LOC100362027	4	FALSE	Predicted mitochondrial
P62898	ENSRNOG00000010452	Cycs	4	TRUE	Known mitochondrial
P62907	ENSRNOG00000000505	Rpl10a	3	FALSE	Predicted mitochondrial
P62909	ENSRNOG00000017418	Rps3	4	TRUE	Known mitochondrial
P62912	ENSRNOG00000010746	Rpl32	1	FALSE	Predicted mitochondrial
P62919	ENSRNOG00000032635	LOC108348142	3	FALSE	Predicted mitochondrial
P62944	ENSRNOG00000061543	Ap2b1	3	FALSE	Predicted mitochondrial
P63012	ENSRNOG00000019433	Rab3a	2	FALSE	Known mitochondrial
P63036	ENSRNOG00000007029	Dnaja1	2	TRUE	Predicted mitochondrial
P63039	ENSRNOG00000014525	Hspd1	12	TRUE	Known mitochondrial
P63086	ENSRNOG00000001849	Mapk1	1	TRUE	Predicted mitochondrial
P63088	ENSRNOG00000001269	Ppp1cc	1	TRUE	Predicted mitochondrial
P63102	ENSRNOG00000008195	Ywhaz	4	TRUE	Predicted mitochondrial
P63219	ENSRNOG00000015936	LOC108349548	0	FALSE	Predicted mitochondrial
P63245	ENSRNOG00000049070	Rack1	2	FALSE	Predicted mitochondrial
P63322	ENSRNOG00000013454	Rala	2	FALSE	Predicted mitochondrial
P63326	ENSRNOG00000052141	Rps10l1	1	FALSE	Predicted mitochondrial
P67779	ENSRNOG00000046799	Phb	12	TRUE	Known mitochondrial
P68255	ENSRNOG00000051650	Ywhaq	3	FALSE	Predicted mitochondrial
P68370	ENSRNOG00000060728	Tuba1a	2	FALSE	Predicted mitochondrial
P68511	ENSRNOG00000055471	Ywhah	3	FALSE	Predicted mitochondrial

P69897	ENSRNOG00000061216	Tubb5	3	FALSE	Predicted mitochondrial
P70470	ENSRNOG00000008320	Lypla1	2	FALSE	Predicted mitochondrial
P70500	ENSRNOG00000024144	Cdipt	3	FALSE	Predicted mitochondrial
P70549	ENSRNOG00000029871	Slc8a3	0	TRUE	Known mitochondrial
P70583	ENSRNOG00000007221	Dut	2	FALSE	Known mitochondrial
P80254	ENSRNOG00000001239	Ddt	2	FALSE	Predicted mitochondrial
P80432	ENSRNOG00000030237	Cox7c	1	TRUE	Known mitochondrial
P81155	ENSRNOG00000013505	Vdac2	7	TRUE	Known mitochondrial
P82995	ENSRNOG00000007219	Hsp90aa1	4	FALSE	Predicted mitochondrial
P83565	ENSRNOG00000049686	Mrpl40	2	TRUE	Known mitochondrial
P84817	ENSRNOG00000001420	Fis1	5	TRUE	Known mitochondrial
P84850	ENSRNOG00000019012	D2hgdh	4	TRUE	Known mitochondrial
P85834	ENSRNOG00000018604	Tufm	3	TRUE	Known mitochondrial
P97519	ENSRNOG00000009422	Hmgcl	5	TRUE	Known mitochondrial
P97532	ENSRNOG00000000185	Mpst	5	TRUE	Known mitochondrial
P97576	ENSRNOG00000006593	Grpel1	5	TRUE	Known mitochondrial
P97612	ENSRNOG00000011019	Faah	5	FALSE	Predicted mitochondrial
P97615	ENSRNOG00000005614	Txn2	2	TRUE	Known mitochondrial
P97887	ENSRNOG00000009110	Psen1	0	TRUE	Known mitochondrial
Q00972	ENSRNOG00000019485	Bckdk	2	TRUE	Known mitochondrial
Q02356	ENSRNOG00000019240	Ampd2	1	FALSE	Predicted mitochondrial
Q03344	ENSRNOG00000013300	Atp5if1	1	TRUE	Known mitochondrial
Q03346	ENSRNOG00000012693	Pmpcb	6	TRUE	Known mitochondrial
Q04462	ENSRNOG00000000867	Vars	1	FALSE	Predicted mitochondrial
Q05982	ENSRNOG00000002693	Nme1	2	TRUE	Predicted mitochondrial
Q06645	ENSRNOG00000007235	Atp5mc1	0	TRUE	Known mitochondrial
Q06646	ENSRNOG00000015320	Atp5mc2	0	TRUE	Known mitochondrial
Q06647	ENSRNOG00000001991	Atp5po	10	TRUE	Known mitochondrial
Q07803	ENSRNOG00000012873	Gfm1	4	TRUE	Known mitochondrial
Q07936	ENSRNOG00000010362	Anxa2	2	FALSE	Predicted mitochondrial
Q07984	ENSRNOG00000053172	Ssr4	4	FALSE	Predicted mitochondrial
Q08877	ENSRNOG00000026490	Dnm3	0	TRUE	Predicted mitochondrial
Q09073	ENSRNOG00000039980	Slc25a5	8	TRUE	Known mitochondrial
Q0D2L2	ENSRNOG00000047984	Mrps22	1	TRUE	Known mitochondrial
Q0ZFS4	ENSRNOG00000026528	Mrps33	1	TRUE	Known mitochondrial
Q1HCL7	ENSRNOG00000054157	Nadk2	2	TRUE	Known mitochondrial
Q1M168	ENSRNOG00000020407	Atcay	0	TRUE	Predicted mitochondrial
Q2TA68	ENSRNOG00000001717	Opa1	5	TRUE	Known mitochondrial
Q2YDU8	ENSRNOG00000017621	Spns1	0	TRUE	Known mitochondrial
Q32Q54	ENSRNOG00000048309	Uqcc1	1	TRUE	Known mitochondrial
Q3B7D0	ENSRNOG00000001654	Cpox	5	TRUE	Known mitochondrial
Q3B7K9	ENSRNOG00000008463	Rundc3b	0	FALSE	Predicted mitochondrial
Q3B7U9	ENSRNOG00000058359	Fkbp8	2	TRUE	Known mitochondrial

Q3B8N9	ENSRNOG00000017577	Bphl	3	TRUE	Predicted mitochondrial
Q3B8R7	ENSRNOG00000011639	Mrpl47	1	TRUE	Known mitochondrial
Q3KRD0	ENSRNOG00000002813	Dars2	2	TRUE	Known mitochondrial
Q3KRD5	ENSRNOG00000029799	Tomm34	0	TRUE	Known mitochondrial
Q3KRE0	ENSRNOG00000018118	Atad3a	3	TRUE	Known mitochondrial
Q3MHS9	ENSRNOG00000000923	Cct6a	1	FALSE	Predicted mitochondrial
Q3MIE4	ENSRNOG00000020684	Vat1	1	TRUE	Predicted mitochondrial
Q3V5X8	ENSRNOG00000016033	Endog	2	TRUE	Known mitochondrial
Q497B0	ENSRNOG00000027797	Nit2	3	FALSE	Predicted mitochondrial
Q498E0	ENSRNOG00000008090	Txndc12	2	FALSE	Known mitochondrial
Q498T4	ENSRNOG00000018057	Mrpl2	3	TRUE	Known mitochondrial
Q498U3	ENSRNOG00000022837	Slc25a40	0	TRUE	Known mitochondrial
Q499N5	ENSRNOG00000003330	Acsf2	3	TRUE	Known mitochondrial
Q499S2	ENSRNOG00000001596	Atp5mc3	0	FALSE	Known mitochondrial
Q4FZT0	ENSRNOG00000009535	Stoml2	6	TRUE	Known mitochondrial
Q4FZT8	ENSRNOG00000003127	Spryd4	4	FALSE	Known mitochondrial
Q4FZU0	ENSRNOG00000017494	Acp6	3	FALSE	Known mitochondrial
Q4FZX5	ENSRNOG00000016873	Msrb2	1	TRUE	Known mitochondrial
Q4G012	ENSRNOG00000005618	Fmc1	0	TRUE	Known mitochondrial
Q4G064	ENSRNOG00000001171	Coq5	4	TRUE	Known mitochondrial
Q4G067	ENSRNOG00000015231	Mrpl44	2	TRUE	Known mitochondrial
Q4G069	ENSRNOG00000025145	Rmdn1	2	FALSE	Known mitochondrial
Q4G086	ENSRNOG00000009820	Bnip3l	0	TRUE	Known mitochondrial
Q4KLL4	ENSRNOG00000009406	Tm9sf4	1	FALSE	Predicted mitochondrial
Q4KLP0	ENSRNOG00000023587	Dhtkd1	2	TRUE	Known mitochondrial
Q4KLZ1	ENSRNOG00000027087	Tmem186	2	FALSE	Predicted mitochondrial
Q4KM93	ENSRNOG00000025484	Tmem177	2	TRUE	Known mitochondrial
Q4QQT0	ENSRNOG00000011567	Rpusd4	0	TRUE	Known mitochondrial
Q4QQV3	ENSRNOG00000002255	Fam162a	2	TRUE	Known mitochondrial
Q4QR80	ENSRNOG00000010912	Mrps25	3	TRUE	Known mitochondrial
Q4QRB4	ENSRNOG00000017209	Tubb3	2	FALSE	Known mitochondrial
Q4V7E5	ENSRNOG00000018773	Mtrf1l	0	TRUE	Known mitochondrial
Q4V7F3	ENSRNOG00000004001	Osgepl1	0	TRUE	Known mitochondrial
Q4V897	ENSRNOG00000009462	Ccdc90b	2	TRUE	Known mitochondrial
Q4V8B7	ENSRNOG00000015576	Hsdl1	2	TRUE	Known mitochondrial
Q4V8F9	ENSRNOG00000016692	Hsdl2	4	TRUE	Known mitochondrial
Q505J6	ENSRNOG00000042731	Slc25a18	1	TRUE	Known mitochondrial
Q52KJ9	ENSRNOG00000057934	Tmx1	2	FALSE	Known mitochondrial
Q52KK3	ENSRNOG00000039278	Slc25a51	3	TRUE	Known mitochondrial
Q561R9	ENSRNOG00000007829	Lactb2	3	TRUE	Predicted mitochondrial
Q561S0	ENSRNOG00000016470	Ndufa10l1	6	TRUE	Known mitochondrial
Q566R0	ENSRNOG00000020829	Them4	2	TRUE	Known mitochondrial
Q569C9	ENSRNOG00000012186	Golph3	0	TRUE	Known mitochondrial

Q5BJN5	ENSRNOG00000022466	LOC100361898	2	TRUE	Known mitochondrial
Q5BJQ0	ENSRNOG00000043201	Coq8a	3	TRUE	Known mitochondrial
Q5BJR4	ENSRNOG00000015088	Prune2	0	FALSE	Predicted mitochondrial
Q5BJS0	ENSRNOG00000029194	Dhx30	1	TRUE	Known mitochondrial
Q5BJS4	ENSRNOG00000003470	Fundc1	2	TRUE	Known mitochondrial
Q5BJT9	ENSRNOG00000014573	Ckmt1	5	TRUE	Known mitochondrial
Q5BJU7	ENSRNOG00000047476	Wasf1	0	TRUE	Known mitochondrial
Q5BJZ3	ENSRNOG00000026842	Nnt	7	TRUE	Known mitochondrial
Q5BK63	ENSRNOG00000061684	Ndufa9	8	TRUE	Known mitochondrial
Q5D059	ENSRNOG00000019113	Hnrnpk	3	TRUE	Known mitochondrial
Q5EB62	ENSRNOG00000017091	Slc25a46	2	TRUE	Known mitochondrial
Q5EB73	ENSRNOG00000006800	Tmem246	0	FALSE	Predicted mitochondrial
Q5FVI4	ENSRNOG00000018448	LOC100911402	1	FALSE	Predicted mitochondrial
Q5FVL2	ENSRNOG00000017654	Emc8	1	FALSE	Known mitochondrial
Q5FVL8	ENSRNOG00000017993	Abcb10	2	TRUE	Known mitochondrial
Q5FVQ4	ENSRNOG00000021725	Mlec	3	FALSE	Predicted mitochondrial
Q5HZA6	ENSRNOG00000007326	Prepl	2	FALSE	Known mitochondrial
Q5HZA9	ENSRNOG00000022748	Tmem126a	2	TRUE	Known mitochondrial
Q5HZE0	ENSRNOG00000004351	Slc25a29	0	TRUE	Known mitochondrial
Q5HZY2	ENSRNOG00000004820	Sar1b	2	FALSE	Known mitochondrial
Q5I0C5	ENSRNOG00000014602	Mtfmt	0	TRUE	Known mitochondrial
Q5I0D1	ENSRNOG00000007788	Glod4	3	TRUE	Known mitochondrial
Q5I0E7	ENSRNOG00000021882	Tmed9	2	FALSE	Predicted mitochondrial
Q5I0I4	ENSRNOG00000020596	Dmac2	2	TRUE	Known mitochondrial
Q5I0K3	ENSRNOG00000014075	Clybl	1	TRUE	Known mitochondrial
Q5I0K5	ENSRNOG00000043107	Abhd10	2	TRUE	Known mitochondrial
Q5I0K8	ENSRNOG00000003797	Mrps7	3	TRUE	Known mitochondrial
Q5I0L3	ENSRNOG00000025252	Yars2	3	TRUE	Known mitochondrial
Q5I0P2	ENSRNOG00000011535	Gcsh	3	TRUE	Known mitochondrial
Q5M7W7	ENSRNOG00000007327	Pars2	3	TRUE	Known mitochondrial
Q5M818	ENSRNOG00000021005	Mrpl16	2	TRUE	Known mitochondrial
Q5M867	ENSRNOG00000043060	Dnajc4	0	FALSE	Predicted mitochondrial
Q5M949	ENSRNOG00000010332	Nipsnap3b	3	TRUE	Predicted mitochondrial
Q5M9G9	ENSRNOG00000052477	Tbrg4	4	TRUE	Known mitochondrial
Q5M9H2	ENSRNOG00000018114	Acadvl	10	TRUE	Known mitochondrial
Q5M9I5	ENSRNOG00000012550	Uqcrh	2	TRUE	Known mitochondrial
Q5PQN9	ENSRNOG00000008256	Mrpl38	1	TRUE	Known mitochondrial
Q5PQZ9	ENSRNOG00000012383	Ndufc2	3	TRUE	Known mitochondrial
Q5QJC9	ENSRNOG00000011527	Bag5	0	FALSE	Known mitochondrial
Q5RJN0	ENSRNOG00000024568	Ndufs7	3	TRUE	Known mitochondrial
Q5RJR8	ENSRNOG00000003524	Lrrc59	3	FALSE	Known mitochondrial
Q5RJY4	ENSRNOG00000005360	Dhrs7b	3	FALSE	Predicted mitochondrial
Q5RK00	ENSRNOG00000018547	Mrpl46	2	TRUE	Known mitochondrial

Q5RK08	ENSRNOG00000023919	Nipsnap2	1	TRUE	Known mitochondrial
Q5RK17	ENSRNOG00000029197	Diablo	2	TRUE	Known mitochondrial
Q5RKH7	ENSRNOG00000009459	Slc35f6	0	TRUE	Known mitochondrial
Q5RKI8	ENSRNOG00000008557	Abcb8	2	TRUE	Known mitochondrial
Q5RKI9	ENSRNOG00000025997	Mrrf	1	TRUE	Known mitochondrial
Q5RKJ9	ENSRNOG00000047088	Rab10	4	FALSE	Predicted mitochondrial
Q5U1W6	ENSRNOG00000004512	Apool	3	TRUE	Known mitochondrial
Q5U1X1	ENSRNOG00000018939	Rexo2	1	TRUE	Known mitochondrial
Q5U1Z8	ENSRNOG00000006858	Pyurf	0	TRUE	Known mitochondrial
Q5U1Z9	ENSRNOG00000001559	Mtx2	3	TRUE	Known mitochondrial
Q5U204	ENSRNOG00000010552	Lamtor3	2	FALSE	Predicted mitochondrial
Q5U2N0	ENSRNOG00000004257	Ctps2	0	FALSE	Predicted mitochondrial
Q5U2Q7	ENSRNOG00000019450	Etf1	0	FALSE	Predicted mitochondrial
Q5U2R1	ENSRNOG00000042962	Pdss2	1	TRUE	Known mitochondrial
Q5U2R4	ENSRNOG00000039567	Trmt10c	2	TRUE	Known mitochondrial
Q5U2R6	ENSRNOG00000017133	LOC306766	0	FALSE	Predicted mitochondrial
Q5U2T7	ENSRNOG00000002695	Tfb2m	1	TRUE	Known mitochondrial
Q5U2U0	ENSRNOG00000030225	Clpx	4	TRUE	Known mitochondrial
Q5U2V5	ENSRNOG00000021273	Crls1	0	TRUE	Known mitochondrial
Q5U2X7	ENSRNOG00000015142	Timm21	2	TRUE	Known mitochondrial
Q5U2X8	ENSRNOG00000003782	Acot9	2	FALSE	Known mitochondrial
Q5U300	ENSRNOG00000019164	Uba1	1	TRUE	Known mitochondrial
Q5U316	ENSRNOG00000022014	Rab35	2	FALSE	Known mitochondrial
Q5U3Z5	ENSRNOG00000000979	Bri3bp	0	TRUE	Predicted mitochondrial
Q5U3Z7	ENSRNOG00000008106	Shmt2	3	TRUE	Known mitochondrial
Q5XFW4	ENSRNOG00000004401	Mrpl13	2	TRUE	Known mitochondrial
Q5XHZ0	ENSRNOG00000005418	Trap1	4	TRUE	Known mitochondrial
Q5XI37	ENSRNOG00000008279	Mrps15	2	TRUE	Known mitochondrial
Q5XI78	ENSRNOG00000005130	LOC103693780	7	TRUE	Known mitochondrial
Q5XI79	ENSRNOG00000005279	Ndufaf7	2	TRUE	Known mitochondrial
Q5XI86	ENSRNOG00000004288	Ptrh2	2	TRUE	Known mitochondrial
Q5XIA5	ENSRNOG00000019918	Coasy	3	TRUE	Known mitochondrial
Q5XIA8	ENSRNOG00000013961	Ghitm	1	TRUE	Known mitochondrial
Q5XIC0	ENSRNOG00000029549	Eci2	5	TRUE	Known mitochondrial
Q5XIC2	ENSRNOG00000014128	Ecsit	3	TRUE	Known mitochondrial
Q5XID7	ENSRNOG00000025730	Armcx3	2	TRUE	Known mitochondrial
Q5XIE2	ENSRNOG00000006978	Mterf2	1	TRUE	Known mitochondrial
Q5XIE6	ENSRNOG00000028557	Hibch	4	TRUE	Known mitochondrial
Q5XIF3	ENSRNOG00000011383	Ndufs4	4	TRUE	Known mitochondrial
Q5XIG0	ENSRNOG00000002186	Nudt9	2	TRUE	Known mitochondrial
Q5XIG4	ENSRNOG00000002205	Ociad1	2	FALSE	Known mitochondrial
Q5XIG9	ENSRNOG00000004640	Mtfp1	2	TRUE	Known mitochondrial
Q5XIH3	ENSRNOG00000018117	Ndufv1	5	TRUE	Known mitochondrial

Q5XIH4	ENSRNOG00000002759	Lias	0	TRUE	Known mitochondrial
Q5XIH7	ENSRNOG00000012999	Phb2	7	TRUE	Known mitochondrial
Q5XII0	ENSRNOG00000060141	Epdr1	1	FALSE	Predicted mitochondrial
Q5XII9	ENSRNOG00000016937	Mtfr1l	1	TRUE	Known mitochondrial
Q5XIJ3	ENSRNOG00000055572	Idh3g	4	TRUE	Known mitochondrial
Q5XIJ4	ENSRNOG00000016740	Fam210a	1	TRUE	Predicted mitochondrial
Q5XIK2	ENSRNOG00000005308	Tmx2	1	FALSE	Predicted mitochondrial
Q5XIM0	ENSRNOG00000016754	Bcs1l	2	TRUE	Known mitochondrial
Q5XIM4	ENSRNOG00000004893	Atp5s	2	TRUE	Known mitochondrial
Q5XIM7	ENSRNOG00000019456	Kars	0	TRUE	Known mitochondrial
Q5XIN6	ENSRNOG00000016427	Letm1	4	TRUE	Known mitochondrial
Q5XIT9	ENSRNOG00000017752	Mccc2	2	TRUE	Known mitochondrial
Q5XIU9	ENSRNOG00000014051	Pgrmc2	3	FALSE	Predicted mitochondrial
Q5XIW0	ENSRNOG00000019811	Timm23	1	TRUE	Known mitochondrial
Q5XJW2	ENSRNOG00000003011	Gadd45gip1	1	TRUE	Known mitochondrial
Q60587	ENSRNOG00000010800	Hadhb	12	TRUE	Known mitochondrial
Q62636	ENSRNOG00000007048	Rap1b	3	FALSE	Predicted mitochondrial
Q62651	ENSRNOG00000020308	Ech1	7	TRUE	Known mitochondrial
Q62703	ENSRNOG00000015780	Rcn2	2	FALSE	Known mitochondrial
Q62760	ENSRNOG00000019980	Tomm20	2	TRUE	Known mitochondrial
Q63042	ENSRNOG00000013370	Gfer	1	TRUE	Known mitochondrial
Q63151	ENSRNOG00000014718	Acsl3	1	TRUE	Known mitochondrial
Q63159	ENSRNOG00000009974	Coq3	4	TRUE	Known mitochondrial
Q63327	ENSRNOG00000018700	Mobp	0	TRUE	Known mitochondrial
Q63362	ENSRNOG00000005698	Ndufa5	6	TRUE	Known mitochondrial
Q63584	ENSRNOG00000007901	Tmed10	3	FALSE	Predicted mitochondrial
Q63666	ENSRNOG00000019219	Vamp1	0	TRUE	Known mitochondrial
Q63707	ENSRNOG00000015063	Dhodh	4	TRUE	Known mitochondrial
Q63716	ENSRNOG00000017194	Prdx1	4	TRUE	Predicted mitochondrial
Q63750	ENSRNOG00000020354	Mrpl23	2	TRUE	Known mitochondrial
Q64057	ENSRNOG00000014645	Aldh7a1	4	TRUE	Known mitochondrial
Q641X9	ENSRNOG00000020869	mrpl9	2	TRUE	Known mitochondrial
Q641Y0	ENSRNOG00000015079	Ddost	3	FALSE	Predicted mitochondrial
Q641Y2	ENSRNOG00000038372	Ndufs2	5	TRUE	Known mitochondrial
Q641Z9	ENSRNOG00000003163	Sdhc	6	TRUE	Known mitochondrial
Q64232	ENSRNOG00000021808	Tecr	3	FALSE	Predicted mitochondrial
Q64244	ENSRNOG00000003069	Cd38	0	FALSE	Known mitochondrial
Q642A4	ENSRNOG00000045765	MGC94207	2	FALSE	Known mitochondrial
Q642E6	ENSRNOG00000019212	Tpp1	2	FALSE	Predicted mitochondrial
Q64428	ENSRNOG00000024629	Hadha	13	TRUE	Known mitochondrial
Q64559	ENSRNOG00000010580	Acot7	2	FALSE	Known mitochondrial
Q66H15	ENSRNOG00000011690	Rmdn3	2	TRUE	Known mitochondrial
Q66H47	ENSRNOG00000022234	mrpl24	3	TRUE	Known mitochondrial

Q66HA6	ENSRNOG00000055860	Arl8b	3	FALSE	Predicted mitochondrial
Q66HF1	ENSRNOG00000011849	Ndufs1	12	TRUE	Known mitochondrial
Q66HF3	ENSRNOG00000009538	Etfdh	4	TRUE	Known mitochondrial
Q66HG9	ENSRNOG00000025295	Mavs	2	TRUE	Known mitochondrial
Q66HI5	ENSRNOG00000022619	Fth1	1	FALSE	Predicted mitochondrial
Q66HP8	ENSRNOG00000020288	Slc25a20	5	TRUE	Known mitochondrial
Q66X93	ENSRNOG00000031173	Snd1	1	FALSE	Predicted mitochondrial
Q68FR6	ENSRNOG00000020075	Eef1g	2	FALSE	Predicted mitochondrial
Q68FS4	ENSRNOG00000003289	Lap3	4	FALSE	Known mitochondrial
Q68FT1	ENSRNOG00000016190	Coq9	5	TRUE	Known mitochondrial
Q68FT3	ENSRNOG00000015807	Pyroxd2	3	FALSE	Predicted mitochondrial
Q68FU3	ENSRNOG00000017851	Etfb	5	TRUE	Known mitochondrial
Q68FU7	ENSRNOG00000011164	Coq6	2	TRUE	Known mitochondrial
Q68FW7	ENSRNOG00000057194	Tars2	2	TRUE	Known mitochondrial
Q68FX0	ENSRNOG00000007316	Idh3B	4	TRUE	Known mitochondrial
Q68FX9	ENSRNOG00000017866	Sirt5	2	TRUE	Known mitochondrial
Q68FY0	ENSRNOG00000032134	Uqcrc1	10	TRUE	Known mitochondrial
Q68FZ8	ENSRNOG00000015869	Pccb	5	FALSE	Known mitochondrial
Q68G41	ENSRNOG00000008843	Eci1	10	FALSE	Known mitochondrial
Q6AXQ5	ENSRNOG00000059442	Pde12	2	TRUE	Known mitochondrial
Q6AXT0	ENSRNOG00000009078	Mrpl37	3	TRUE	Known mitochondrial
Q6AXT5	ENSRNOG00000003923	Rab21	2	FALSE	Predicted mitochondrial
Q6AXV4	ENSRNOG00000011952	Samm50	4	TRUE	Known mitochondrial
Q6AXW1	ENSRNOG00000003385	Glrx2	2	TRUE	Known mitochondrial
Q6AXX6	ENSRNOG00000011140	Fam213a	2	FALSE	Predicted mitochondrial
Q6AXY8	ENSRNOG00000020264	Dhrs1	2	FALSE	Predicted mitochondrial
Q6AY04	ENSRNOG00000015983	Maip1	3	TRUE	Known mitochondrial
Q6AY19	ENSRNOG00000020848	Coq8b	2	TRUE	Known mitochondrial
Q6AY23	ENSRNOG00000003267	Pycr2	2	TRUE	Known mitochondrial
Q6AY30	ENSRNOG00000037984	LOC103689999	4	FALSE	Known mitochondrial
Q6AY49	ENSRNOG00000013061	Tusc3	0	TRUE	Predicted mitochondrial
Q6AY55	ENSRNOG00000021682	Dcakd	3	FALSE	Predicted mitochondrial
Q6AY94	ENSRNOG00000002999	Timmdc1	2	TRUE	Known mitochondrial
Q6AYA6	ENSRNOG00000036666	LOC619574	2	FALSE	Predicted mitochondrial
Q6AYA7	ENSRNOG00000022273	Rfk	0	FALSE	Predicted mitochondrial
Q6AYD9	ENSRNOG00000059386	Nudt19	2	FALSE	Known mitochondrial
Q6AYE2	ENSRNOG00000012957	Sh3glb1	1	TRUE	Known mitochondrial
Q6AYG2	ENSRNOG00000002026	Dnajc28	0	FALSE	Predicted mitochondrial
Q6AYG5	ENSRNOG00000011622	Echdc1	1	TRUE	Known mitochondrial
Q6AYL0	ENSRNOG00000003918	Slc25a19	1	TRUE	Known mitochondrial
Q6AYN4	ENSRNOG00000000274	Phyhipl	0	FALSE	Predicted mitochondrial
Q6AYQ3	ENSRNOG00000016135	Fars2	0	TRUE	Known mitochondrial
Q6AYQ8	ENSRNOG00000014727	Fahd1	4	TRUE	Known mitochondrial

Q6AYS2	ENSRNOG00000018279	Sfxn1	5	TRUE	Known mitochondrial
Q6GMM8	ENSRNOG00000018170	Slc27a1	1	TRUE	Predicted mitochondrial
Q6IMX3	ENSRNOG00000001177	Acads	8	TRUE	Known mitochondrial
Q6IMX8	ENSRNOG00000010134	Acot2	5	FALSE	Known mitochondrial
Q6IMZ3	ENSRNOG00000010668	Anxa6	2	TRUE	Predicted mitochondrial
Q6IRH7	ENSRNOG00000019693	Clpb	3	FALSE	Known mitochondrial
Q6MG61	ENSRNOG00000029682	Clic1	2	TRUE	Predicted mitochondrial
Q6MGB5	ENSRNOG00000000466	Hsd17b8	4	TRUE	Known mitochondrial
Q6P136	ENSRNOG00000010944	Hyou1	4	FALSE	Predicted mitochondrial
Q6P2A5	ENSRNOG00000052506	Ak3	5	TRUE	Known mitochondrial
Q6P3V9	ENSRNOG00000009378	Rpl4	2	FALSE	Predicted mitochondrial
Q6P6R2	ENSRNOG00000006364	Dld	6	TRUE	Known mitochondrial
Q6P792	ENSRNOG00000000875	Fhl1	0	FALSE	Known mitochondrial
Q6P7A7	ENSRNOG00000046345	Rpn1	4	FALSE	Predicted mitochondrial
Q6P7S1	ENSRNOG00000010034	Asah1	3	FALSE	Predicted mitochondrial
Q6P9Y4	ENSRNOG00000010830	Slc25a4	7	TRUE	Known mitochondrial
Q6PCT8	ENSRNOG00000022980	Sdhd	1	TRUE	Known mitochondrial
Q6PCU0	ENSRNOG00000019223	Atp5c1	12	FALSE	Predicted mitochondrial
Q6PCU8	ENSRNOG00000001182	Ndufv3	2	TRUE	Known mitochondrial
Q6PDU2	ENSRNOG00000019511	Mrps18a	1	TRUE	Known mitochondrial
Q6PDU7	ENSRNOG00000028884	Atp5mg	3	TRUE	Known mitochondrial
Q6PDV7	ENSRNOG00000056765	Rpl10	2	FALSE	Predicted mitochondrial
Q6PDW6	ENSRNOG00000019497	Mrpl17	3	TRUE	Known mitochondrial
Q6PEC0	ENSRNOG00000013110	Nudt2	1	FALSE	Predicted mitochondrial
Q6PEC3	ENSRNOG00000020616	LOC103689986	1	FALSE	Predicted mitochondrial
Q6Q0N3	ENSRNOG00000018358	Nt5dc2	1	FALSE	Known mitochondrial
Q6T487	ENSRNOG00000056756	Actn1	4	FALSE	Predicted mitochondrial
Q6TA25	ENSRNOG00000001811	Fgfr1op2	0	FALSE	Predicted mitochondrial
Q6TXE9	ENSRNOG00000002393	Eprs	1	FALSE	Predicted mitochondrial
Q6TXF3	ENSRNOG00000046889	Dbi	3	FALSE	Known mitochondrial
Q6UPE0	ENSRNOG00000015859	Chdh	7	TRUE	Known mitochondrial
Q6V7V2	ENSRNOG00000009022	LOC103692165	0	FALSE	Predicted mitochondrial
Q704S8	ENSRNOG00000018145	Crat	3	TRUE	Known mitochondrial
Q75Q41	ENSRNOG00000014058	Tomm22	5	TRUE	Known mitochondrial
Q76MV3	ENSRNOG00000038951	Cox17	0	TRUE	Known mitochondrial
Q7M0E7	ENSRNOG00000019734	Mrpl14	2	TRUE	Known mitochondrial
Q7TP08	ENSRNOG00000018662	Amacr	3	TRUE	Known mitochondrial
Q7TP77	ENSRNOG00000020975	Mrpl49	2	TRUE	Known mitochondrial
Q7TQ16	ENSRNOG00000048174	Uqcrq	5	TRUE	Known mitochondrial
Q7TQ94	ENSRNOG00000003881	Nit1	2	TRUE	Known mitochondrial
Q7TS56	ENSRNOG00000024411	Cbr4	3	TRUE	Known mitochondrial
Q7TS62	ENSRNOG00000007946	Bcl2l1	2	TRUE	Known mitochondrial
Q7TSA0	ENSRNOG00000019930	Rhot2	2	TRUE	Known mitochondrial

Q7TT47	ENSRNOG00000015150	Spg7	2	TRUE	Known mitochondrial
Q80W89	ENSRNOG00000048320	Ndufa11	3	TRUE	Known mitochondrial
Q8CFD0	ENSRNOG00000037871	Sfxn5	2	TRUE	Known mitochondrial
Q8K3P6	ENSRNOG00000014338	Slc25a25	3	TRUE	Known mitochondrial
Q8VH49	ENSRNOG00000019428	Higd1a	1	TRUE	Known mitochondrial
Q8VHI8	ENSRNOG00000020753	Bnip1	0	TRUE	Known mitochondrial
Q8VID1	ENSRNOG00000018239	Dhrs4	5	TRUE	Predicted mitochondrial
Q91Y94	ENSRNOG00000007827	Cox4i2	0	TRUE	Known mitochondrial
Q91ZW6	ENSRNOG00000000729	Tmlhe	3	TRUE	Known mitochondrial
Q920F5	ENSRNOG00000014522	Mlycd	3	TRUE	Known mitochondrial
Q920L2	ENSRNOG00000013331	Sdha	13	TRUE	Known mitochondrial
Q920P0	ENSRNOG00000050315	Dcxr	2	FALSE	Known mitochondrial
Q924S1	ENSRNOG00000017731	Agpat4	2	FALSE	Known mitochondrial
Q924S5	ENSRNOG00000046502	Lonp1	4	TRUE	Known mitochondrial
Q99JD5	ENSRNOG00000015514	Bcat1	1	TRUE	Predicted mitochondrial
Q99MC0	ENSRNOG00000020676	Ppp1r14a	0	FALSE	Predicted mitochondrial
Q9EP88	ENSRNOG00000006871	Slc25a14	0	TRUE	Known mitochondrial
Q9EPJ3	ENSRNOG00000021224	Mrps26	1	TRUE	Known mitochondrial
Q9EPX4	ENSRNOG00000013902	P2ry12	0	TRUE	Known mitochondrial
Q9EQX9	ENSRNOG00000058053	Ube2n	1	FALSE	Predicted mitochondrial
Q9ER34	ENSRNOG00000024128	Aco2	10	TRUE	Known mitochondrial
Q9ES21	ENSRNOG00000005149	Sacm1l	3	FALSE	Predicted mitochondrial
Q9ET09	ENSRNOG00000017545	Mrs2	1	TRUE	Known mitochondrial
Q9ET45	ENSRNOG00000017243	Bnip3	0	TRUE	Known mitochondrial
Q9JI56	ENSRNOG00000001867	Snap29	1	FALSE	Known mitochondrial
Q9JJW3	ENSRNOG00000020296	LOC103693430	5	TRUE	Known mitochondrial
Q9JK59	ENSRNOG00000000485	Bak1	1	TRUE	Known mitochondrial
Q9JKL3	ENSRNOG00000020876	Bax	2	TRUE	Known mitochondrial
Q9JKW1	ENSRNOG00000007988	Timm22	2	TRUE	Known mitochondrial
Q9JM53	ENSRNOG00000006067	Aifm1	9	TRUE	Known mitochondrial
Q9NQR8	ENSRNOG00000007506	Ndufaf4	6	TRUE	Known mitochondrial
Q9QVC8	ENSRNOG00000006444	Fkbp4	1	TRUE	Known mitochondrial
Q9R1B1	ENSRNOG00000050846	Timm10b	0	TRUE	Known mitochondrial
Q9R1R4	ENSRNOG00000055779	Tdrd7	0	TRUE	Known mitochondrial
Q9WUD9	ENSRNOG00000009495	Src	1	TRUE	Known mitochondrial
Q9WUH4	ENSRNOG00000000875	Fhl1	0	FALSE	Known mitochondrial
Q9WUS0	ENSRNOG00000045738	Ak4	6	TRUE	Known mitochondrial
Q9WV97	ENSRNOG00000008222	Timm9	1	TRUE	Known mitochondrial
Q9WVJ4	ENSRNOG00000006399	Synj2bp	5	TRUE	Known mitochondrial
Q9WVK7	ENSRNOG00000010697	Hadh	8	TRUE	Known mitochondrial
Q9Z0V5	ENSRNOG00000003763	Prdx4	4	FALSE	Known mitochondrial
Q9Z158	ENSRNOG00000005801	Stx17	0	TRUE	Known mitochondrial
Q9Z269	ENSRNOG00000005331	Vapb	2	FALSE	Predicted mitochondrial

Q9Z2L0	ENSRNOG00000006375	Vdac1	9	TRUE	Known mitochondrial

Supplementary Table S2 Mitochondrial DEMPs both belong of the model vs sham-operation group and SG vs model group in rat brain tissue

UniProt Accession	Gene Description	Model vs Sham	SG vs Model
A0A0A0MXZ0	iron-sulfur cluster assembly 1(Isca1)	↑	↑
A0A0G2JVH4	inner membrane mitochondrial protein(Immt)	↑	↓
A0A0G2JYU2	mitochondrial ribosomal protein L11(mrpl11)	↑	↓
A0A0G2JZ68	transcription factor B1, mitochondrial(Tfb1m)	↑	↑
A0A0G2K0L0	3'-phosphoadenosine 5'-phosphosulfate synthase 1(Papss1)	↓	↑
A0A0G2K3V4	O-linked N-acetylglucosamine (GlcNAc) transferase(Ogt)	↓	↑
A0A0G2K459	mitochondrial carrier 2(Mtch2)	↑	↑
A0A0G2KAN7	glutaminase(Gls)	↑	↓
A0A0H2UHK3	FAST kinase domains 2(Fastkd2)	↑	↑
A4F267	translocase of outer mitochondrial membrane 40 like(Tomm40l)	↑	↑
B0BN30	mitochondrial carrier 1(Mtch1)	↑	↓
B0BNM1	NAD(P)HX epimerase(Naxe)	↑	↑
B1WC37	tRNA mitochondrial 2-thiouridylase(Trmu)	↑	↑
B1WC67	solute carrier family 25 member 24(Slc25a24)	↑	↓
B2GUZ6	reticulon 4 interacting protein 1(Rtn4ip1)	↑	↓
B2GV53	solute carrier family 25 member 32(Slc25a32)	↑	↑
B2GV57	cysteinyl-tRNA synthetase 2, mitochondrial(Cars2)	↑	↑
B2RYS8	NADH:ubiquinone oxidoreductase subunit B8(Ndufb8)	↑	↓
D3ZF03	apoptosis inducing factor, mitochondria associated 3(Aifm3)	↓	↑
D3ZFQ3	nucleotide binding protein-like(Nubpl)	↑	↓
D3ZHD3	PET100 cytochrome c oxidase chaperone(Pet100)	↑	↓
D3ZHF8	GTP binding elongation factor GUF1(Guf1)	↑	↑
D3ZL85	holocytochrome c synthase(Hccs)	↑	↓
D3ZLU4	tRNA mitochondrial 2-thiouridylase(Trmu)	↑	↑
D3ZM09	seryl-tRNA synthetase 2, mitochondrial(Sars2)	↑	↑
D3ZNK1	metaxin 3(Mtx3)	↑	↓
D3ZPF2	malonyl-CoA-acyl carrier protein transacylase(Mcat)	↑	↑
D3ZQD3	oxoglutarate dehydrogenase L(Ogdhl)	↓	↑
D3ZVS2	L-2-hydroxyglutarate dehydrogenase(L2hgdh)	↑	↓
D3ZWW5	solute carrier family 30 member 9(Slc30a9)	↑	↑
D4A414	cytochrome c oxidase assembly homolog COX15(Cox15)	↑	↓
F1LU71	AU RNA binding methylglutaconyl-CoA hydratase(Auh)	↑	↓
F1MAR6	proline dehydrogenase 1(Prodh1)	↑	↓
F7EPE0	prosaposin(Psap)	↑	↓
G3V6D3	ATP synthase F1 subunit beta(Atp5f1b)	↑	↓

G3V7J0	aldehyde dehydrogenase 6 family, member A1(Aldh6a1)	↑	↓
G3V8C3	vimentin(Vim)	↑	↓
G3V913	heat shock protein family B (small) member 1(Hspb1)	↑	↓
G3V985	synthesis of cytochrome C oxidase 1(Sco1)	↑	↓
O35567	5-aminoimidazole-4-carboxamide ribonucleotide formyltransferase/IMP cyclohydrolase(Atic)	↓	↑
P00173	cytochrome b5 type A(Cyb5a)	↑	↓
P05505	cytochrome c oxidase III, mitochondrial(mt-Co3)	↑	↑
P07632	superoxide dismutase 1(Sod1)	↓	↓
P08461	dihydrolipoamide S-acetyltransferase(Dlat)	↑	↑
P09034	argininosuccinate synthase 1(Ass1)	↓	↑
P11030	diazepam binding inhibitor(Dbi)	↑	↑
P11240	cytochrome c oxidase subunit 5A(Cox5a)	↑	↓
P11348	quinoid dihydropteridine reductase(Qdpr)	↑	↑
P18297	sepiapterin reductase(Spr)	↑	↑
P21533	ribosomal protein L6(Rpl6)	↑	↑
P23928	crystallin, alpha B(Cryab)	↑	↑
P29266	3-hydroxyisobutyrate dehydrogenase(Hibadh)	↑	↓
P48500	triosephosphate isomerase 1(Tpi1)	↓	↑
P56522	ferredoxin reductase(Fdxr)	↑	↑
P56571	glutamine amidotransferase class 1 domain containing 3A(Gatd3a)	↑	↓
P61589	ras homolog family member A(Rhoa)	↓	↑
P62260	tyrosine 3-monooxygenase/tryptophan 5-monooxygenase activation protein, epsilon(Ywhae)	↓	↓
P63086	mitogen activated protein kinase 1(Mapk1)	↓	↓
P80254	D-dopachrome tautomerase(Ddt)	↓	↑
P84817	fission, mitochondrial 1(Fis1)	↑	↓
Q07936	annexin A2(Anxa2)	↑	↓
Q08877	dynamin 3(Dnm3)	↓	↑
Q2YDU8	sphingolipid transporter 1(Spns1)	↓	↓
Q3MHS9	chaperonin containing TCP1 subunit 6A(Cct6a)	↓	↓
Q4G067	mitochondrial ribosomal protein L44(Mrpl44)	↑	↓
Q4KLP0	dehydrogenase E1 and transketolase domain containing 1(Dhtkd1)	↓	↑
Q4V8F9	hydroxysteroid dehydrogenase like 2(Hsdl2)	↑	↑
Q5I0K3	citramalyl-CoA lyase(Clybl)	↑	↓
Q5U1Z8	PIGY upstream open reading frame(Pyurf)	↑	↑
Q5U3Z5	Bri3 binding protein(Bri3bp)	↑	↑
Q5U3Z7	serine hydroxymethyltransferase 2(Shmt2)	↑	↓
Q5XIH4	lipoic acid synthetase(Lias)	↑	↓
Q5XIT9	methylcrotonyl-CoA carboxylase subunit 2(Mccc2)	↑	↑
Q641Y2	NADH:ubiquinone oxidoreductase core subunit S2(Ndufs2)	↑	↓
Q64428	hydroxyacyl-CoA dehydrogenase trifunctional multienzyme complex subunit alpha(Hadha)	↑	↓

Q66HF1	NADH:ubiquinone oxidoreductase core subunit S1(Ndufs1)	↑	↓
Q68FY0	ubiquinol-cytochrome c reductase core protein 1(Uqcrc1)	↑	↓
Q6AY23	pyrroline-5-carboxylate reductase 2(Pycr2)	↑	↓
Q6AYG2	DnaJ heat shock protein family (Hsp40) member C28(Dnajc28)	↑	↓
Q6AYQ8	fumarylacetoacetate hydrolase domain containing 1(Fahd1)	↑	↑
Q6IMZ3	annexin A6(Anxa6)	↓	↓
Q6MGB5	hydroxysteroid (17-beta) dehydrogenase 8(Hsd17b8)	↑	↓
Q6PCU8	NADH:ubiquinone oxidoreductase subunit V3(Ndufv3)	↑	↓
Q6TXF3	diazepam binding inhibitor(Dbi)	↓	↓
Q9EPX4	purinergic receptor P2Y12(P2ry12)	↑	↓

Supplementary Table S3 The identified peptides associated with the PDK2 binding pockets

Protein Accession	Peptide	Start	End	Binding pocket	t.test.
Q15119|PDK2_HUMAN	K.NASLAGAPKYIEHFSKFSPSP.L	10	30	Lipoamide-binding	p＜0.05
Q15119|PDK2_HUMAN	K.NASLAGAPKYIEHFSKFSPSPLSMK.Q	10	34	Lipoamide-binding	p＜0.05
Q15119|PDK2_HUMAN	K.YIEHFSKFSPSPLSMK.Q	19	34	Lipoamide-binding	p＜0.05
Q15119|PDK2_HUMAN	K.YIEHFSKFSP.S	19	28	Lipoamide-binding	p＜0.05
Q15119|PDK2_HUMAN	K.YIEHFSKFSPSPLSMKQ.F	19	35	Lipoamide-binding	p＜0.05
Q15119|PDK2_HUMAN	K.YIEHFSKFSPSPL.S	19	31	Lipoamide-binding	p＜0.05
Q15119|PDK2_HUMAN	S.KFSPSPLSMK.Q	25	34	Lipoamide-binding	p＜0.05
Q15119|PDK2_HUMAN	K.FSPSPLSM.K	26	33	Lipoamide-binding	p＜0.05
Q15119|PDK2_HUMAN	K.FSPSPLSM(+15.99)K.Q	26	34	Lipoamide-binding	p＜0.05
Q15119|PDK2_HUMAN	K.FSPSPLSMKQFLDFGSSNAC(+57.02)EK.T	26	47	Lipoamide-binding	ns
Q15119|PDK2_HUMAN	K.FSPSPLSMKQFLDFGSSNAC(+57.02)E.K	26	46	Lipoamide-binding	p＜0.05
Q15119|PDK2_HUMAN	K.QFLDFGSSNAC(+57.02)EK.T	35	47	Lipoamide-binding	p＜0.05
Q15119|PDK2_HUMAN	R.HNDVVPTMAQGVLE.Y	123	136	CoA-binding	ns
Q15119|PDK2_HUMAN	K.DTYGDDPVSNQNIQYFLDR.F	139	157	CoA-binding	ns
Q15119|PDK2_HUMAN	K.IERLFSYMYSTAPTPQPGTGGTPLAGFGYGLP.I	300	331	ATP-binding	p＜0.05
Q15119|PDK2_HUMAN	R.LFSYM(+15.99)YSTAPTPQPGTGGT.P	303	321	ATP-binding	ns
Q15119|PDK2_HUMAN	R.LFSYM(+15.99)YSTA.P	303	311	ATP-binding	p＜0.05
Q15119|PDK2_HUMAN	R.LFSYM(+15.99)YSTAPTPQPGTGGTPLAGFG.Y	303	327	ATP-binding	p＜0.05
Q15119|PDK2_HUMAN	R.LFSYMYSTA.P	303	311	ATP-binding	p＜0.05
Q15119|PDK2_HUMAN	L.AGFGYGLPISR.L	324	334	ATP-binding	p＜0.05
Q15119|PDK2_HUMAN	G.FGYGLPISR.L	326	334	ATP-binding	p＜0.05
